# Microwave irradiated synthesis of Schiff bases of 4-(arylideneamino)-5-alkyl-2,4-dihydro-1,2,4-triazole-3-thione containing 1,2,4-triazole segment

**DOI:** 10.3906/kim-2105-39

**Published:** 2021-07-18

**Authors:** Mahdi SHIRMOHAMMADI, Saeid SOUZANGARZADEH, Dadkhoda GHAZANFARI, Mohsen GHODRATBEIGI, Mohammad Reza AKHGAR

**Affiliations:** 1 Department of Chemistry, Kerman branch, Islamic Azad University, Kerman, Iran; 2 Department of Chemistry, Faculty of Basic Science, Yadegare Imam Khomeini (RAH) Shahr-e Rey Branch, Islamic Azad University, Tehran, Iran

**Keywords:** 4-Amino-5-alkyl-4H-1, 2, 4-triazole-3-thione, thiocarbohydrazide, long-chain aliphatic carboxylic acids, Schiff bases

## Abstract

Novel compounds based on the 1,2,4-triazole skeleton were synthesized. A class of 4-amino-5-alkyl-4H-1,2,4-triazole-3-thione created by reaction of thiocarbohydrazide with long-chain aliphatic carboxylic acids, and then the Schiff bases were obtained in the media of heat and microwave waves, in the presence and the absence of a catalyst. Their chemical structures were assayed by elemental analysis, also device spectroscopic methods.

## 1. Introduction

Over the past decades, heterocyclic compounds and their various derivatives have attracted chemists due to their diverse applications in chemical and pharmaceutical fields. Review references indicate that triazole compounds are of particular importance to other heterocyclic compounds due to their biological properties. 1,2,4-triazoles exhibit a variety of biological properties, such as antimicrobial [1–5], antiinflammatory [6–8], anticonvulsant [9,10], anticancer [11,12,14,15], antitubercular [13], antibacterial [1,7,16–19], antifungal [1,20–22], antitubulin [23], insecticides [24], herbicidal [25] and anticorrosion [26] activities. Due to potential properties of triazoles and the fact that one of the tasks of our research team is to investigate industrial emulsifiers, we expect such compounds to have emulsifying properties because they have a polar head and a long nonpolar tail. So, we decided to make compounds that have such characteristics in addition to being new.

We succeeded to synthesize the structures of 4a–f and 5a–l using thiocarbohydrazide.

## 2. Materials and methods

Solvents and analytical chemicals used were of analytical grade or dry distilled. The qualitative analysis of compounds was evaluated by TLC, and the Rf values were assessed using prefabricated aluminum-silicon plates and Kieselgel 60 F254 (obtained from Merck) by using ethyl acetate as a molecule and the TLC which then visualized by means of a UV lamp. Determining melting points was performed using Electrothermal melting furnace (B1 4300 BAMSETEP B1). Bruker Tensor 27 FT-IR spectrophotometer was used to record IR spectra. Recording the NMR spectrum was handled in a Bruker Avance DRX-300 spectrometer with TMS as standard.

Mass spectra recorded on Finnigan-Matt 5973. Elemental analysis for C, H, N, and S determined using a Heracus CHN-O-Rapid analyzer. Microwave irradiations were carried in a MicroSynth, Milestone microwave oven with 2500 W power.

### 2.1. Synthesis of 4-Amino-5-alkyl-2,4-dihydro-[1,2,4]triazole-3-thione ( 4a–f )

A mixture consisting of carboxylic acid (0.01 mol) and thiocarbohydrazide (0.015 mol) was made in a round-bottomed flask heated on a mantle until content melted. The resulting mixture was washed several times with warm water to remove unreacted thiocarbohydrazide and carboxylic acid and then collected by filtration. To produce prementioned compounds, the product was recrystallized using ethanol.

#### 2.1.1. 4-amino-5-heptyl-2,4-dihydro-3H-1,2,4-triazole-3-thione (4a)

Yield 80%; white solid; m.p. 115–117 °C; IR υ (cm^−1^): 3320, 3200, 3152 (NH strength vibration of NH and NH_2_ groups), 2949-2851 (strength vibration of SP_3_ CH), 1486 (bending vibration of CH_2_); ^1^H NMR (300 MHz DMSO-d6) δ 0.84 (t, 3H, CH_3_), 1.23–1.26 (m, 8H, CH_2_), 1.56–1.63 (m, 2H, CH_2_), 2.59 (t, 2H, CH_2_), 5.49 (s, 2H, NH_2_), 13.40 (s, 1H, NH). Anal. Calcd. for C_9_H_18_N_4_S: C 50.44, H 8.47, N 26.14, S 14.95. found C 50.41, H 8.49, N 26.04, S 15.06.

#### 2.1.2. 4-amino-5-nonyl-2,4-dihydro-3H-1,2,4-triazole-3-thione (4b)

Yield 79%; white solid; m.p. 114–115 °C; IR υ (cm^−1^): 3320, 3200, 3152 (NH strength vibration of NH and NH_2_ groups), 2941-2851 (strength vibration of SP_3_ CH), 1486 (bending vibration of CH_2_); ^1^H NMR (300 MHz DMSO-d6) δ 0.84 (t, 3H, CH_3_), 1.23–1.25 (m, 12H, CH_2_), 1.58–1.64 (m, 2H, CH_2_), 2.59 (t, 2H, CH_2_), 5.50 (s, 2H, NH_2_), 13.40 (s, 1H, NH). Anal. Calcd. for C_11_H_22_N_4_S: C 54.51, H 9.15, N 23.12, S 13.22. found C 54.50, H 9.11, N 23.16, S 13.23.

#### 2.1.3. 4-amino-5-undecyl-2,4-dihydro-3H-1,2,4-triazole-3-thione (4c)

Yield 78%; white solid; m.p. 112–113.5 °C; IR υ (cm^−1^): 3320, 3248, 3138 (NH strength vibration of NH and NH_2_ groups), 2933-2851 (strength vibration of SP_3_ CH), 1486 (bending vibration of CH_2_); ^1^H NMR (300 MHz DMSO-d6) δ 0.83 (t, 3H, CH_3_), 1.23–1.26 (m, 16H, CH_2_), 1.58–1.63 (m, 2H, CH_2_), 2.59 (t, 2H, CH_2_), 5.49 (s, 2H, NH_2_), 13.40 (s, 1H, NH). Anal. Calcd. for C_13_H_26_N_4_S: C 57.74, H 9.69, N 20.72, S 11.85. found C 57.80, H 9.65, N 20.75, S 11.80.

#### 2.1.4. 4-amino-5-tridecyl-2,4-dihydro-3H-1,2,4-triazole-3-thione (4d)

Yield 75%; white solid; m.p. 110–112.5 °C; IR υ (cm^−1^): 3320, 3252, 3142 (NH strength vibration of NH and NH_2_ groups), 2927-2851 (strength vibration of SP_3_ CH), 1486 (bending vibration of CH2); 1H NMR (300 MHz DMSO-d6) δ 0.84 (t, 3H, CH3), 1.22–1.26 (m, 20H, CH2), 1.58–1.60 (m, 2H, CH2), 2.59 (t, 2H, CH2), 5.49 (s, 2H, NH2), 13.40 (s, 1H, NH). Anal. Calcd. for C15H30N4S: C 60.36, H 10.13, N 18.77, S 10.74. found C 60.26, H 10.20, N 18.80, S 10.74.

#### 2.1.5. 4-amino-5-pentadecyl-2,4-dihydro-3H-1,2,4-triazole-3-thione (4e)

Yield 71%; white solid; m.p. 108–110 °C; IR υ (cm^−1^): 3320, 3153, 3043 (NH strength vibration of NH and NH_2_ groups), 2920-2851 (strength vibration of SP_3_ CH), 1486 (bending vibration of CH_2_); ^1^H NMR (300 MHz DMSO-d6) δ 0.83 (t, 3H, CH_3_), 1.22–1.26 (m, 24H, CH_2_), 1.58–1.60 (m, 2H, CH_2_), 2.59 (t, 2H, CH_2_), 5.48 (s, 2H, NH_2_), 13.40 (s, 1H, NH). Anal. Calcd. for C_17_H_34_N_4_S: C 62.53, H 10.50, N 17.16, S 9.82. found C 62.49, H 10.41, N 17.18, S 9.92.

#### 2.1.6. 4-amino-5-heptadecyl-2,4-dihydro-3H-1,2,4-triazole-3-thione (4f)

Yield 70%; white solid; m.p. 106–108 °C; IR υ (cm^−1^): 3282, 3200, 3138 (NH strength vibration of NH and NH_2_ groups), 2922-2850 (strength vibration of SP_3_ CH), 1488 (bending vibration of CH_2_); ^1^H NMR (300 MHz DMSO-d6) δ 0.83 (t, 3H, CH_3_), 1.21–1.25 (m, 28H, CH_2_), 1.55–1.62 (m, 2H, CH_2_), 2.58 (t, 2H, CH_2_), 5.48 (s, 2H, NH_2_), 13.39 (s, 1H, NH). Anal. Calcd. for C_19_H_38_N_4_S: C 64.36, H 10.80, N 15.80, S 9.04. found C 64.25, H 10.70, N 15.90, S 9.15.

### 2.2. Synthesis of 4-(arylideneamino)-5-substituted-2,4-dihydro-1,2,4-triazole-3-thione (5a–l)

#### 2.2.1. Conventional procedure

An equimolar amount of corresponding substituted benzaldehyde with 3 to 4 drops of glacial acetic acid was added to a suspension of substituted amino triazole (1.2 mol) in methanol. The reaction mixture vessel was refluxed at temperature of 80–90°C for duration of 2 to 3 hours. Obtained precipitate was then washed with water, subsequently filtered and finally dried.

#### 2.2.2. Microwave procedure

Substituted amino triazole (1.2 mol) with an equimolar amount of the substituted benzaldehyde were mixed with 4–5 drops of DMSO and exposed to microwave irradiation at 80 °C (400 W) (see Table 1) using a Micro Synth lab station reactor. The reaction was carried within high-pressure Teflon reactor equipped with a magnetic stir bar and an optical fiber (to control resulting temperature).

Then, the mixture vessel was allowed to cool down while adding 20 mL water, and the obtained material was then filtered and washed using 10 mL of hot water and finally recrystallized in methanol.

#### 2.2.3. 4-(benzylideneamino)-5-heptyl-2,4-dihydro-3H-1,2,4-triazole-3-thione (5a)

Yield(%): 93(60); white solid; m.p. 119–121 °C; IR υ (cm^−1^): 3114 (NH strength vibration of NH group), 2950-2850 (strength vibration of SP_3_ CH), 1581, 1500 (strength vibration of C = C aromatic), 1469 (bending vibration of CH_2_); ^1^H NMR (300 MHz DMSO-d6) δ 0.82 (t, 3H, CH_3_), 1.26–1.42 (m, 8H, CH_2_), 1.74–1.80 (m, 2H, CH_2_), 2.84 (t, 2H, CH_2_), 7.46–7.87 (m, 5H, Ar), 10.35 (s, 1H, HC = N), 13.40 (s, 1H, NH); ^13^C NMR (75 MHz DMSO-d6) δ 14.2–31.8 (aliphatic, 7 carbons), 128.6–133.4 (aromatic, 6 carbons) 153.9, 157.2 (imine groups, 2 carbons), 181.3 (thione group, 1 carbon); M.S, m/z 302 (M^+^, 5%), 287 (M-NH, 3%), 258 (M-CS, 10%), 243 (M-NH-CS, 12%). Anal. Calcd. for C_16_H_22_N_4_S: C 63.54, H 7.33, N 18.53, S 10.60. found C 63.60, H 7.30, N 18.55, S 10.55.

#### 2.2.4. 4-(benzylideneamino)-5-nonyl-2,4-dihydro-3H-1,2,4-triazole-3-thione (5b)

Yield(%): 93(60); white solid; m.p. 116–118 °C; IR υ (cm^−1^): 3114 (strength vibration of NH group), 2950-2850 (strength vibration of SP_3_ CH), 1581, 1500 (strength vibration of C = C aromatic), 1468 (bending vibration of CH_2_); ^1^H NMR (300 MHz DMSO-d6) δ 0.82 (t, 3H, CH_3_), 1.21–1.40 (m, 12H, CH_2_), 1.70–1.89 (m, 2H, CH_2_), 2.80 (t, 2H, CH_2_), 7.46–7.87 (m, 5H, Ar), 10.31 (s, 1H, HC=N), 13.40 (s, 1H, NH ); ^13^C NMR (75 MHz DMSO-d6) δ14.4–34.10 (aliphatic, 9 carbons), 128.6–133.4 (aromatic, 6 carbons), 153.9, 157.2 (imine, 2 carbons), 181.3 (thione, 1 carbon); M.S, m/z 330 (M^+^, 6%), 315 (M-NH, 4%), 286 (N-C-S, 11%), 271 (M-NH-CS, 13%). Anal. Calcd. for C_18_H_26_N_4_S: C 65.42, H 7.93, N 16.95, S 9.70. found C 65.55, H 7.86, N 16.84, S 9.75.

#### 2.2.5. 4-(benzylideneamino)-5-undecyl-2,4-dihydro-3H-1,2,4-triazole-3-thione (5c)

Yield(%): 92(59); white solid; m.p. 112–115 °C; IR υ (cm^−1^): 3112 (strength vibration of NH group), 2950-2850 (strength vibration of SP_3_ CH), 1581, 1500 (strength vibration of C = C aromatic), 1468 (bending vibration of CH_2_); ^1^H NMR (300 MHz DMSO-d6) δ 0.80 (t, 3H, CH_3_), 1.24–1.41 (m, 16H, CH_2_), 1.67–1.83 (m, 2H, CH_2_), 2.78 (t, 2H, CH_2_), 7.46–7.87 (m, 5H, Ar), 10.28 (s, 1H, HC = N), 13.39 (s, 1H, NH); ^13^C NMR (75 MHz DMSO-d6)δ14.3–34.0 (aliphatic, 11 carbons), 128.8–133.2 (aromatic, 6 carbons), 154.9, 157.1 (imine, 2 carbons), 181.6 (thione, 1 carbon); M.S, m/z 358 (M^+^, 4%), 343 (M-NH, 4%), 314 (N-C-S, 11%), 299 (M-NH-CS, 12%). Anal. Calcd. for C_20_H_30_N_4_S: C 67.00, H 8.43, N 15.63, S 8.94. found C 66.91, H 8.50, N 15.60, S 8.99.

#### 2.2.6. 4-(benzylideneamino)-5-tridecyl-2,4-dihydro-3H-1,2,4-triazole-3-thione (5d)

Yield(%): 92(58); white solid; m.p. 99–111.5 °C; IR υ (cm^−1^): 3112 (strength vibration of NH group), 2950-2850 (strength vibration of SP_3_ CH), 1581, 1500 (strength vibration of C = C aromatic), 1468 (bending vibration of CH_2_); ^1^H NMR (300 MHz DMSO-d6) δ 0.80 (t, 3H, CH_3_), 1.16–1.38 (m, 20H, CH_2_), 1.60–1.62 (m, 2H, CH_2_), 2.73 (t, 2H, CH_2_), 7.47–7.86 (m, 5H, Ar), 10.23 (s, 1H, HC=N), 13.40 (s, 1H, NH); ^13^C NMR (75 MHz DMSO-d6)δ14.1–35.1 (aliphatic, 13 carbons), 127.9–133.1 (aromatic, 6 carbons), 154.1, 158.1 (imine, 2 carbons), 181.2 (thione, 1 carbon); M.S, m/z 386 (M^+^, 5%), 371 (M-NH, 4%), 342 (N-C-S, 12%), 327 (M-NH-CS, 11%). Anal. Calcd. for C_22_H_34_N_4_S: C 68.36, H 8.86, N 14.49, S 8.29. found C 68.33, H 8.85, N 14.47, S 8.35.

#### 2.2.7. 4-(benzylideneamino)-5-pentadecyl-2,4-dihydro-3H-1,2,4-triazole-3-thione (5e)

Yield(%): 91(57); white solid; m.p. 96–98 °C; IR υ (cm^−1^): 3112 (NH strength vibration of NH group), 2950-2850 (strength vibration of SP_3_ CH), 1581, 1500 (strength vibration of C=C aromatic), 1468 (bending vibration of CH_2_); ^1^H NMR (300 MHz DMSO-d6) δ 0.80 (t, 3H, CH_3_), 1.15–1.23 (m, 24H, CH_2_), 1.57–1.64 (m, 2H, CH_2_), 2.68 (t, 2H, CH_2_), 7.47–7.86 (m, 5H, Ar), 10.23 (s, 1H, HC=N), 13.41 (s, 1H, NH); ^13^C NMR (75 MHz DMSO-d6)δ14.1–29.7 (aliphatic, 15 carbons), 127.9–133.1 (aromatic, 6 carbons), 154.1, 158.1 (imine, 2 carbons), 181.2 (thione, 1 carbon); M.S, m/z 414 (M^+^, 6%), 399 (M-NH, 6%), 370 (N-C-S, 13%), 355 (M-NH-CS, 14%). Anal. Calcd. for C_24_H_38_N_4_S: C 69.52, H 9.24, N 13.51, S 7.73. found C 69.38, H 9.17, N 13.63, S 7.82.

#### 2.2.8. 4-(benzylideneamino)-5-heptadecyl-2,4-dihydro-3H-1,2,4-triazole-3-thione (5f)

Yield(%): 90(57); white solid; m.p. 90–92 °C; IR υ (cm^−1^): 3115 (NH strength vibration of NH group), 2921-2851 (strength vibration of SP_3_ CH), 1583, 1499 (strength vibration of C=C aromatic), 1417 (bending vibration of CH_2_); ^1^H NMR (300 MHz DMSO-d6) δ 0.81 (t, 3H, CH_3_), 1.08–1.26 (m, 28H, CH_2_), 1.60–1.71 (m, 2H, CH_2_), 2.69 (t, 2H, CH_2_), 7.52–7.88 (m, 5H, Ar), 10.23 (s, 1H, HC = N), 13.40 (s, 1H, NH); ^13^C NMR (75 MHz DMSO-d6) δ13.9–34.2 (aliphatic, 17 carbons), 128.7–132.9 (aromatic, 6 carbons), 153.7, 157.2 (imine, 2 carbons), 182.1 (thione, 1 carbon); M.S, m/z 442 (M^+^, 7%), 427 (M-NH, 5%), 398 (N-C-S, 9%), 383 (M-NH-CS, 13%). Anal. Calcd. for C_26_H_42_N_4_S: C 70.54, H 9.56, N 12.66, S 7.24. found C 70.61, H 9.51 N 12.61, S 7.27.

#### 2.2.9. 5-heptyl-4-((2-hydroxybenzylidene)amino)-2,4-dihydro-3H-1,2,4-triazole-3-thione (5g)

Yield(%): 94(62); white solid; m.p. 125–127 °C; IR υ (cm^−1^): 3450-3200 (strength vibration of OH group), 3105 (strength vibration of NH group), 2917-2850 (strength vibration of SP_3_ CH), 1585, 1488 (strength vibration of C=C aromatic), 1465 (bending vibration of CH_2_); ^1^H NMR (300 MHz DMSO-d6) δ 0.82 (t, 3H, CH_3_), 1.26–1.42 (m, 8H, CH_2_), 1.56–1.71 (m, 2H, CH_2_), 2.73 (t, 2H, CH_2_), 6.80–7.33 (m, 4H, Ar), 10.32 (s, 1H, HC=N), 10.89 (s, 1H, OH), 13.40 (s, 1H, NH); ^13^C NMR (75 MHz DMSO-d6) δ13.8–31.9 (aliphatic, 7 carbons), 117.7–132.3, 156.7 (aromatic, 6 carbons), 143.4, 156.0 (imine, 2 carbons), 181.2 (thione, 1 carbon); M.S, m/z 318 (M^+^, 6%), 303 (M-NH, 5%), 274 (N-C-S, 10%), 259 (M-NH-CS, 11%). Anal. Calcd. for C_16_H_22_N_4_OS: C 60.35, H 6.96, N 17.59, O 5.03, S 10.07. found C 60.50, H 6.99, N 17.51, O 4.99 S 10.0.1

#### 2.2.10. 4-((2-hydroxybenzylidene)amino)-5-nonyl-2,4-dihydro-3H-1,2,4-triazole-3-thione (5h)

Yield(%): 93(61); white solid; m.p. 121–124 °C; IR υ (cm^−1^): 3430-3190 (strength vibration of OH group), 3150 (strength vibration of NH group), 2930-2860 (strength vibration of SP_3_ CH), 1590, 1495 (strength vibration of C = C aromatic), 1475 (bending vibration of CH_2_); ^1^H NMR (300 MHz DMSO-d6) δ 0.83 (t, 3H, CH_3_), 1.20–1.25 (m, 12H, CH_2_), 1.60–1.65 (m, 2H, CH_2_), 2.65 (t, 2H, CH_2_), 6.83–7.45 (m, 4H, Ar), 10.51 (s, 1H, HC = N), 10.89 (s, 1H, OH), 13.40 (s, 1H, NH); ^13^C NMR (75 MHz DMSO-d6) δ14.3–34.2 (aliphatic, 9 carbons), 117.5–132.1, 157.2 (aromatic, 6 carbons), 143.1, 156.3 (imine, 2 carbons), 181.5 (thione, 1 carbon); M.S, m/z 346 (M^+^, 5%), 331 (M-NH, 4%), 302 (N-C-S, 12%), 287 (M-NH-CS, 14%). Anal. Calcd. for C_18_H_26_N_4_OS: C 62.40, H 7.56, N 16.17, O 4.62, S 9.25. found C 62.30, H 7.53, N 16.21, O 4.66, S 9.30

#### 2.2.11. 4-((2-hydroxybenzylidene)amino)-5-undecyl-2,4-dihydro-3H-1,2,4-triazole-3-thione (5i)

Yield(%): 93(61); white solid; m.p. 116–118 °C; IR υ (cm^−1^): 3500-3250 (strength vibration of OH group), 3180 (strength vibration of NH group), 2950-2840 (strength vibration of SP_3_ CH), 1595, 1500 (strength vibration of C=C aromatic), 1470 (bending vibration of CH_2_); ^1^H NMR (300 MHz DMSO-d6) δ 0.82 (t, 3H, CH_3_), 1.26–1.42 (m, 16H, CH_2_), 1.47–1.80 (m, 2H, CH_2_), 2.49 (t, 2H, CH_2_), 6.79–7.45 (m, 4H, Ar), 10.50 (s, 1H, HC = N), 10.89 (s, 1H, OH), 13.41 (s, 1H, NH); ^13^C NMR (75 MHz DMSO-d6) δ14.4–34.1 (aliphatic, 11 carbons), 117.5–133.1, 157.4 (aromatic, 6 carbons), 144.8, 156.9 (imine, 2 carbons), 181.4 (thione, 1 carbon); M.S, m/z 374 (M^+^, 5%), 359 (M-NH, 5%), 330 (N-C-S, 12%), 315 (M-NH-CS, 11%). Anal. Calcd. for C_20_H_30_N_4_OS: C 64.14, H 8.07, N 14.96, O 4.27, S 8.56. found C 64.25, H 8.05, N 14.86, O 4.31, S 8.53

#### 2.2.12. 4-((2-hydroxybenzylidene)amino)-5-tridecyl-2,4-dihydro-3H-1,2,4-triazole-3-thione (5j)

Yield(%): 92(59); white solid; m.p. 112–115 °C; IR υ (cm^−1^): 3480-3210 (strength vibration of OH group) ), 3145 (strength vibration of NH group), 2945-2833 (strength vibration of SP_3_ CH), 1600, 1490 (strength vibration of C = C aromatic), 1466 (bending vibration of CH_2_); ^1^H NMR (300 MHz DMSO-d6) δ 0.81 (t, 3H, CH_3_), 1.06–1.18 (m, 20H, CH_2_), 1.56–1.63 (m, 2H, CH_2_), 2.67 (t, 2H, CH_2_), 6.82–7.44 (m, 4H, Ar), 10.49 (s, 1H, HC = N), 10.89 (s, 1H, OH), 13.39 (s, 1H, NH); ^13^C NMR (75 MHz DMSO-d6) δ14.5–34.9 (aliphatic, 13 carbons), 117.3–131.9, 157.7 (aromatic, 6 carbons), 143.6, 157.1 (imine, 2 carbons), 181.3 (thione, 1 carbon); M.S, m/z 402 (M^+^, 6%), 387 (M-NH, 4%), 358 (N-C-S, 12%), 343 (M-NH-CS, 12%). Anal. Calcd. for C_22_H_34_N_4_OS: C 65.63, H 8.51, N 13.92, O 3.97 S 7.97. found C 65.75, H 8.47, N 13.85, O 3.95, S 7.98.

#### 2.2.13. 4-((2-hydroxybenzylidene)amino)-5-pentadecyl-2,4-dihydro-3H-1,2,4-triazole-3-thione (5k)

Yield(%): 91(59); white solid; m.p. 108–110 °C; IR υ (cm^−1^): 3500-3200 (strength vibration of OH group) ), 3105 (strength vibration of NH group), 2917-2850 (strength vibration of SP_3_ CH), 1597, 1509 (strength vibration of C = C aromatic), 1467 (bending vibration of CH_2_); ^1^H NMR (300 MHz DMSO-d6) δ 0.81 (t, 3H, CH_3_), 1.26–1.32 (m, 24H, CH_2_), 1.74–1.80 (m, 2H, CH_2_), 2.65 (t, 2H, CH_2_), 6.80–7.46 (m, 4H, Ar), 10.43 (s, 1H, HC=N), 10.84 (s, 1H, OH), 13.40 (s, 1H, NH); ^13^C NMR (75 MHz DMSO-d6) δ14.6–34.0 (aliphatic, 15 carbons), 117.8–132.3, 157.8 (aromatic, 6 carbons), 143.3, 157.1 (imine, 2 carbons), 182.1 (thione, 1 carbon); M.S, m/z 430 (M^+^, 7%), 415 (M-NH, 3%), 386 (N-C-S, 11%), 371 (M-NH-CS, 14%). Anal. Calcd. for C_24_H_38_N_4_OS: C 66.94, H 8.89, N 13.01, O 3.72 S 7.44. found C 67.01, H 8.94, N 12.96, O 3.69, S 7.40.

#### 2.2.14. 5-heptadecyl-4-((2-hydroxybenzylidene)amino)-2,4-dihydro-3H-1,2,4-triazole-3-thione (5l)

Yield(%): 91(58); white solid; m.p. 97–99 °C; IR υ (cm^−1^): 3480-3330 (strength vibration of OH group) ), 3105 (strength vibration of NH group), 2918-2840 (strength vibration of SP_3_ CH), 1593, 1505 (strength vibration of C = C aromatic), 1466 (bending vibration of CH_2_); ^1^H NMR (300 MHz DMSO-d6) δ 0.80 (t, 3H, CH_3_), 1.16–1.22 (m, 28H, CH_2_), 1.57–1.64 (m, 2H, CH_2_), 2.68 (t, 2H, CH_2_), 6.85–7.46 (m, 4H, Ar), 10.44 (s, 1H, HC = N), 10.88 (s, 1H, OH), 13.41 (s, 1H, NH); ^13^C NMR (75 MHz DMSO-d6) δ14.9–34.2 (aliphatic, 17 carbons), 118.1–131.9, 157.1 (aromatic, 6 carbons), 143.4, 156.9 (imine, 2 carbons), 182.4 (thione, 1 carbon); M.S, m/z 458 (M^+^, 6%), 443 (M-NH, 5%), 414 (N-C-S, 11%), 399 (M-NH-CS, 11%). Anal. Calcd. for C_26_H_42_N_4_OS: C 68.08, H 9.22, N 12.21, O 3.50, S 6.99. found C 68.07, H 9.25, N 12.18, O 3.52, S 6.98.

## 3. Results and discussion

In a continuous research works of this group [27–34], 18 triazole amines and Schiff bases were synthesized. The difference among these compounds is in the R_1_ acid group and the R_2_ aldehyde group. Our research team performed the reaction of thiocarbohydrazide (TCH) with long-chain aliphatic carboxylic acids (1a–f) in different conditions and analyzed the reaction of the resulting products with benzaldehyde and its derivatives under various conditions.

**Figure 1:**
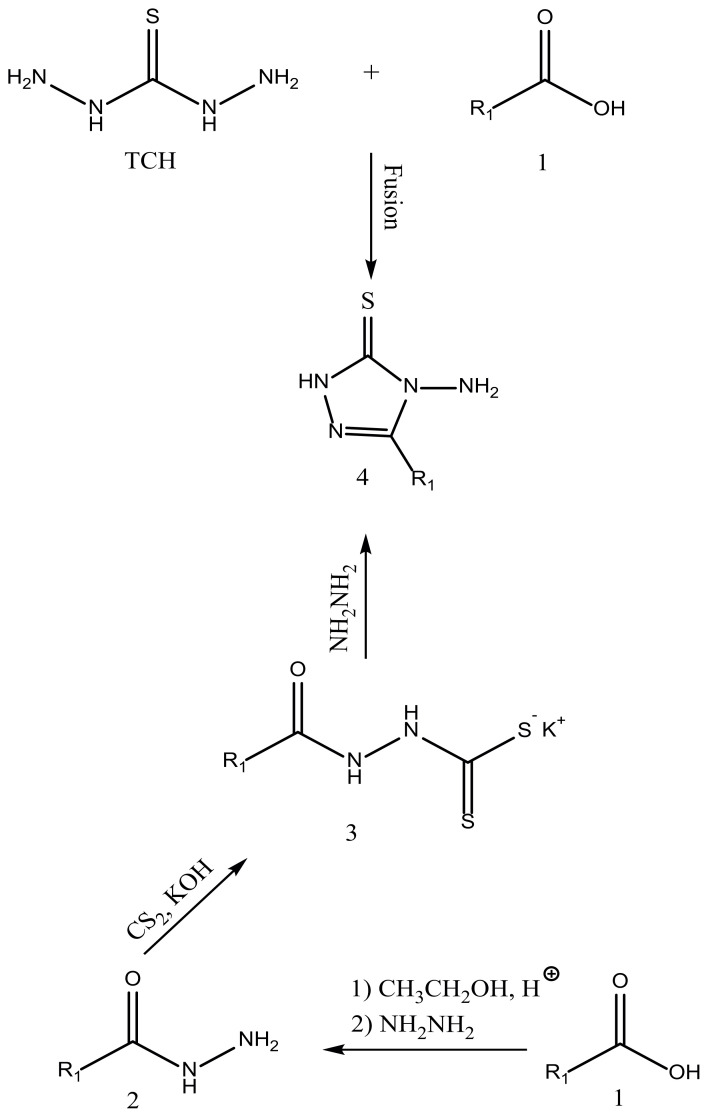
Synthesis of 1,2,4-triazole amines 4a–f.

**Figure 2:**
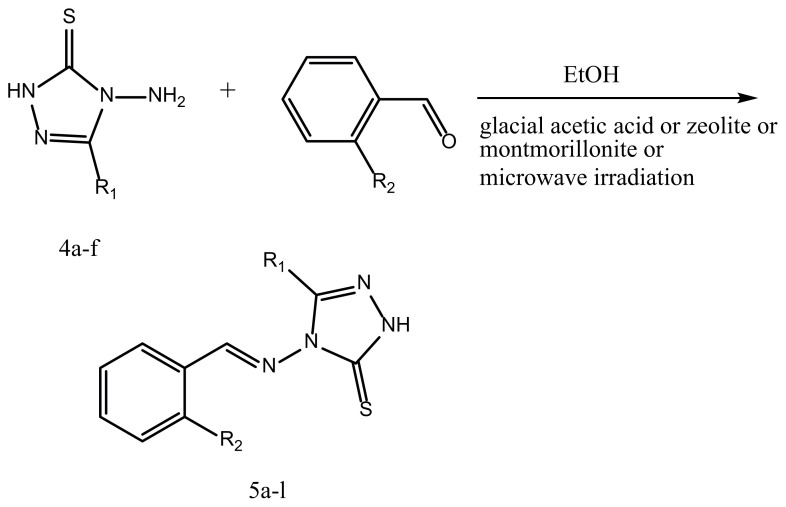
Synthesis of Schiff base 5a–l.

The reaction of TCH with octanoic acid 1a in the fusion method yielded product 4a (Figure 1), and it was confirmed as 4-amino-5-heptyl-2,4-dihydro-3H-1,2,4-triazole-3-thione (4a). The structure of 4a was assessed and approved as compared to its spectral data (^1^H NMR, IR). Also, triazole 4a was synthesized in three steps reaction as well. Hydrazide (2) was obtained by the ethanolysis and then hydrazinolysis of carboxylic acid (1). The required dithiocarbazinate (3) was synthesized by reacting hydrazide with carbon disulfide and ethanolic solution of potassium hydroxide. And 1,2,4-triazole(4) was produced by the reaction of intramolecular cyclization from the yielded salt with hydrazine. The advantage of the first method is the lower steps and higher efficiency. The triazole synthesized in this way was then condensed with benzaldehyde. As a catalyst, the reaction was performed in the presence of concentrated sulfuric acid or glacial acetic acid (few drops) to yield Schiff base 5 (Figure 2). The Schiff base formation reaction was carried out by the use of bronsted acid. The reaction was also accomplished in the presence of heterogeneous inorganic solid acidic catalysts such as beta-zeolite and montmorillonite-KSF under heat conditions. And also, the reaction was conducted in the absence of catalyst in microwave environment. The efficiency obtained using zeolite and montmorillonite was approximately similar. Besides, when the reaction was carried out under microwave conditions, the reaction time was reduced and the efficiency increased. The obtained yield using catalyst and in conventional method was moderate. Reactions were evaluated at different times. The best results were obtained with acetic acid in the thermal method for 90 min and in the microwave method for 12 min (see Table 2).

The path for synthesizing proposed compounds 4a–f and 5a–l is outlined in Figures 1 and 2. 4a–f was approved by IR and ^1^H NMR spectroscopic methods. 5a–f was also fully characterized using various spectroscopic methods. The progress for reactions was assessed using TLC (thin layer chromatography). Elemental analysis was used to check the purity of all compounds.

The position of IR bands suggests enough evidence regarding the formation of 4a and 5a. The bands due to υ ( C = N ) and υ (C = S) stretch at 1616 cm^−1^ and 1223 cm^−1^, which approves the formation of triazole 4a. The absence of υ (NH_2_) band in the IR spectrum of 4a shows the formation of 5a.

Other signs for the formation of 4a and 5a were attained by ^1^H NMR and ^13^C NMR spectroscopies. In the ^1^H NMR of triazole 4a in d_6_-DMSO, signals for the NH proton of triazole ring and for the NH_2_ protons were observed at 13.40 ppm and 5.49 ppm, respectively. In the ^1^H NMR of Schiff base 5a in d_6_-DMSO, signal for the CH proton of imine bond was observed at 10.35 ppm, beside the ^13^C NMR spectrum signal at 154.1 or 157.0 ppm due to -CH=N- carbon atom. Also, in the mass spectrum of 5a, fragment 302 obtained. These results support for the proposed structures of 4a and 5a.

## 4. Conclusion

In this work, Schiff base 5 was prepared in two steps. In the first stage, amine triazole 4 was prepared from the reaction of thiocarbohydrazide and carboxylic acid, and in the second stage, compound 4 reacted with benzaldehyde and its derivatives and the final product 5 was obtained. The second step reaction was performed using sulfuric or acetic acid, zeolite, montmorillonite, and microwave irradiations. The best efficiency was achieved in the microwave.

























































































































## Figures and Tables

**Table 1:** Chemical structures of 1,2,4-triazole 4a–f and Schiff base 5a–l.

Entry	Compounds	R1	R2	Yield (%)	
				Conventional heating	Microwave heating (12 min.)
1	4a	n-C_7_H_15_	---	80	---
2	4b	n-C_9_H_19_	---	79	---
3	4c	n-C_11_H_23_	---	78	---
4	4d	n-C_13_H_27_	---	75	---
5	4e	n-C_15_H_31_	---	71	---
6	4f	n-C_17_H_35_	---	70	---
7	5a	n-C_7_H_15_	H	60	93
8	5b	n-C_9_H_19_	H	60	93
9	5c	n-C_11_H_23_	H	59	92
10	5d	n-C_13_H_27_	H	58	92
11	5e	n-C_15_H_31_	H	57	91
12	5f	n-C_17_H_35_	H	57	90
13	5g	n-C_7_H_15_	OH	62	94
14	5h	n-C_9_H_19_	OH	61	93
15	5i	n-C_11_H_23_	OH	61	93
16	5j	n-C_13_H_27_	OH	59	92
17	5k	n-C_15_H_31_	OH	59	91
18	5l	n-C_17_H_35_	OH	58	91

**Table 2:** The effect of catalyst on the reaction efficiency of Schiff base 5a*.

Time (min.)	Yield (%)			
	Conventional heating			Microwave heating
	Acetic acid	β-Zeolite	Montmorillonite KSF	
5	---	---	---	90
8	---	---	---	91
10	---	---	---	92
12	---	---	---	93
30	53	51	54	---
45	55	51	55	---
60	56	52	56	---
90	60	52	57	---

* The effect of the catalyst was investigated only on 5a.

The reaction efficiency using microwaves did not change significantly with increasing time. The start of the reaction in the presence of the catalyst was after 30 min.
