# Supplementary figures and images for: Microwave irradiated synthesis of Schiff bases of 4-(arylideneamino)-5-alkyl-2,4-dihydro-1,2,4-triazole-3-thione containing 1,2,4-triazole segment
Source: Turk J Chem. 2021 Jul 18;45(6):1805–13. doi: 10.3906/kim-2105-39 (PMC10734766; doi:10.3906/kim-2105-39)

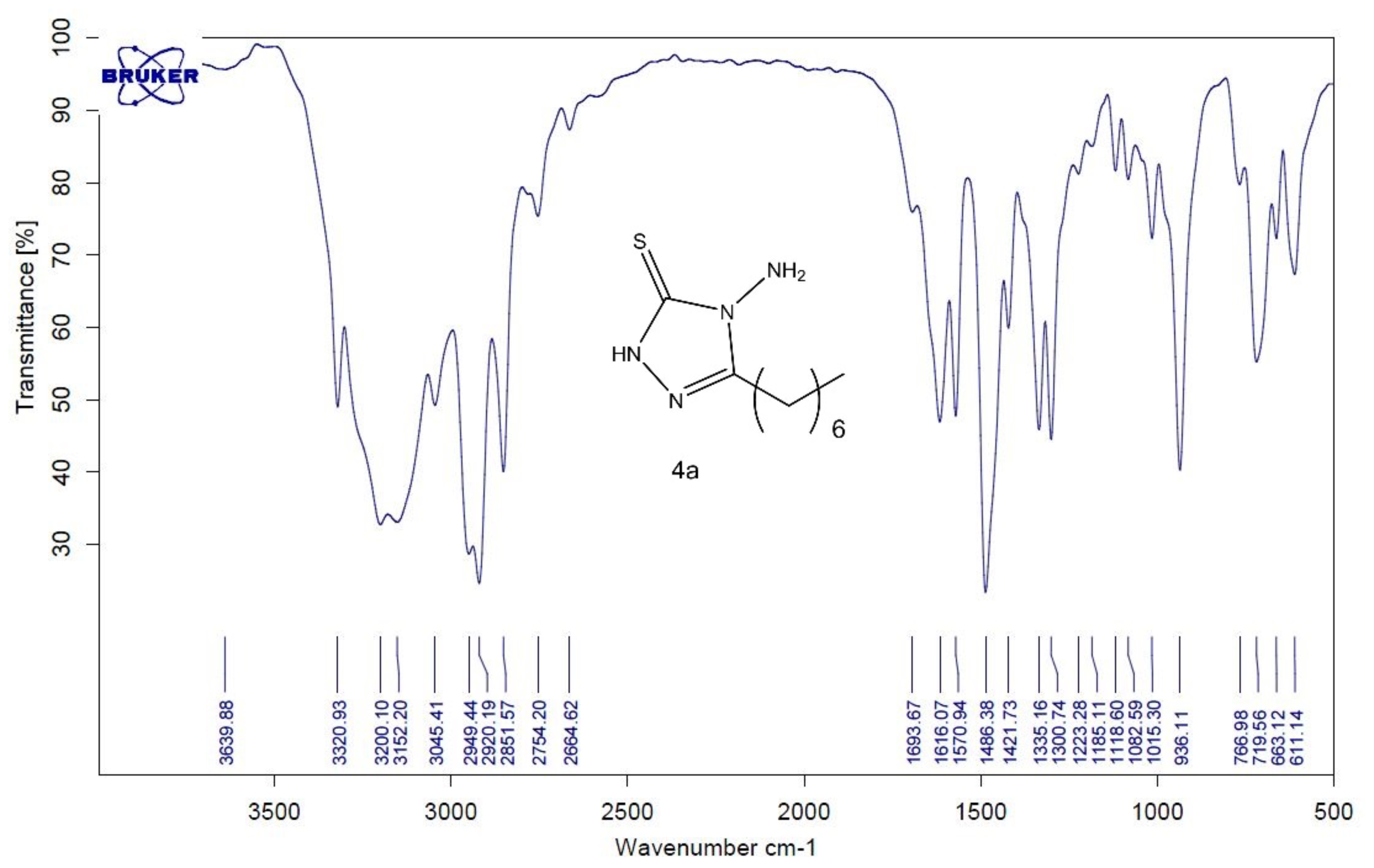

Supplement: Supplementary file 1 [file turkjchem-45-6-1805s1.tif]

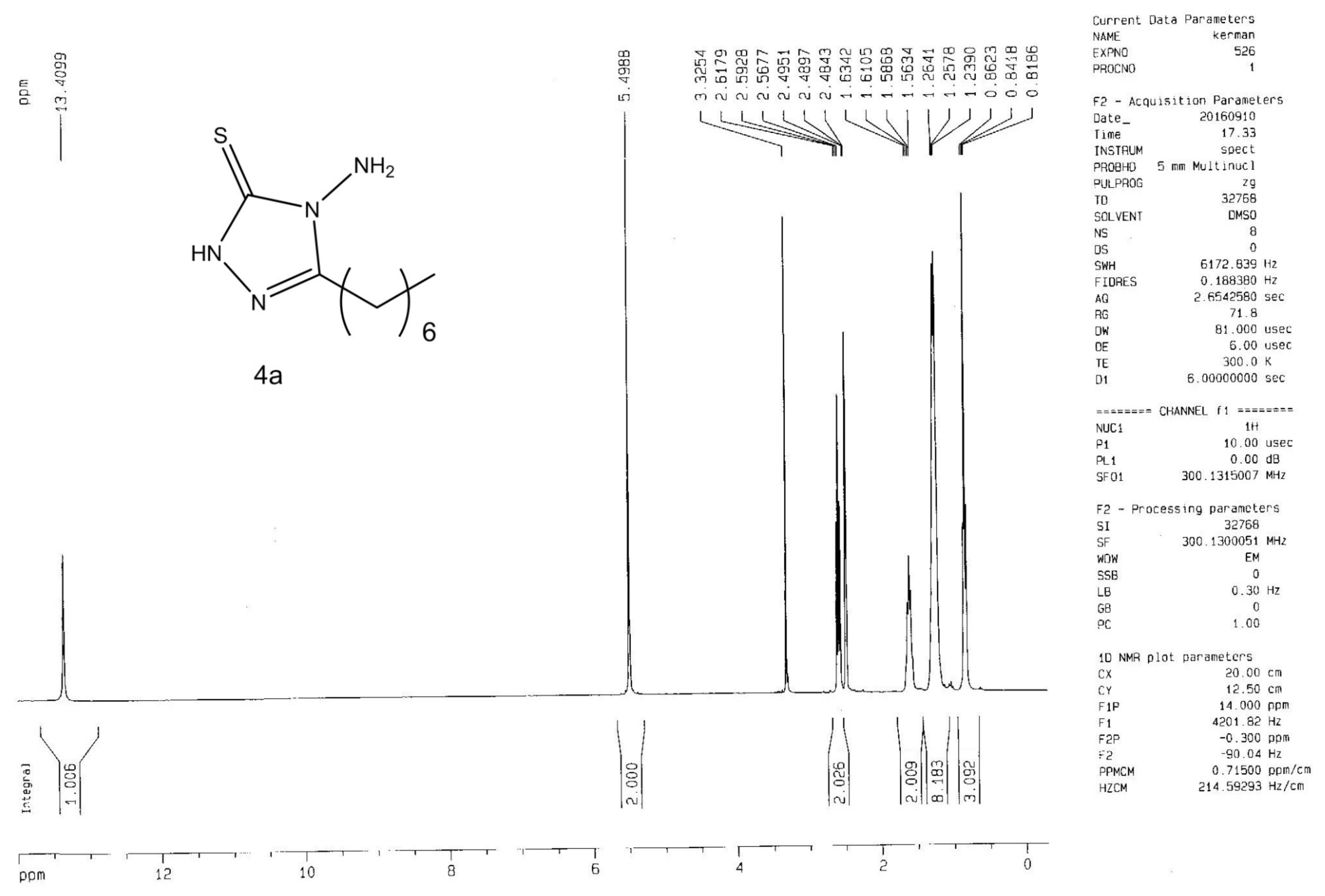

Supplement: Supplementary file 2 [file turkjchem-45-6-1805s2.tif]

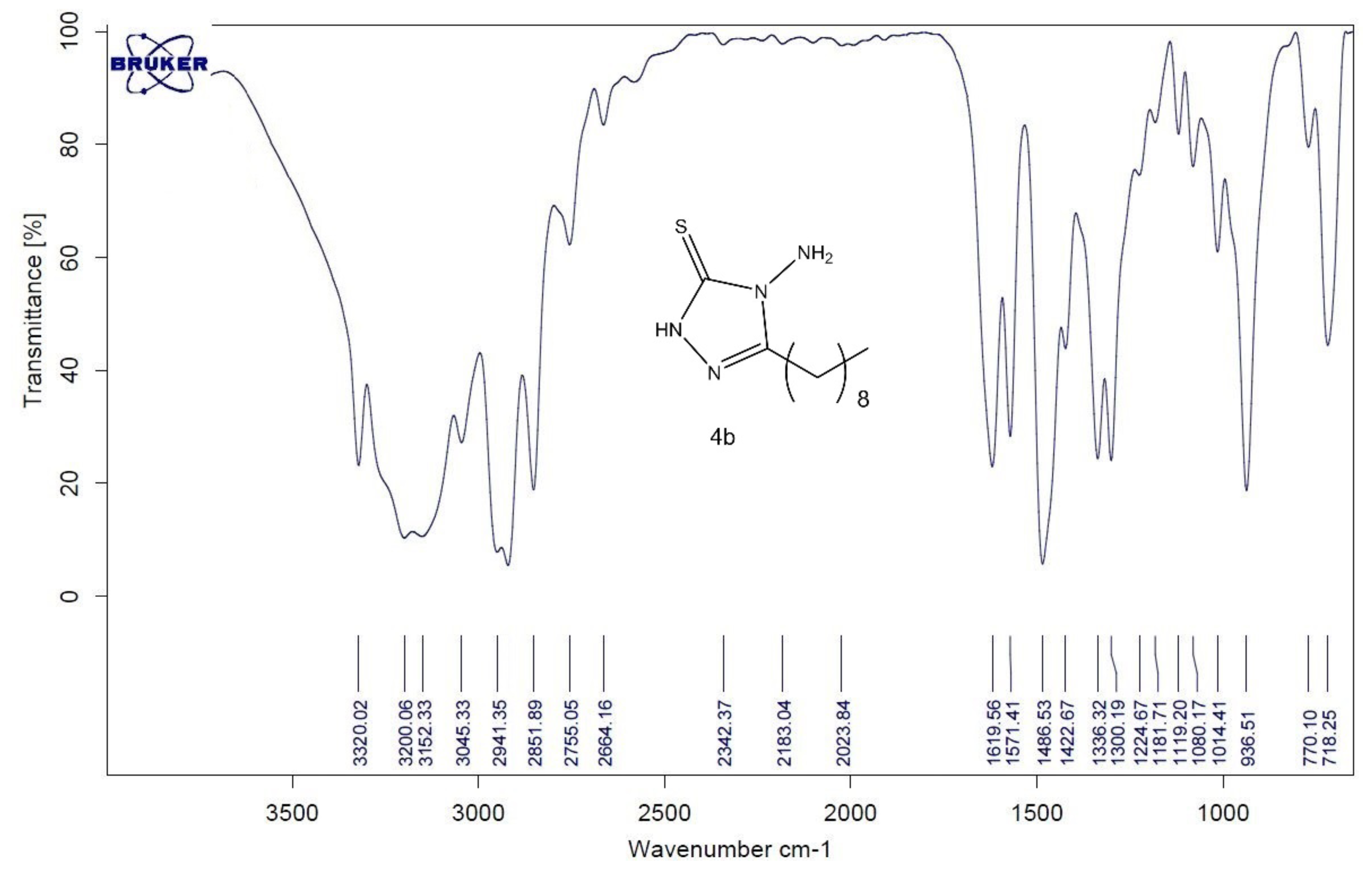

Supplement: Supplementary file 3 [file turkjchem-45-6-1805s3.tif]

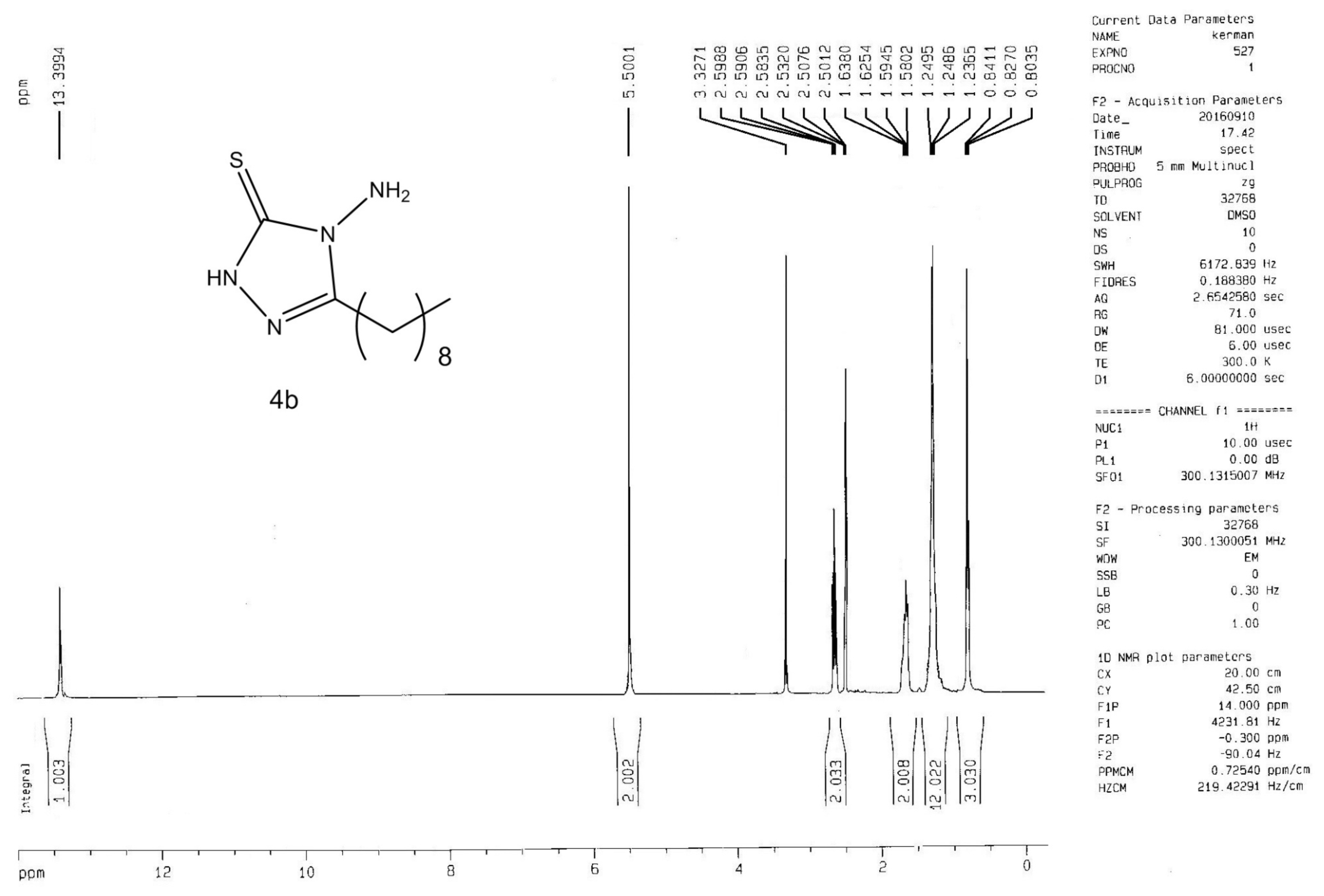

Supplement: Supplementary file 4 [file turkjchem-45-6-1805s4.tif]

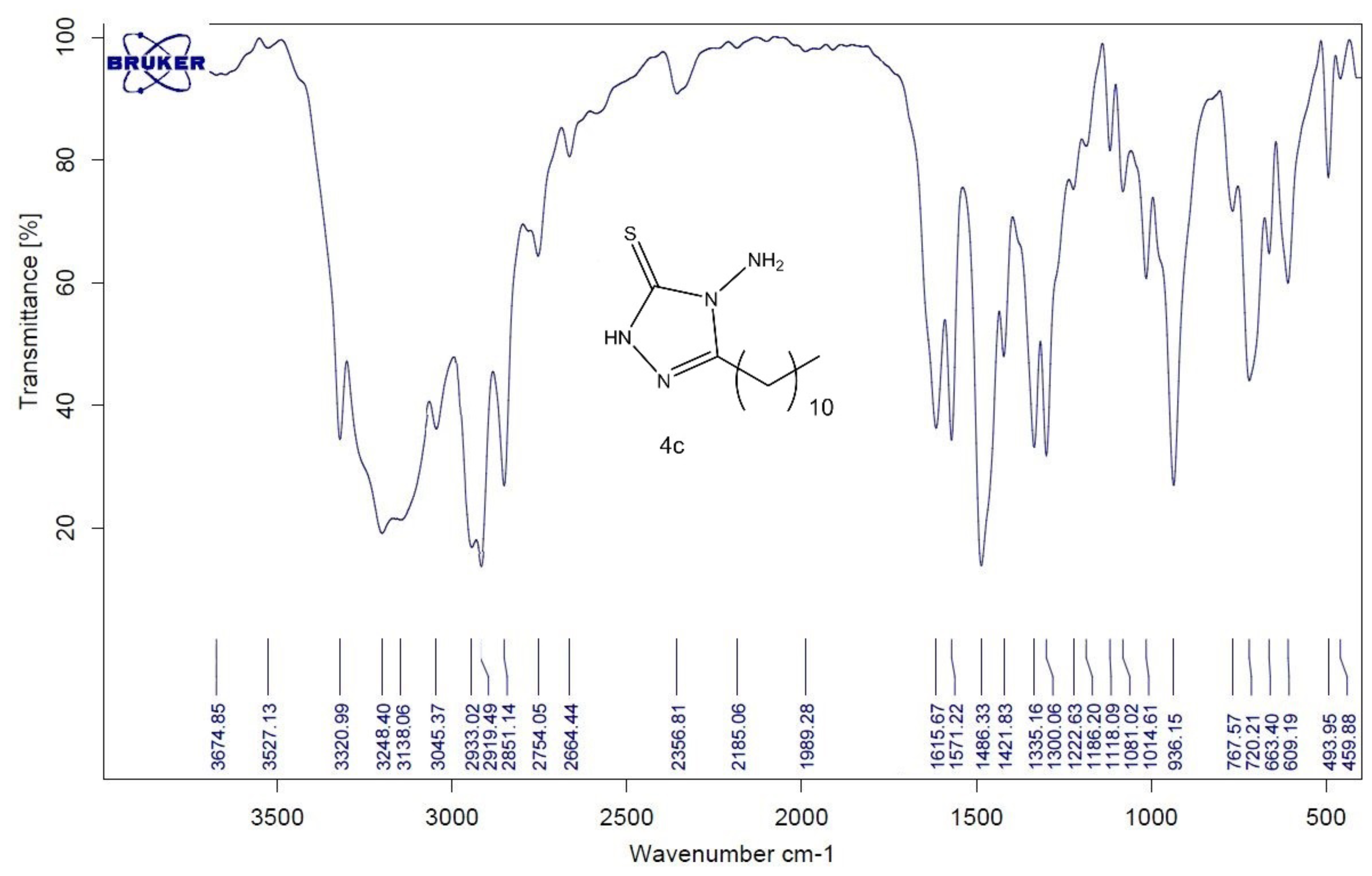

Supplement: Supplementary file 5 [file turkjchem-45-6-1805s5.tif]

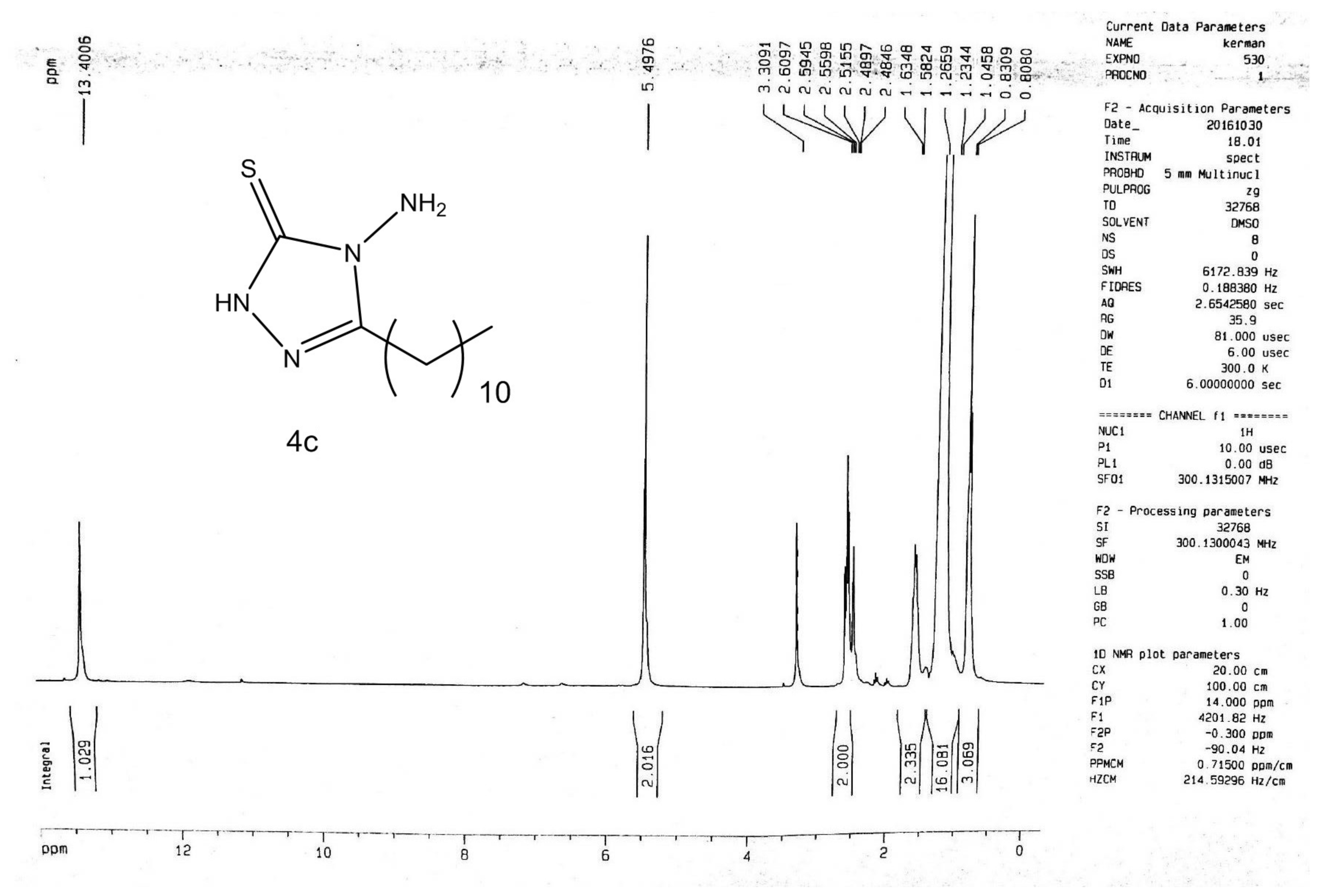

Supplement: Supplementary file 6 [file turkjchem-45-6-1805s6.tif]

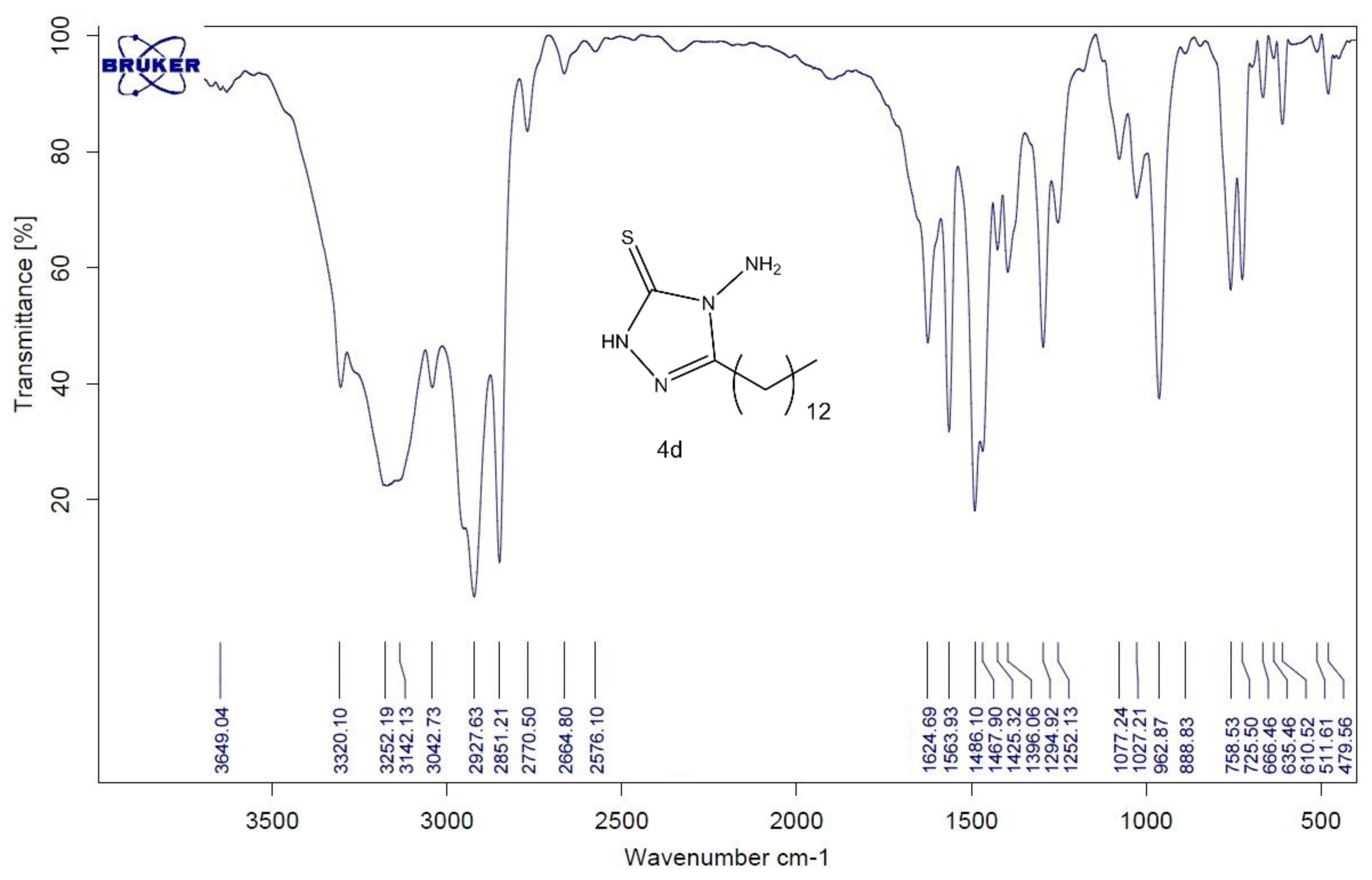

Supplement: Supplementary file 7 [file turkjchem-45-6-1805s7.tif]

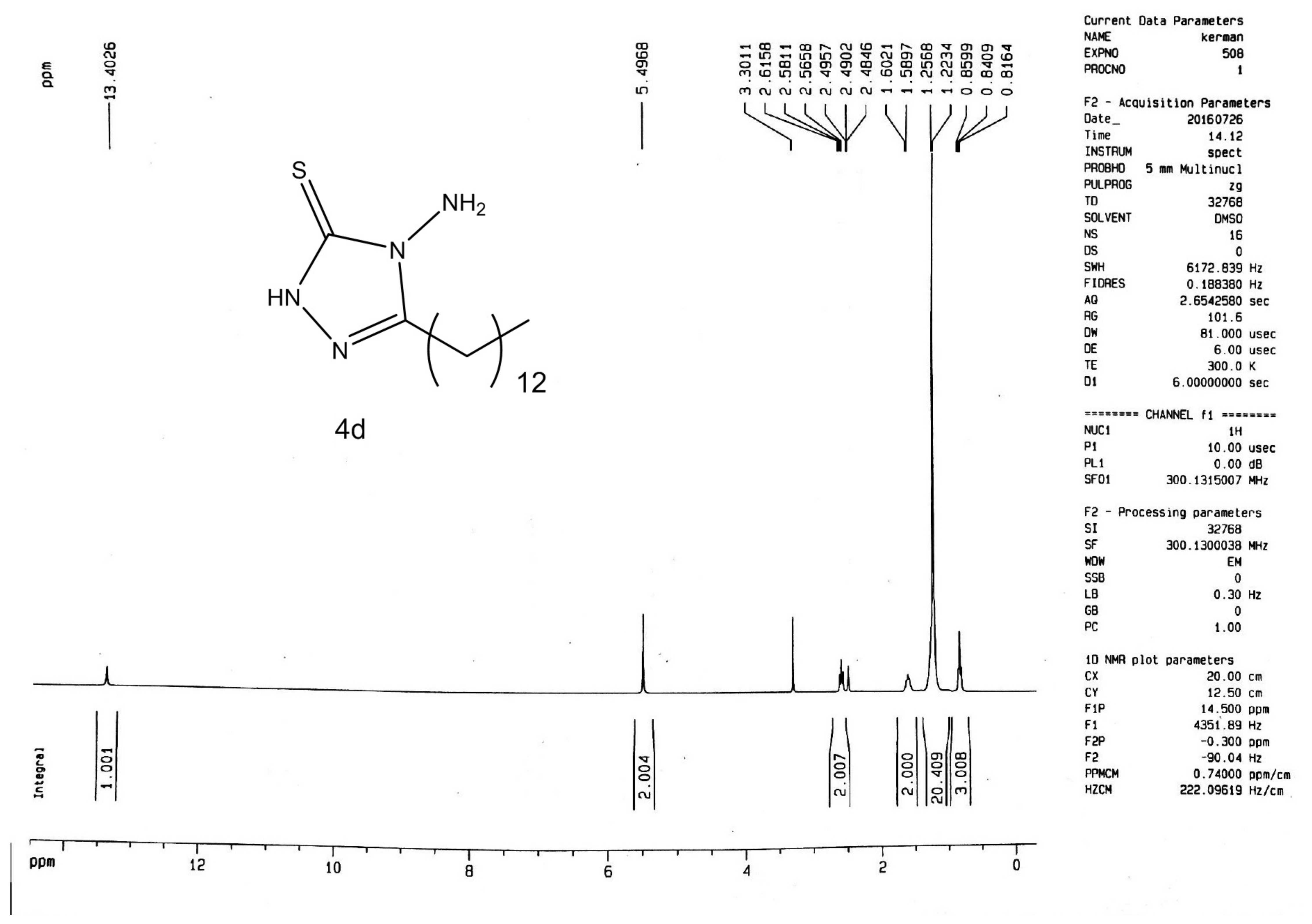

Supplement: Supplementary file 8 [file turkjchem-45-6-1805s8.tif]

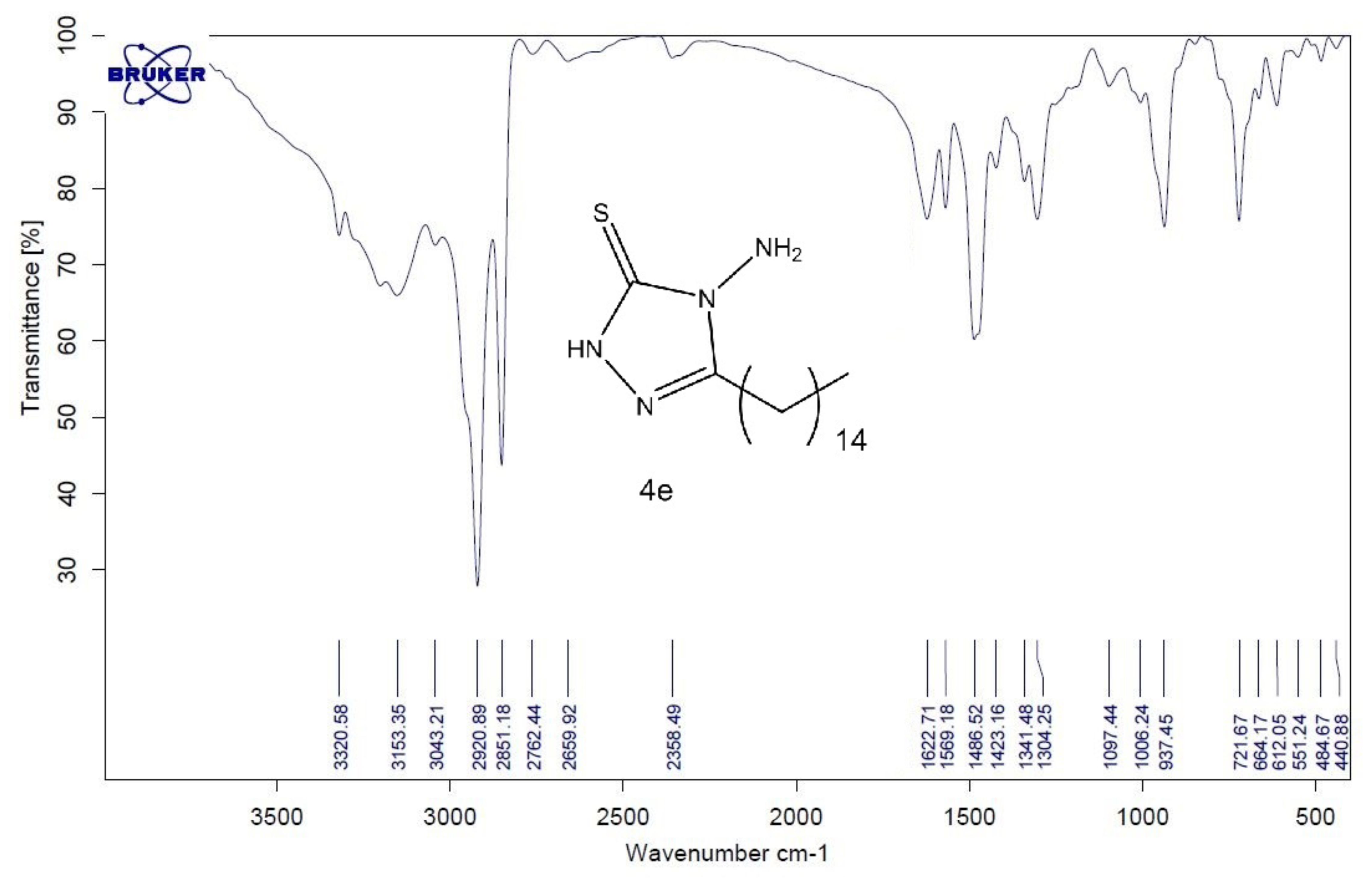

Supplement: Supplementary file 9 [file turkjchem-45-6-1805s9.tif]

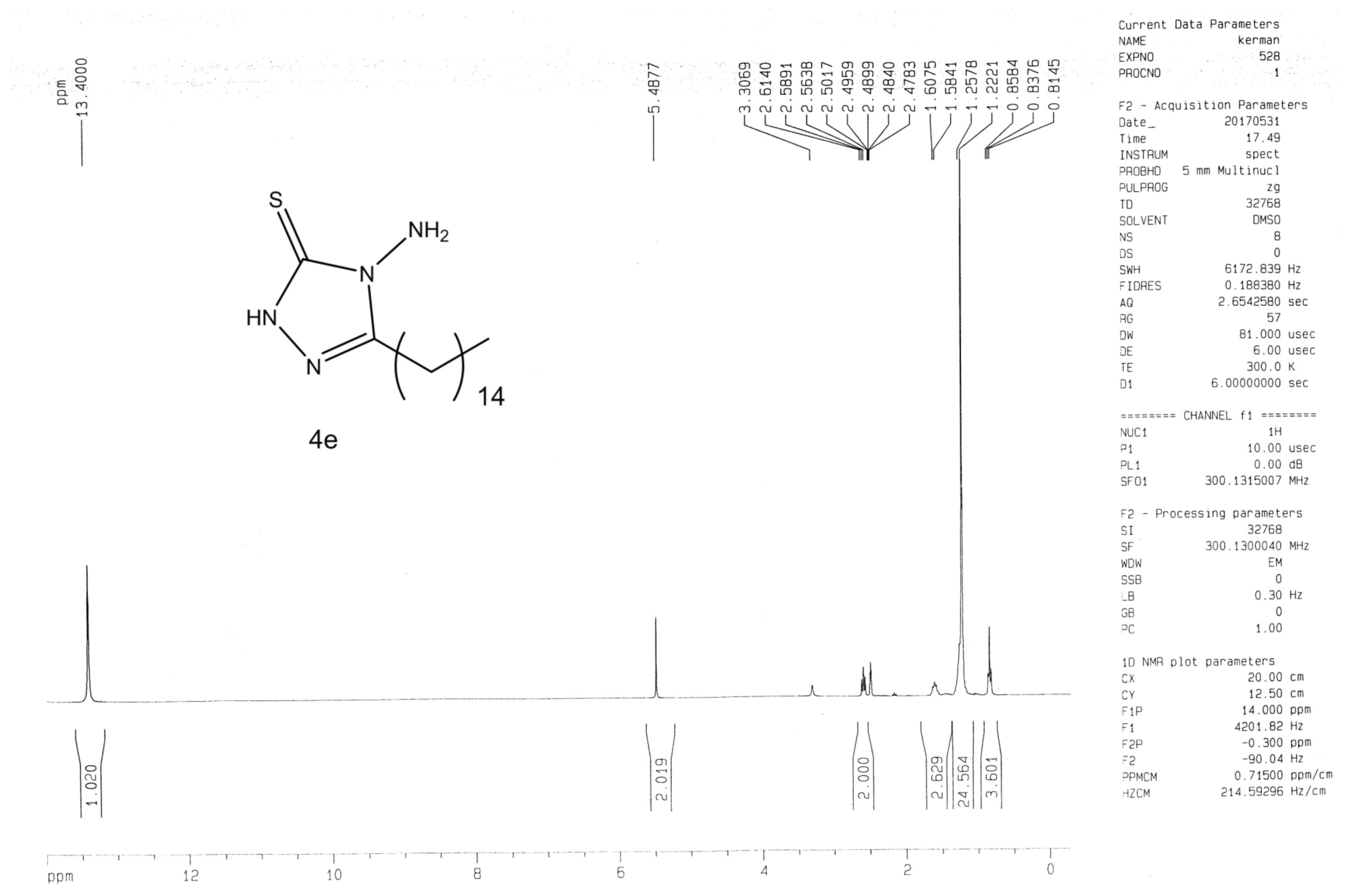

Supplement: Supplementary file 10 [file turkjchem-45-6-1805s10.tif]

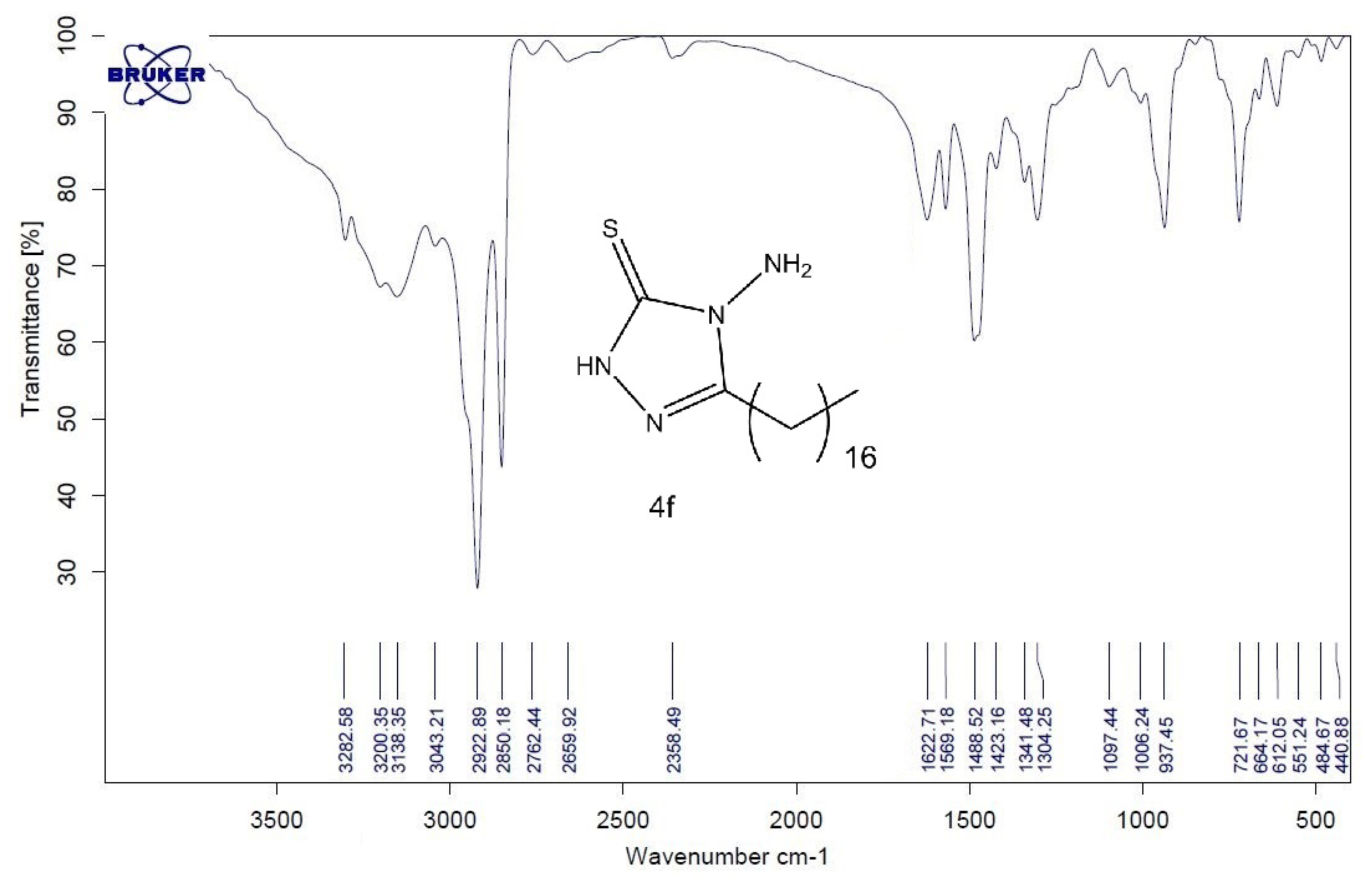

Supplement: Supplementary file 11 [file turkjchem-45-6-1805s11.tif]

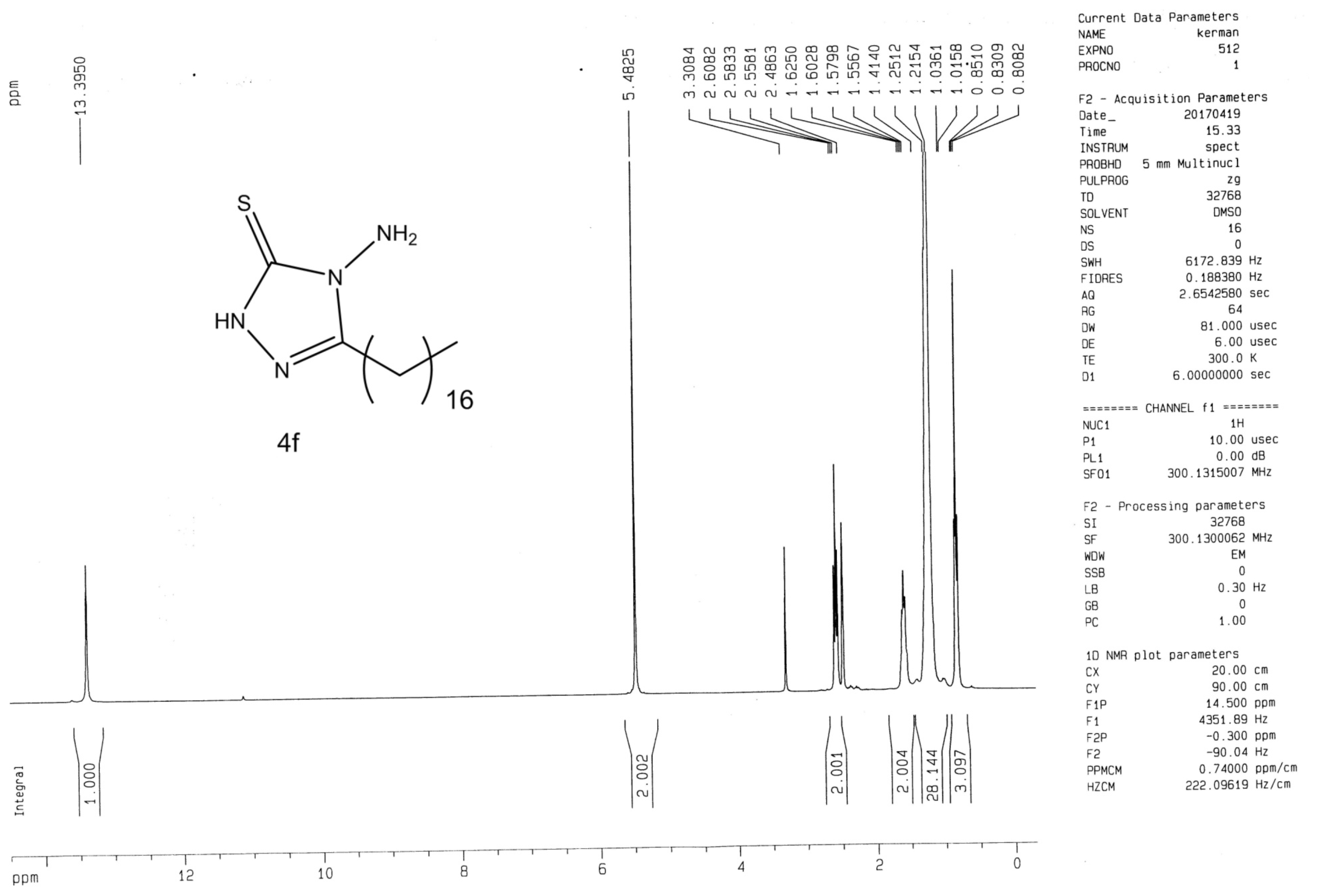

Supplement: Supplementary file 12 [file turkjchem-45-6-1805s12.tif]

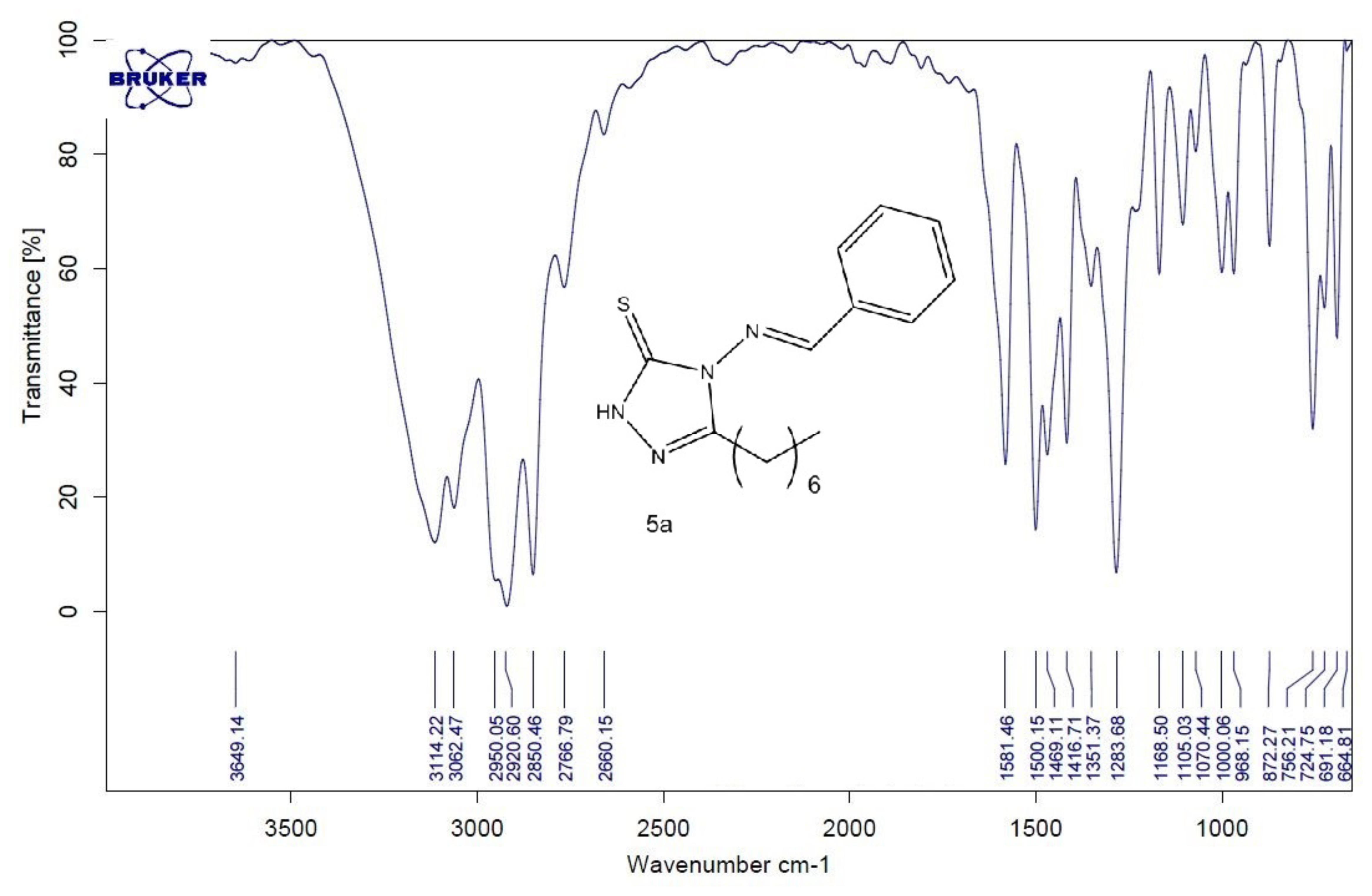

Supplement: Supplementary file 13 [file turkjchem-45-6-1805s13.tif]

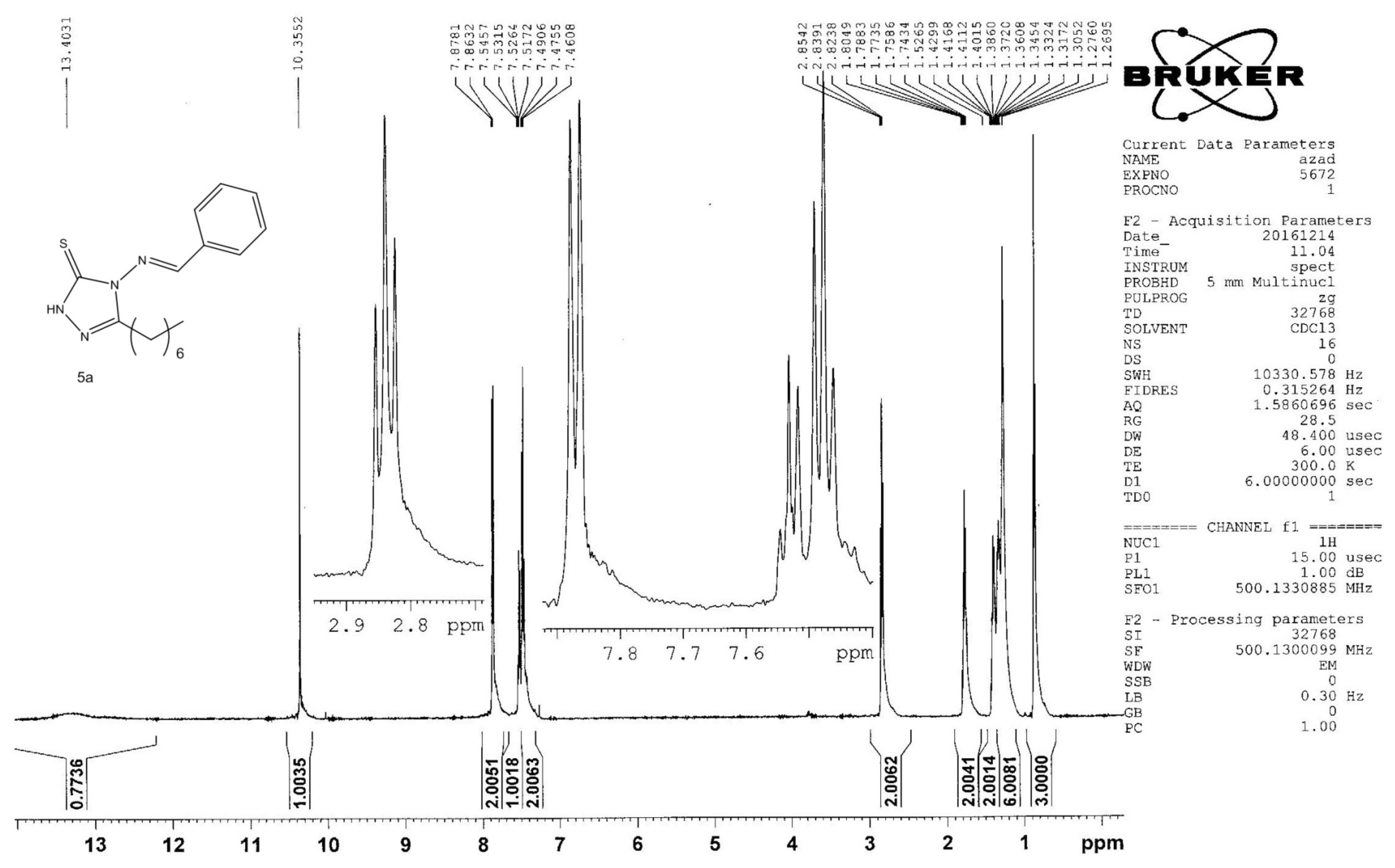

Supplement: Supplementary file 14 [file turkjchem-45-6-1805s14.tif]

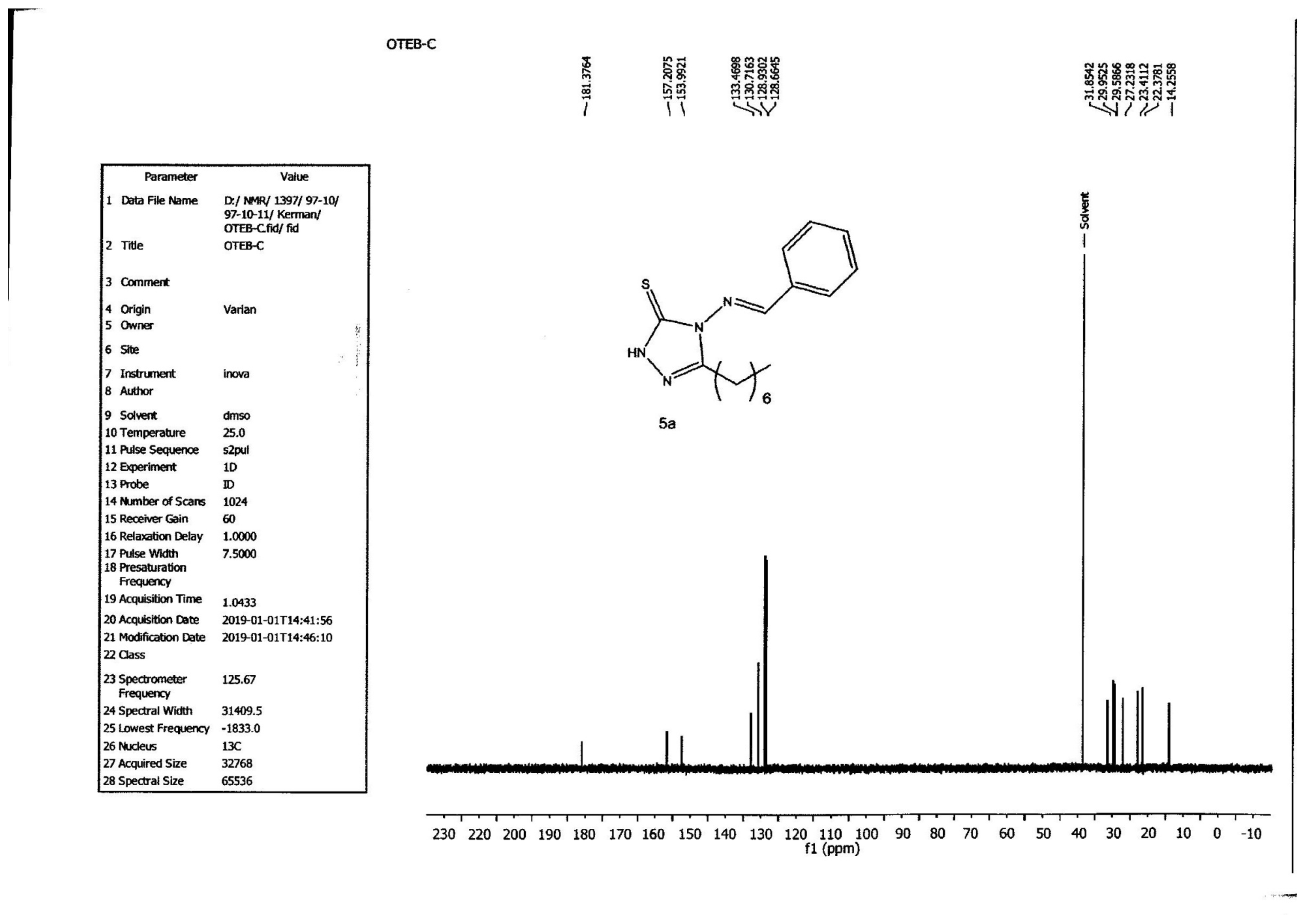

Supplement: Supplementary file 15 [file turkjchem-45-6-1805s15.tif]

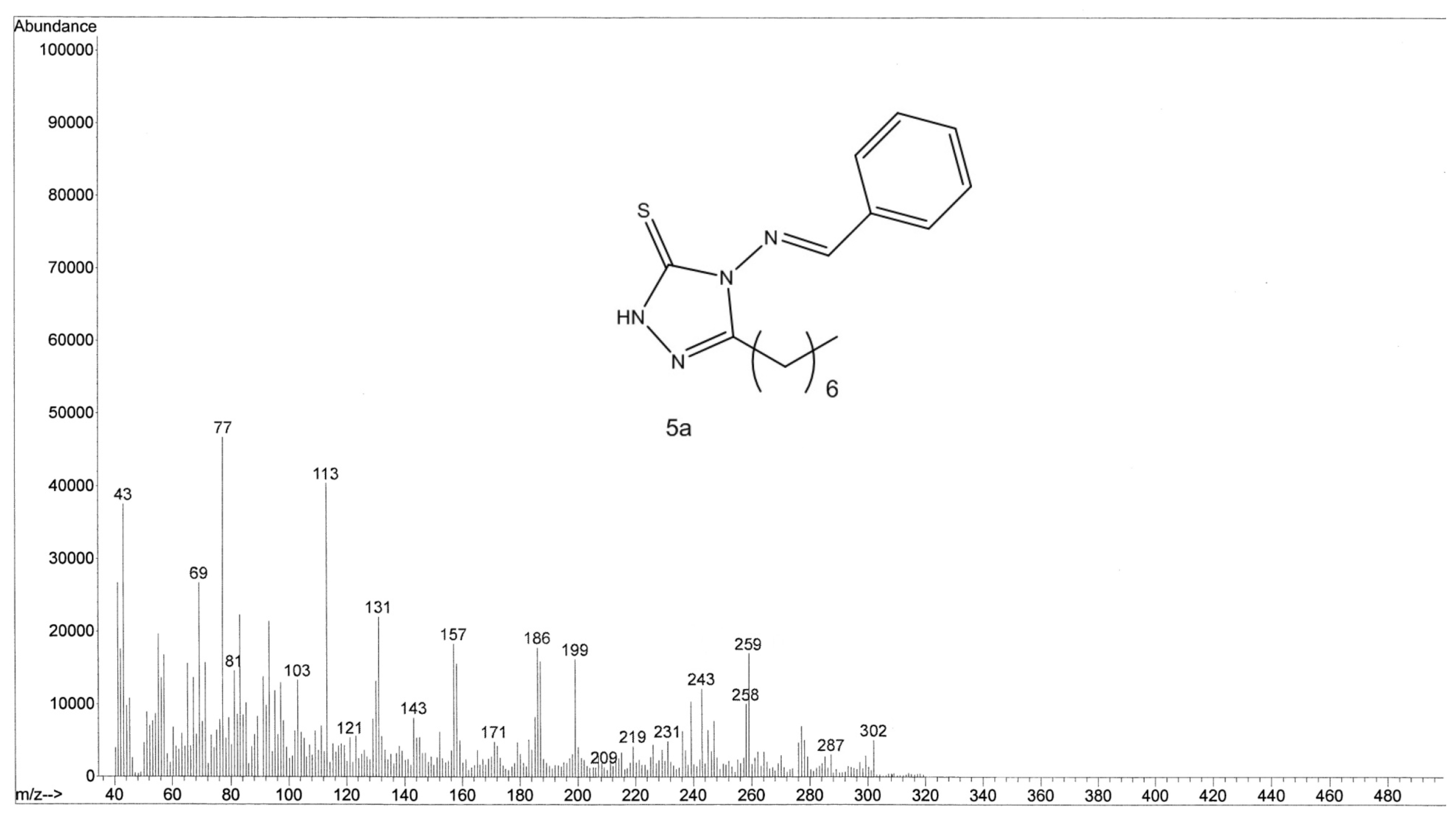

Supplement: Supplementary file 16 [file turkjchem-45-6-1805s16.tif]

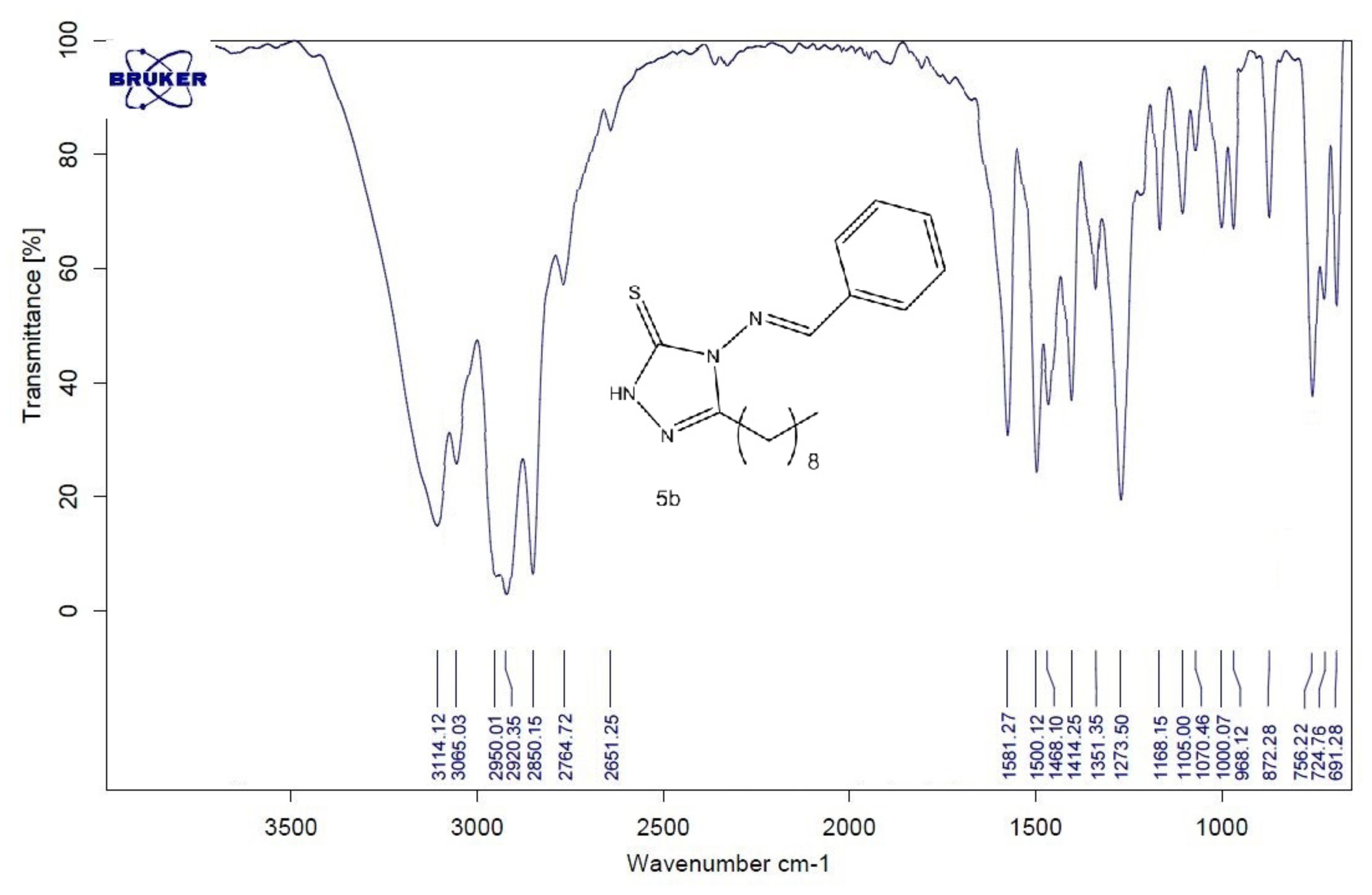

Supplement: Supplementary file 17 [file turkjchem-45-6-1805s17.tif]

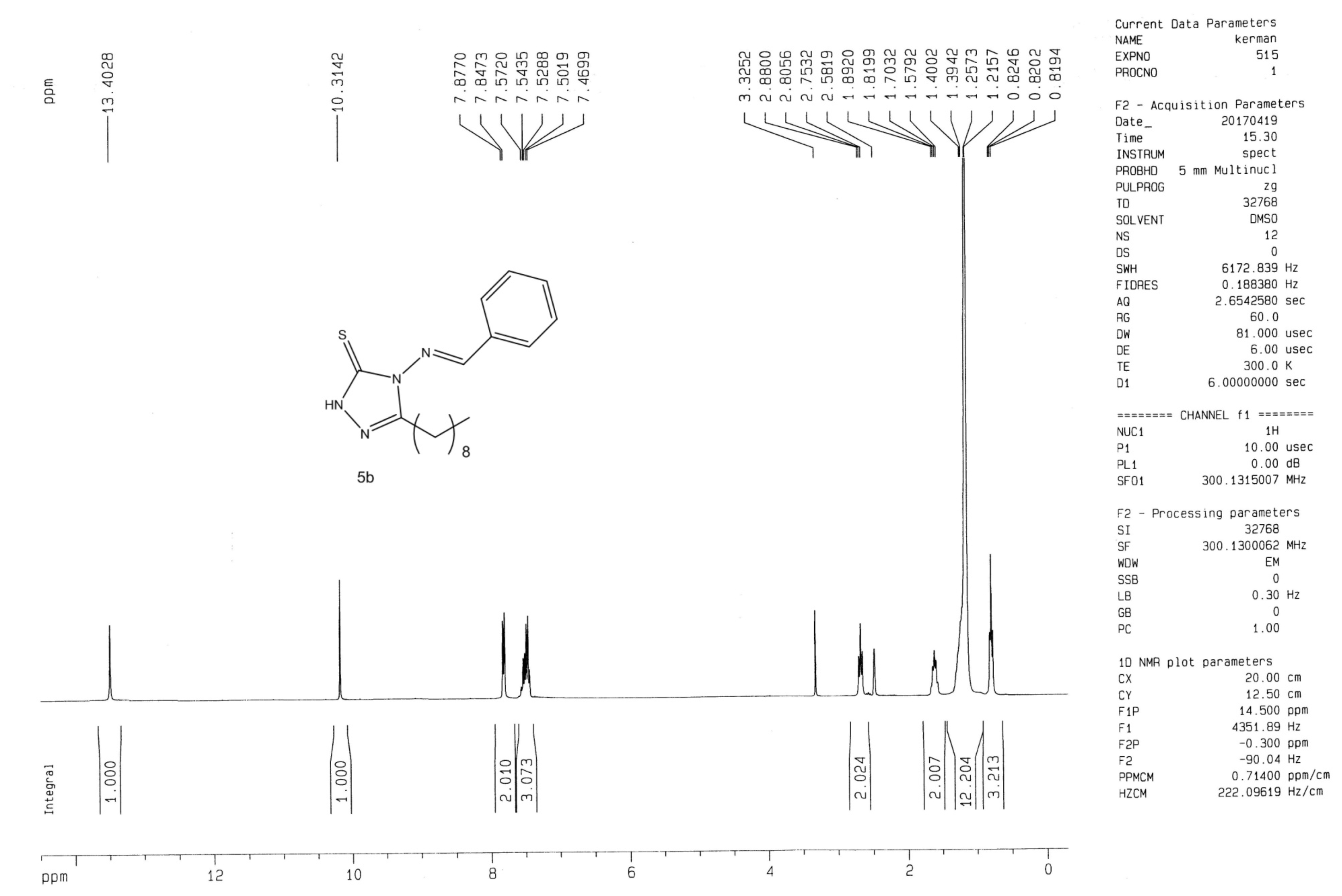

Supplement: Supplementary file 18 [file turkjchem-45-6-1805s18.tif]

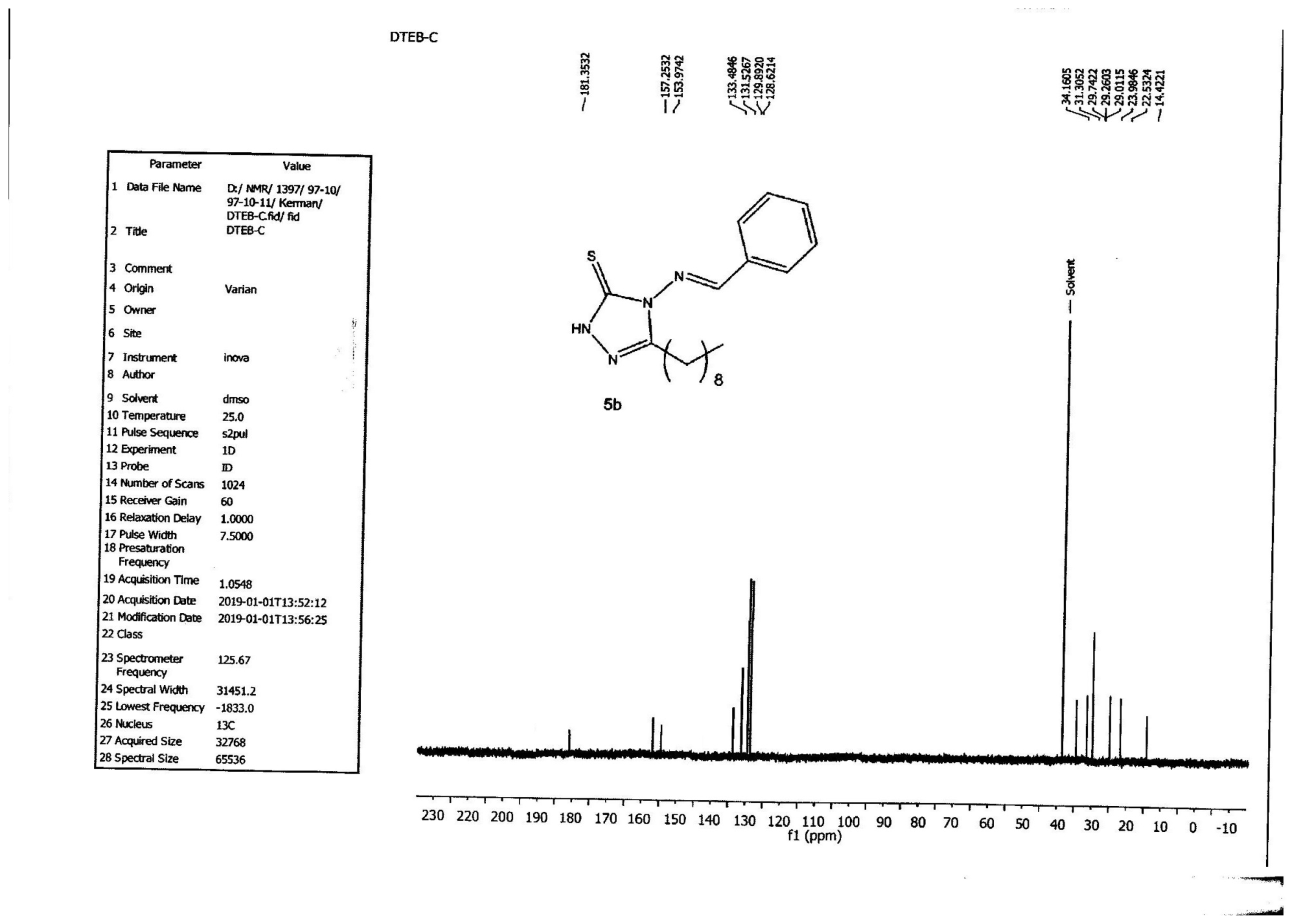

Supplement: Supplementary file 19 [file turkjchem-45-6-1805s19.tif]

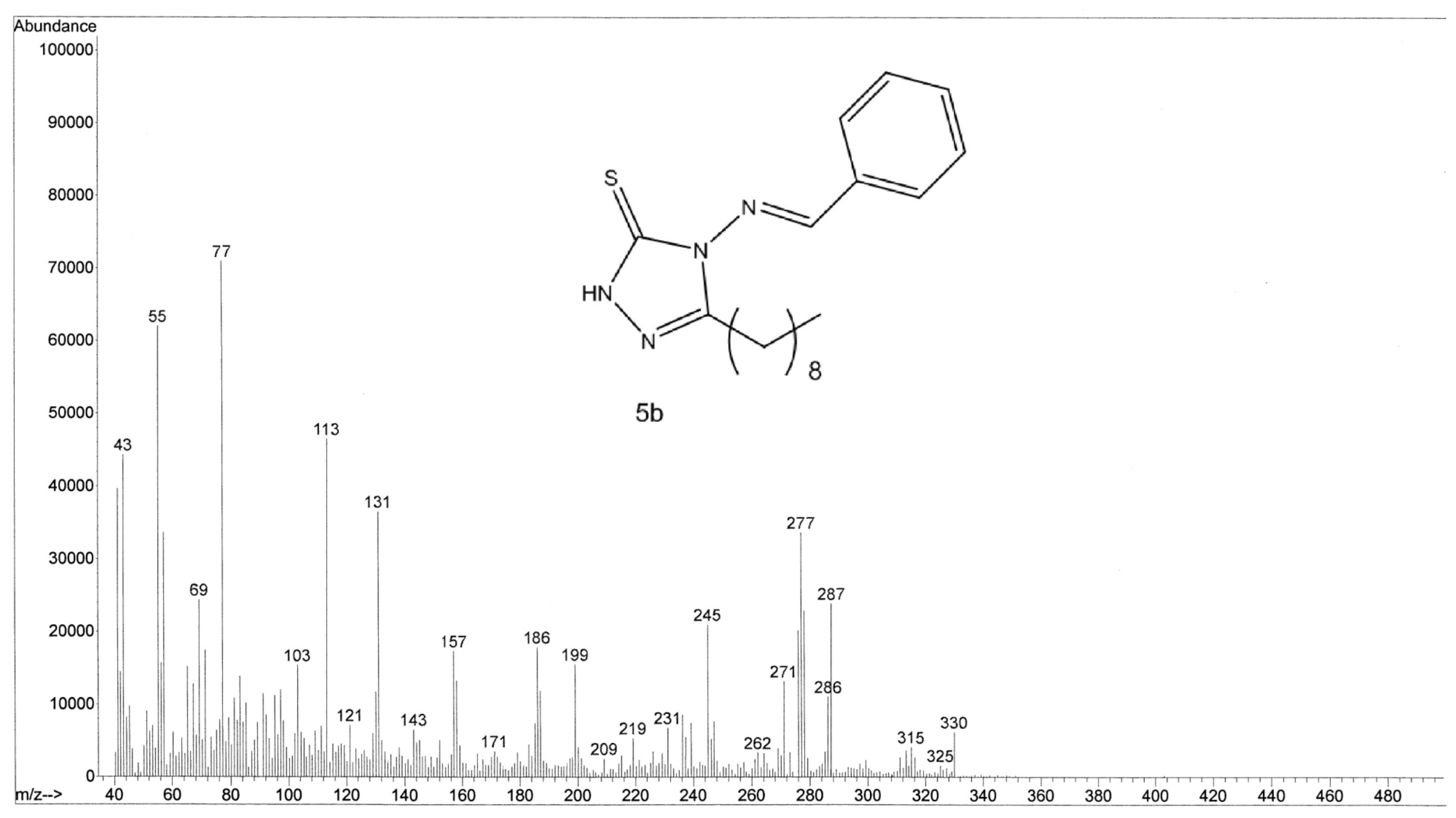

Supplement: Supplementary file 20 [file turkjchem-45-6-1805s20.tif]

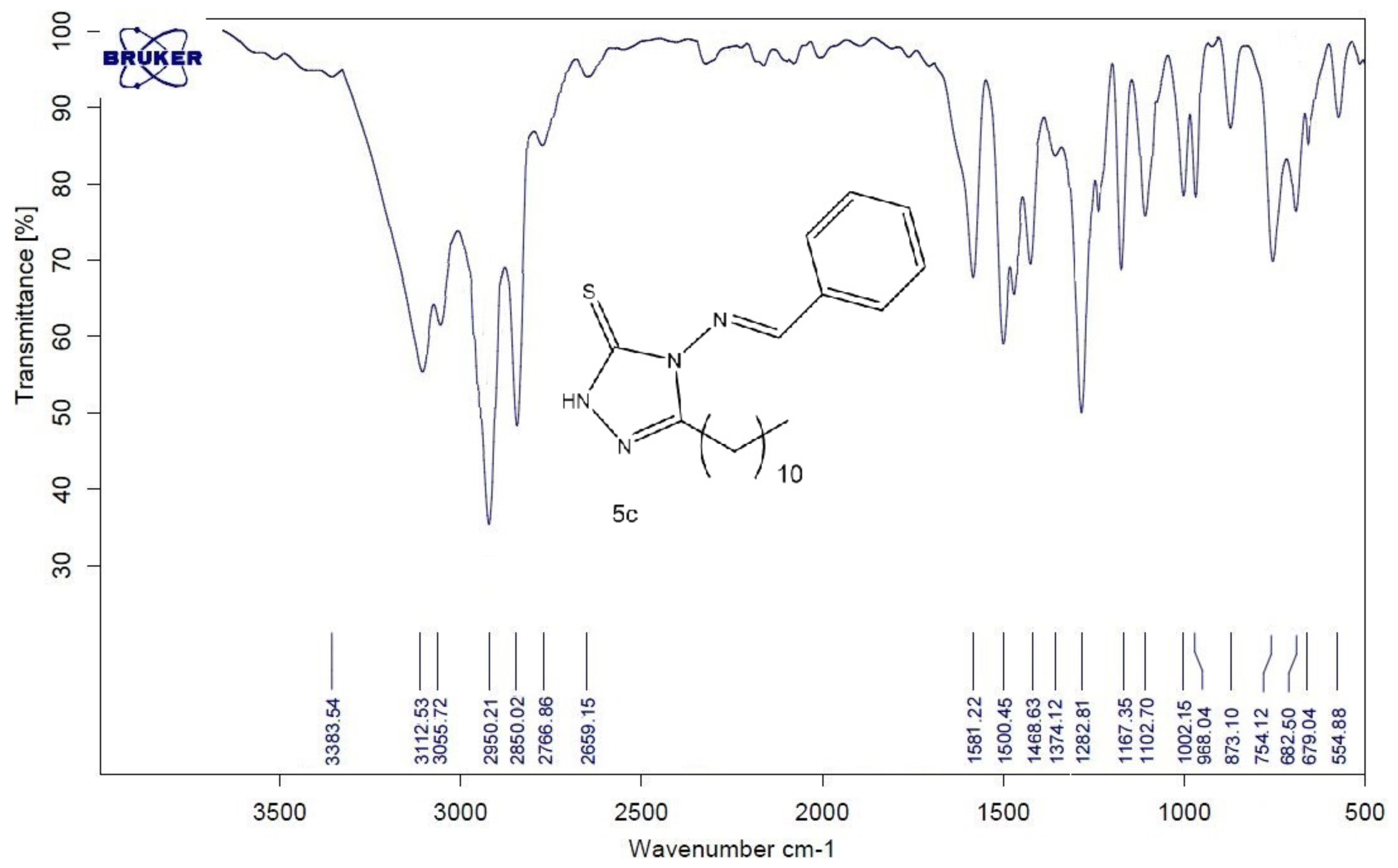

Supplement: Supplementary file 21 [file turkjchem-45-6-1805s21.tif]

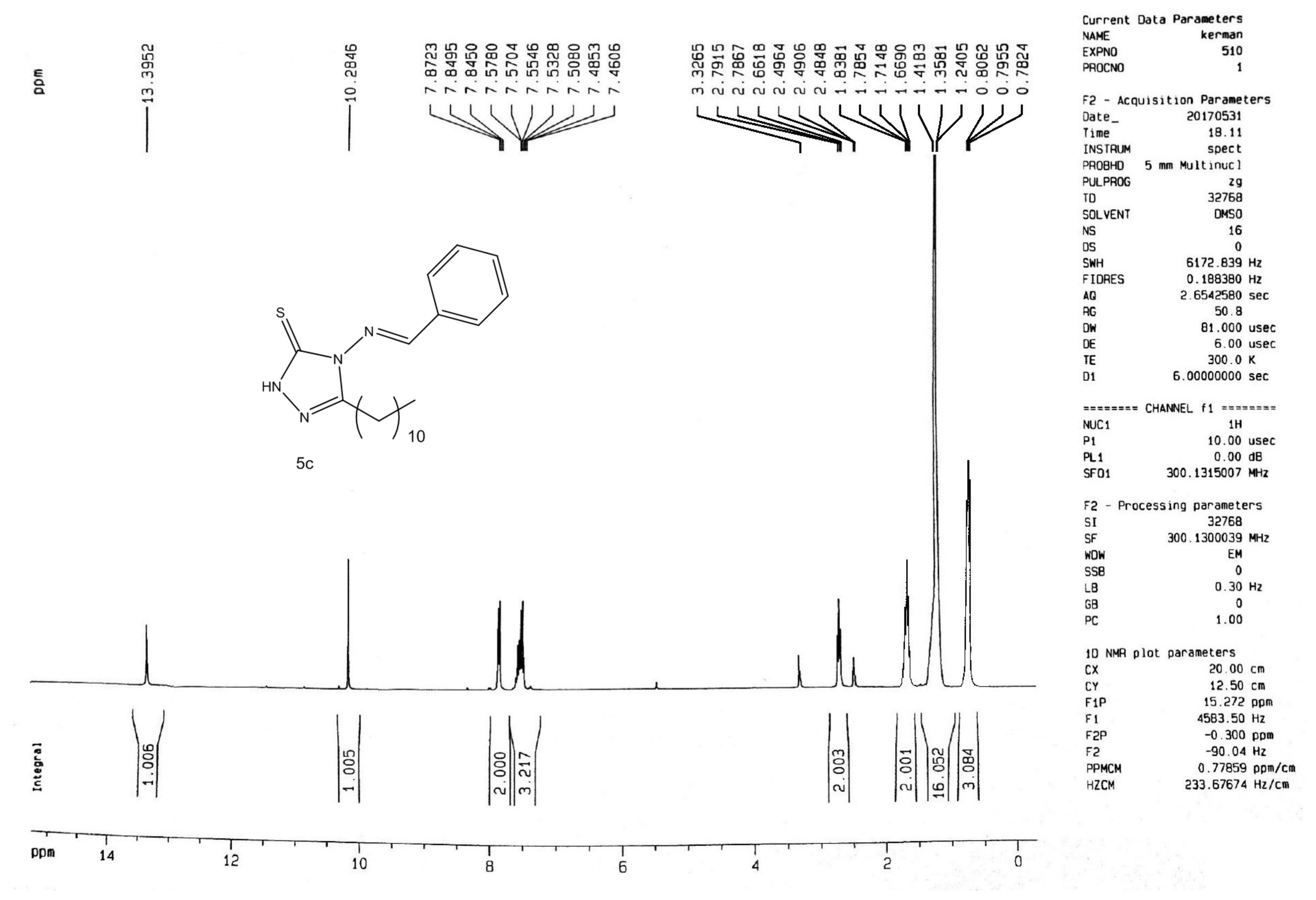

Supplement: Supplementary file 22 [file turkjchem-45-6-1805s22.tif]

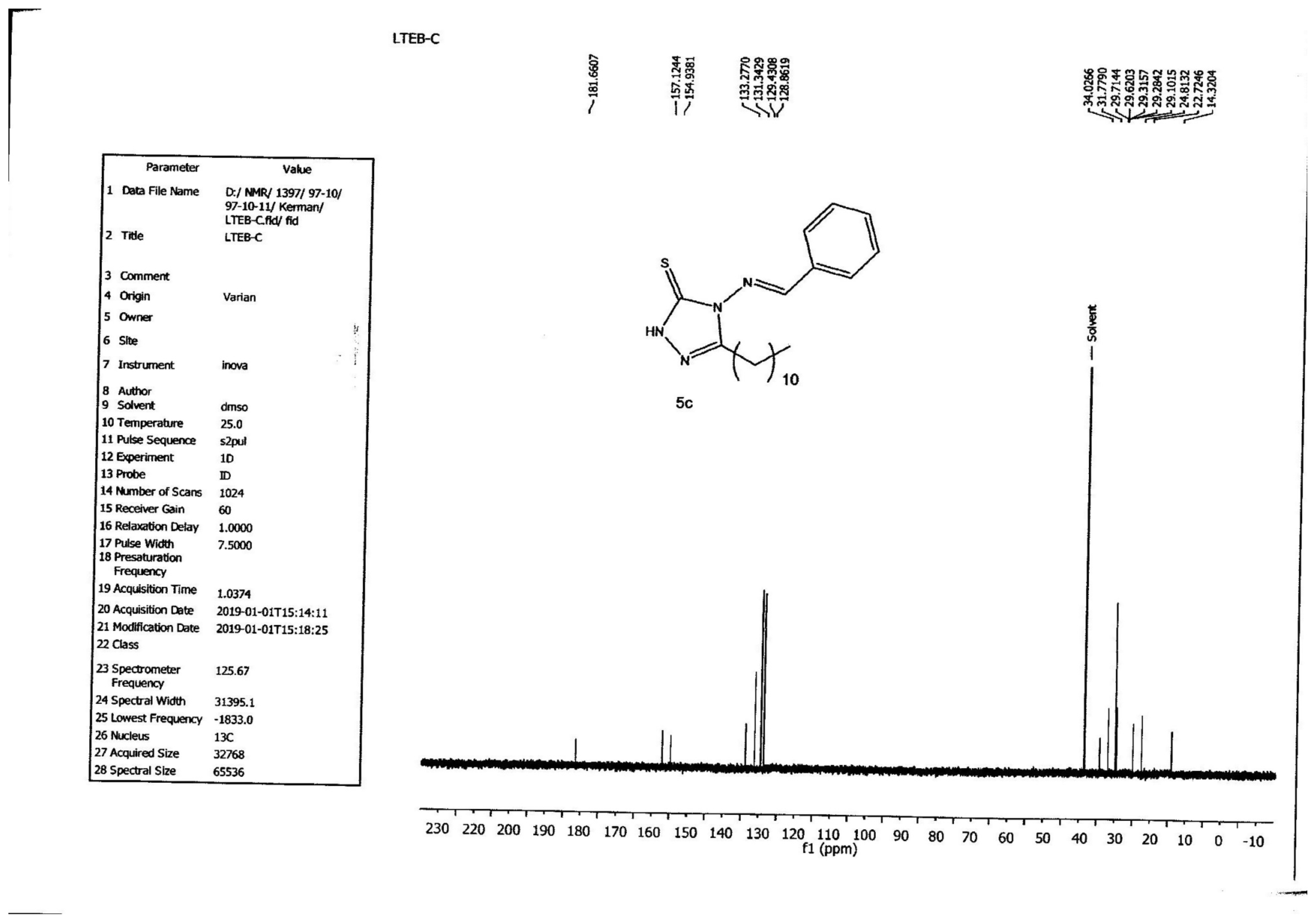

Supplement: Supplementary file 23 [file turkjchem-45-6-1805s23.tif]

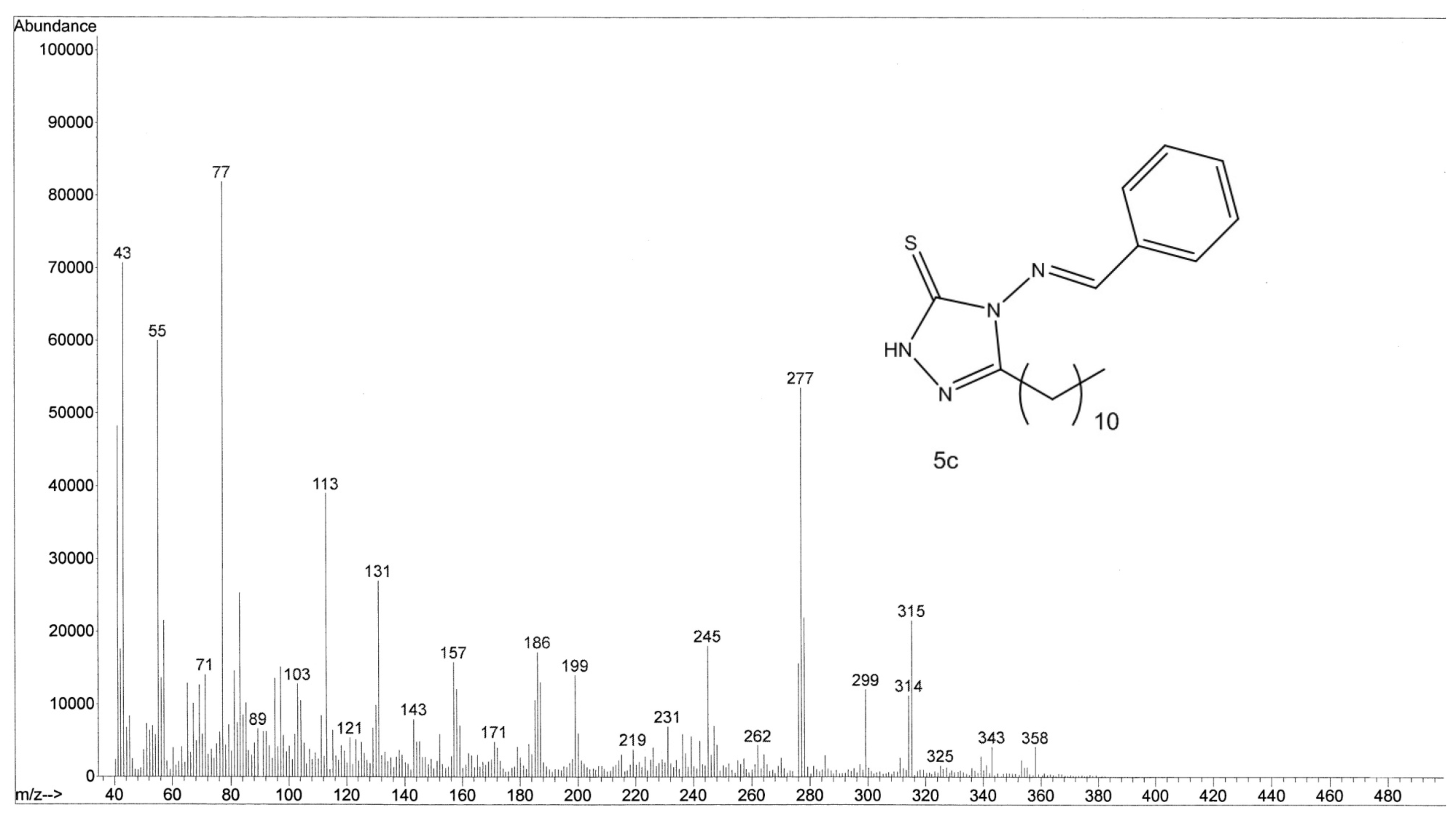

Supplement: Supplementary file 24 [file turkjchem-45-6-1805s24.tif]

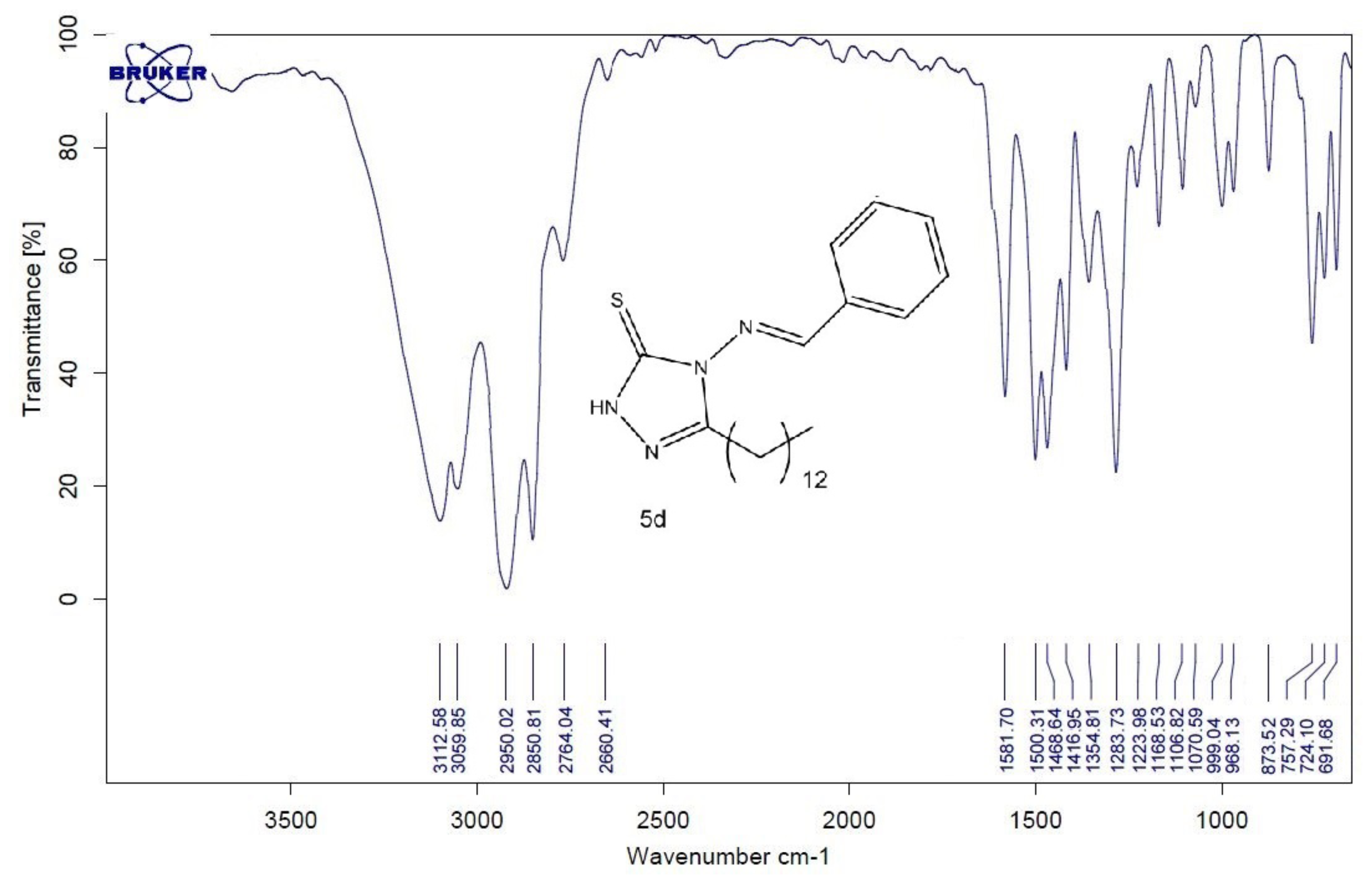

Supplement: Supplementary file 25 [file turkjchem-45-6-1805s25.tif]

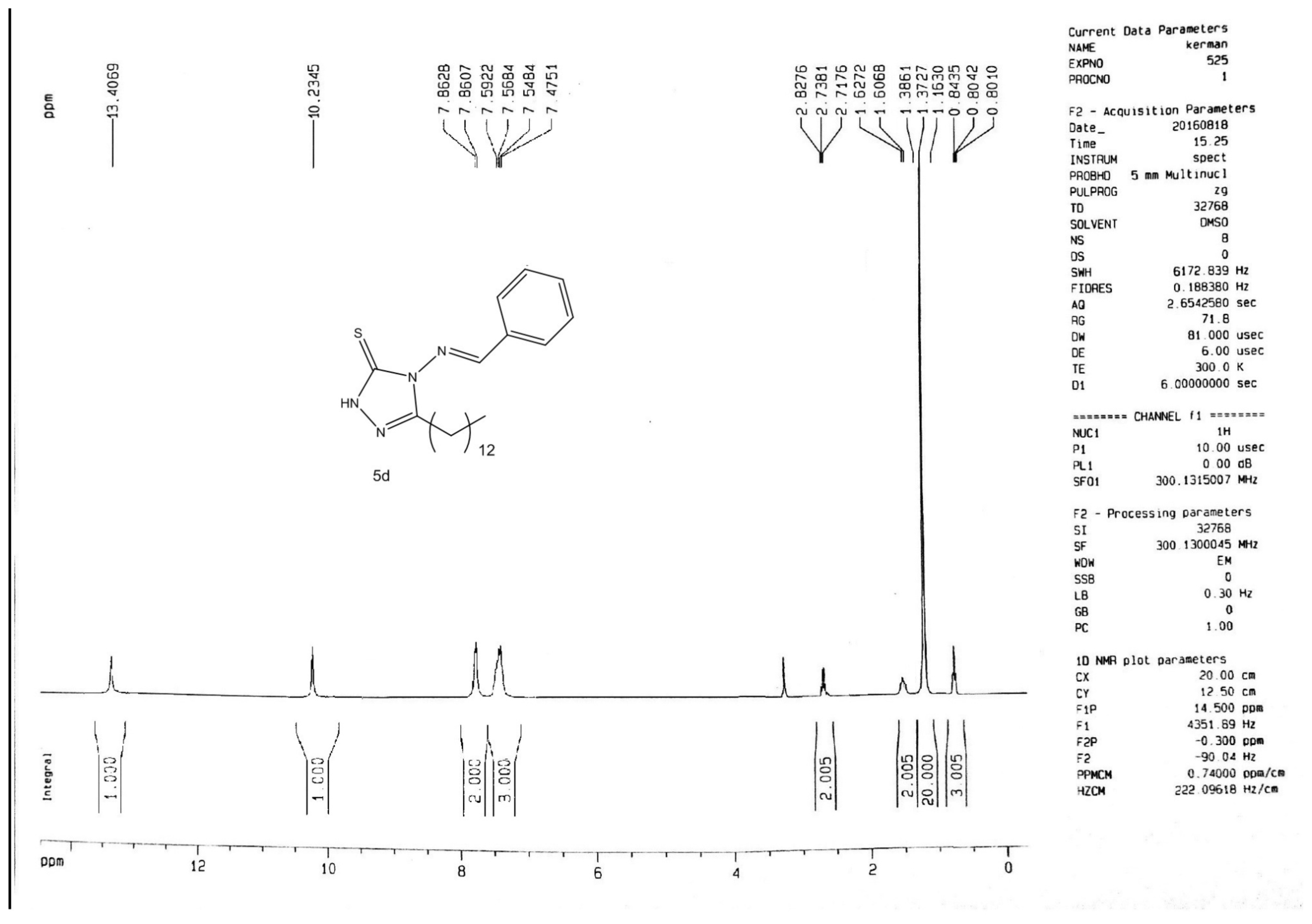

Supplement: Supplementary file 26 [file turkjchem-45-6-1805s26.tif]

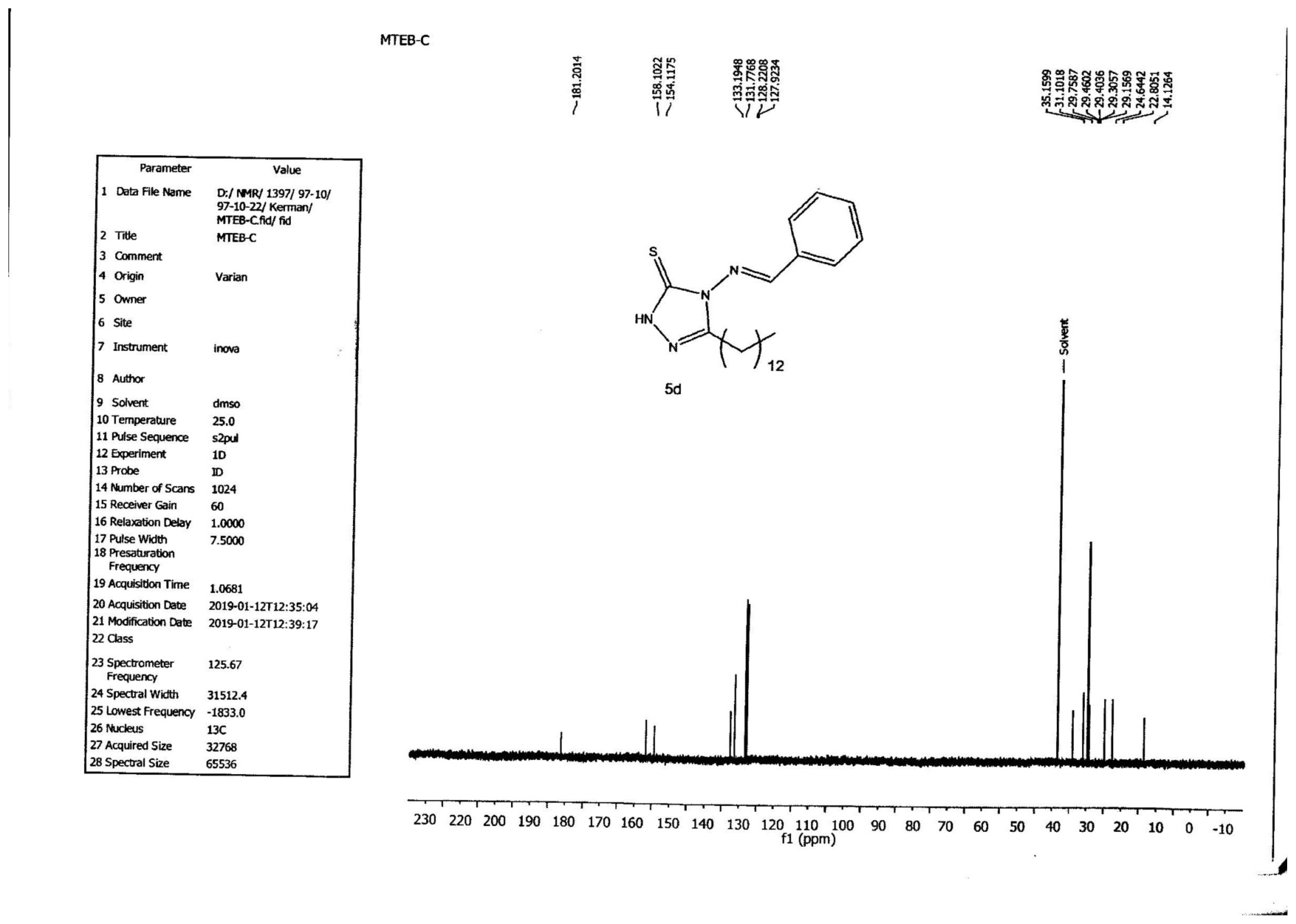

Supplement: Supplementary file 27 [file turkjchem-45-6-1805s27.tif]

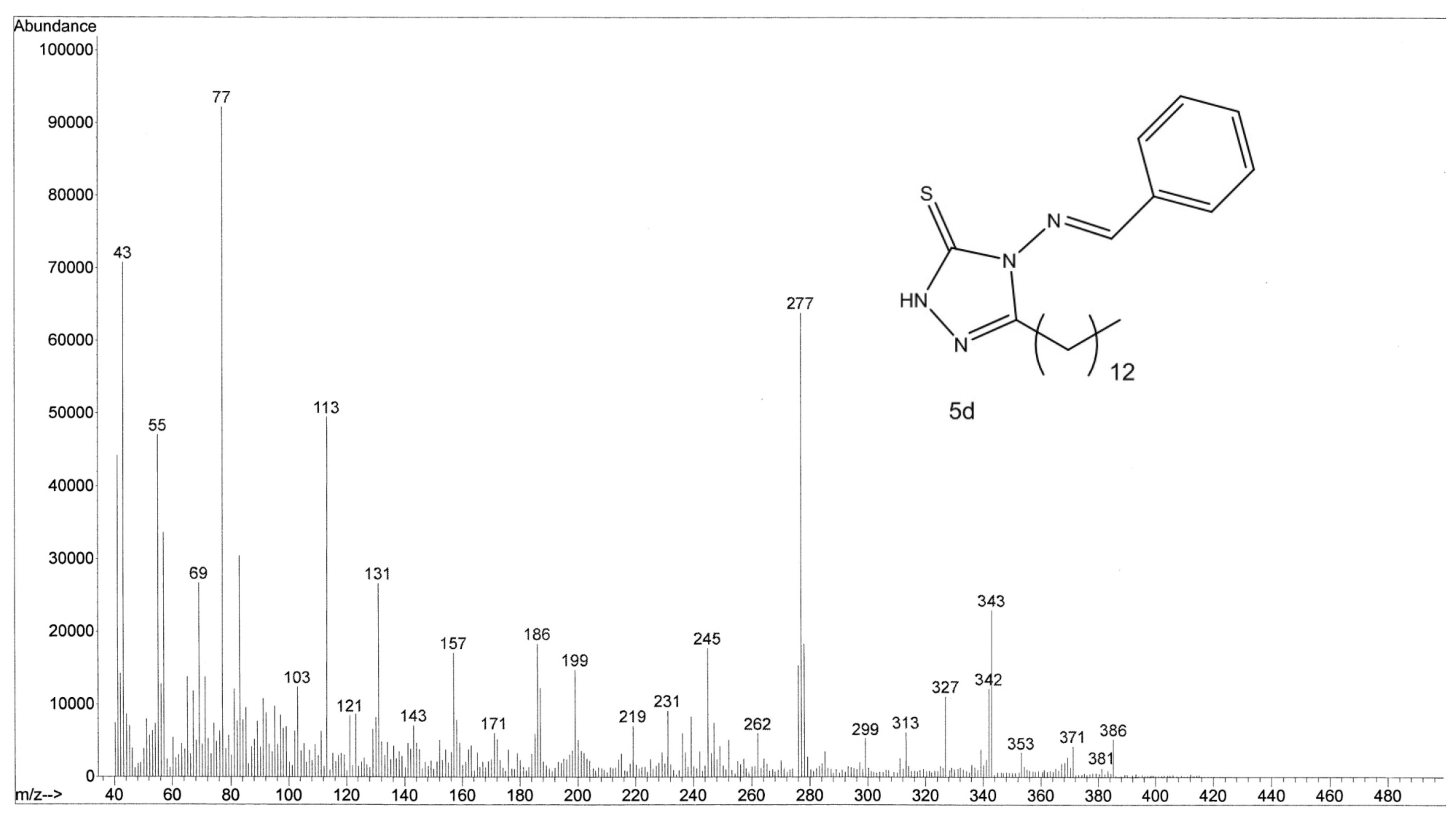

Supplement: Supplementary file 28 [file turkjchem-45-6-1805s28.tif]

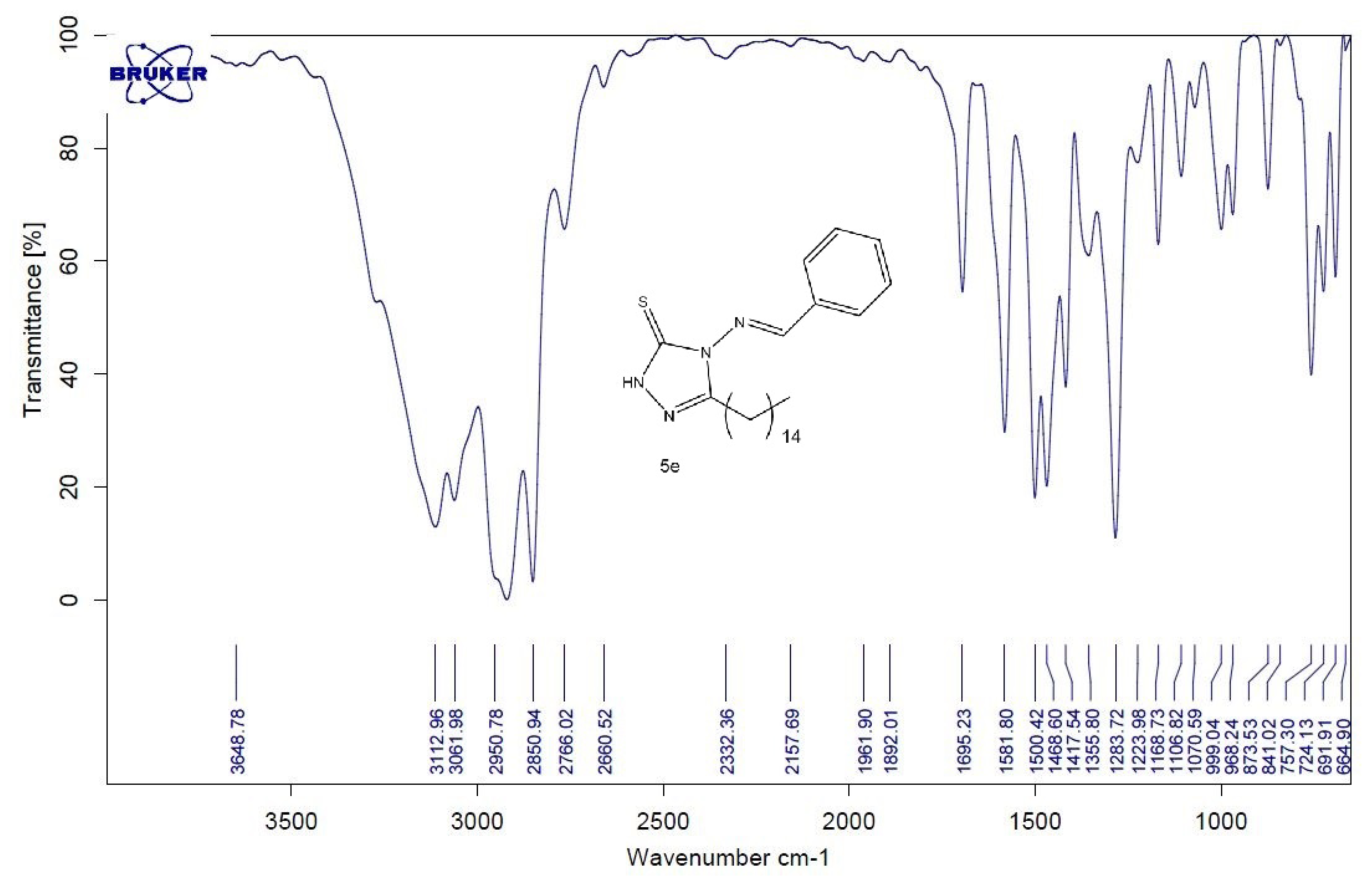

Supplement: Supplementary file 29 [file turkjchem-45-6-1805s29.tif]

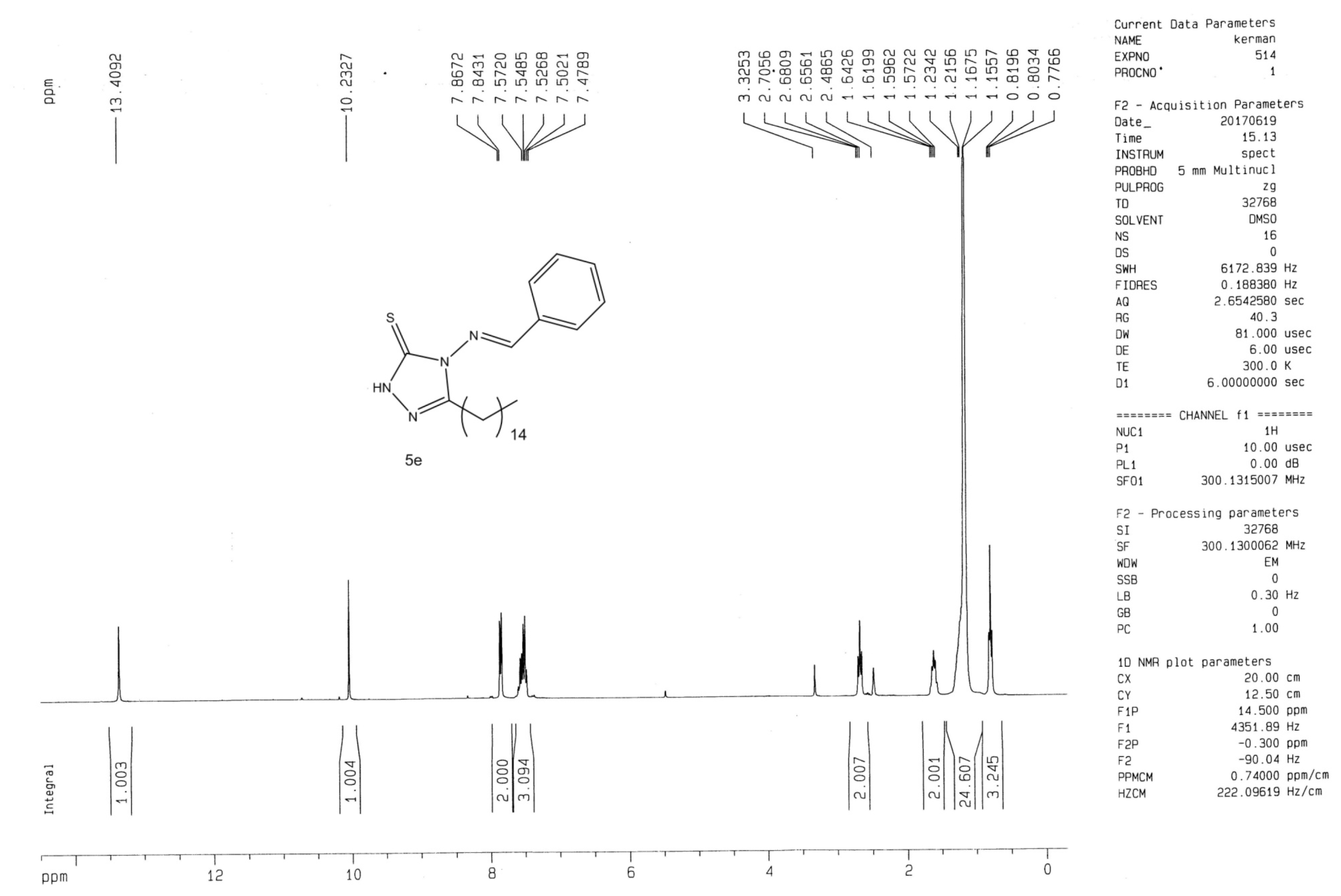

Supplement: Supplementary file 30 [file turkjchem-45-6-1805s30.tif]

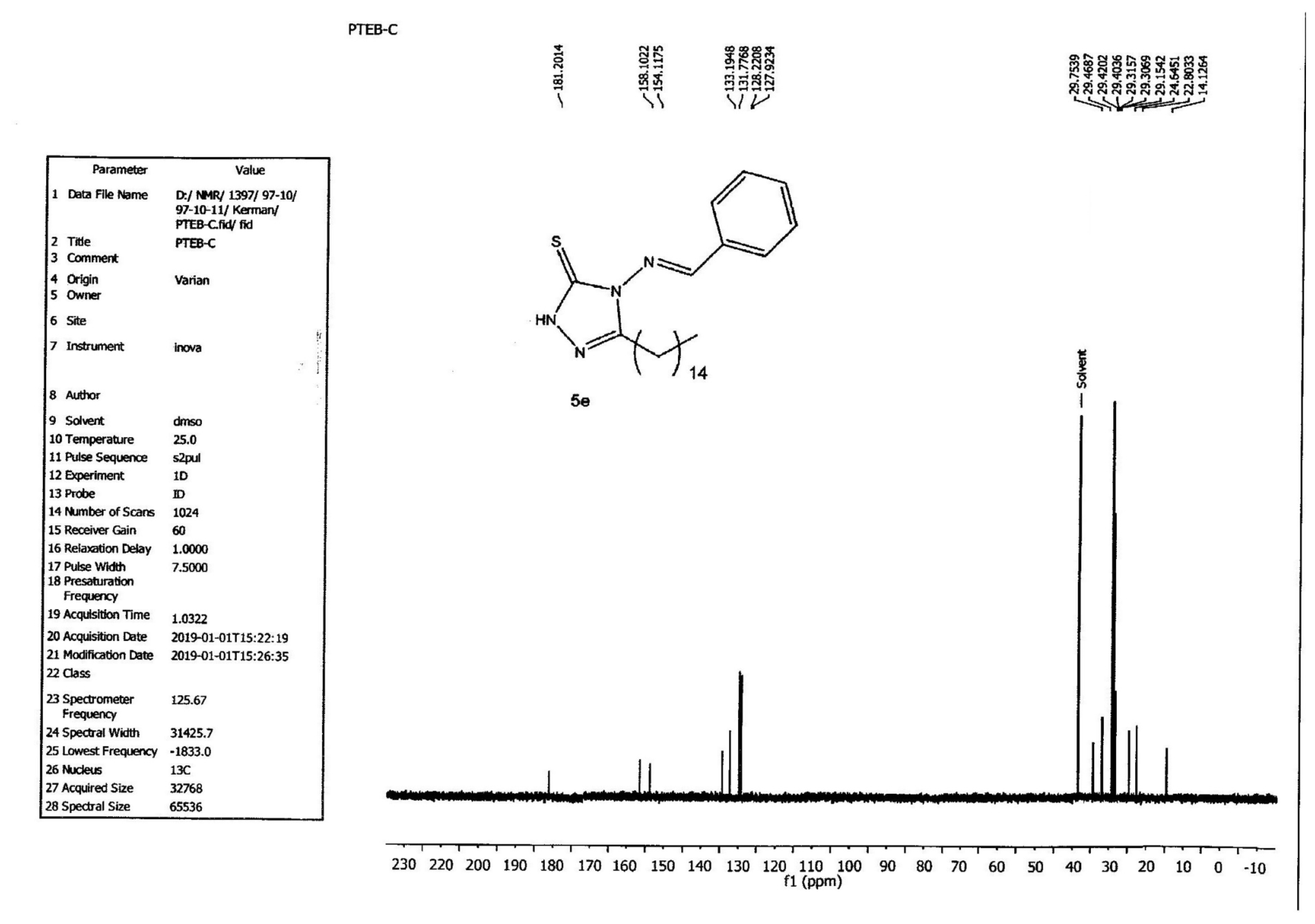

Supplement: Supplementary file 31 [file turkjchem-45-6-1805s31.tif]

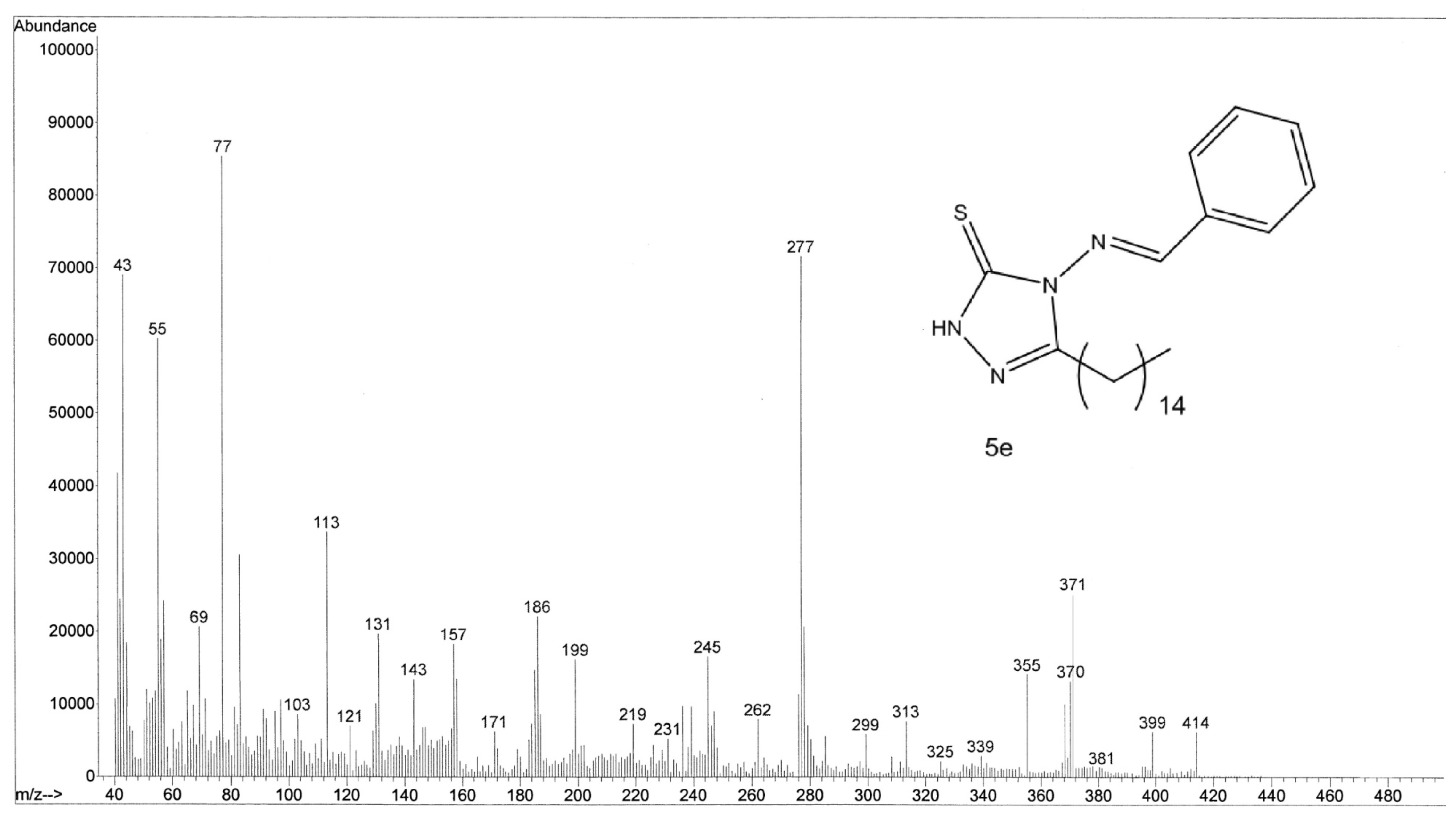

Supplement: Supplementary file 32 [file turkjchem-45-6-1805s32.tif]

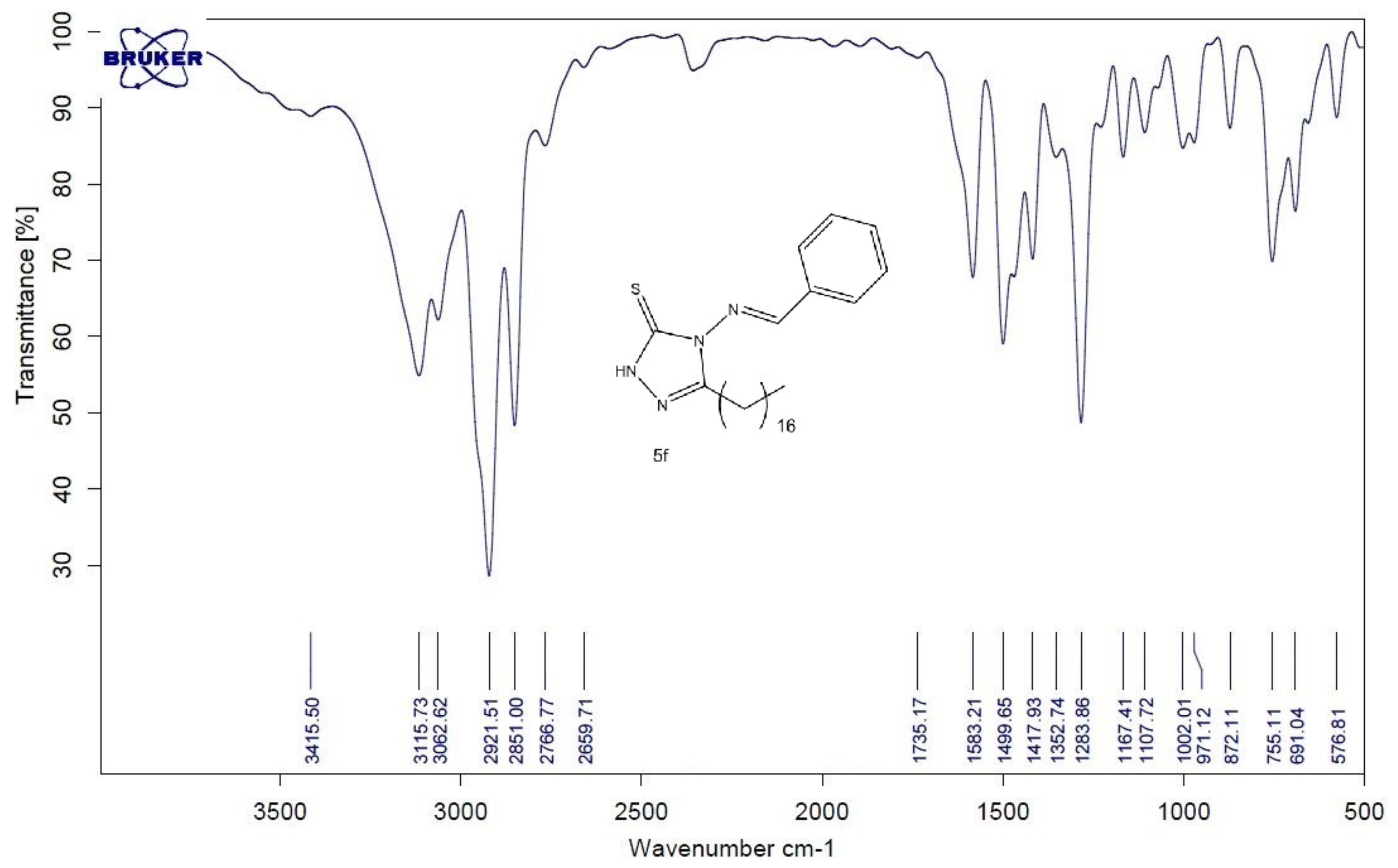

Supplement: Supplementary file 33 [file turkjchem-45-6-1805s33.tif]

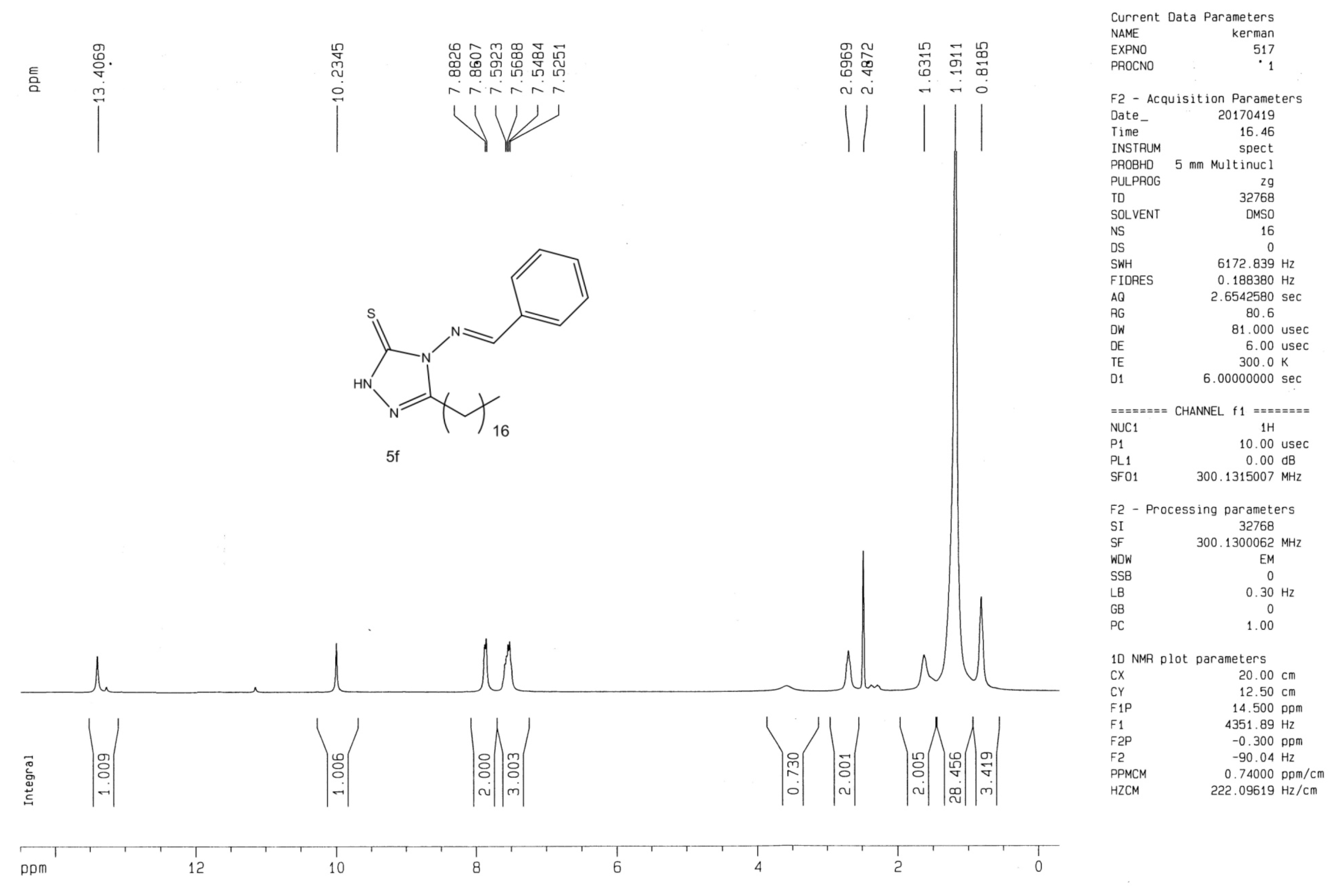

Supplement: Supplementary file 34 [file turkjchem-45-6-1805s34.tif]

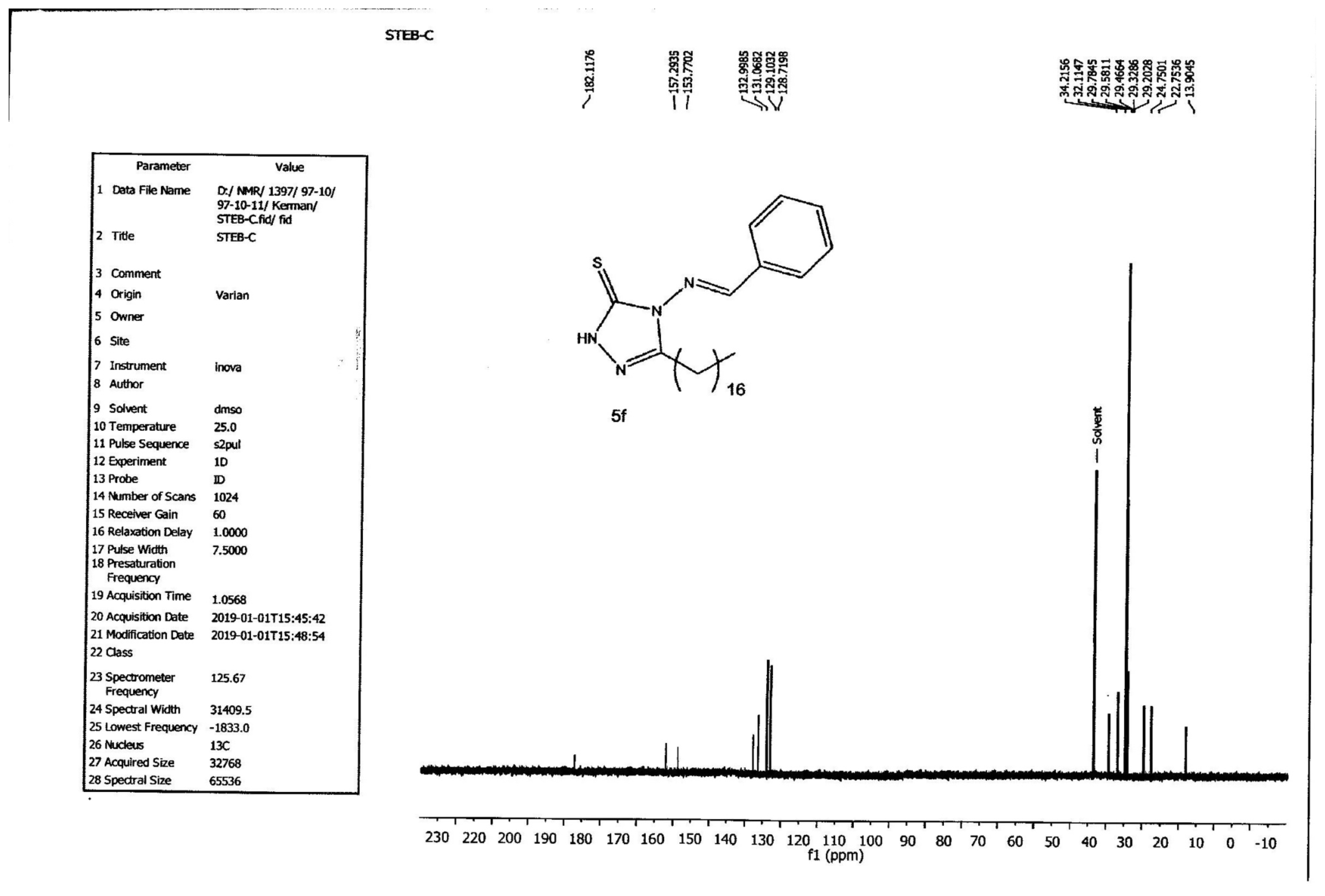

Supplement: Supplementary file 35 [file turkjchem-45-6-1805s35.tif]

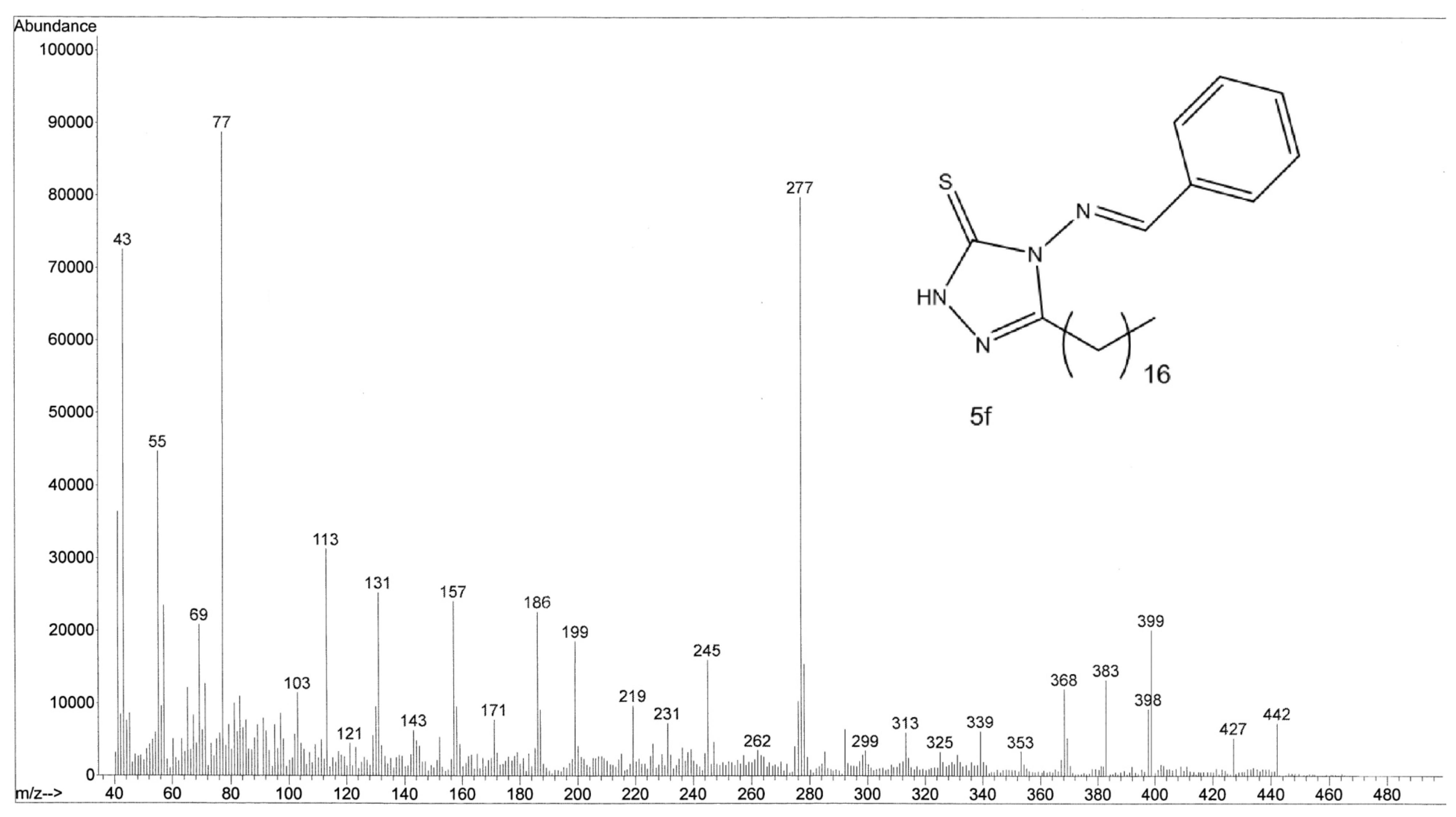

Supplement: Supplementary file 36 [file turkjchem-45-6-1805s36.tif]

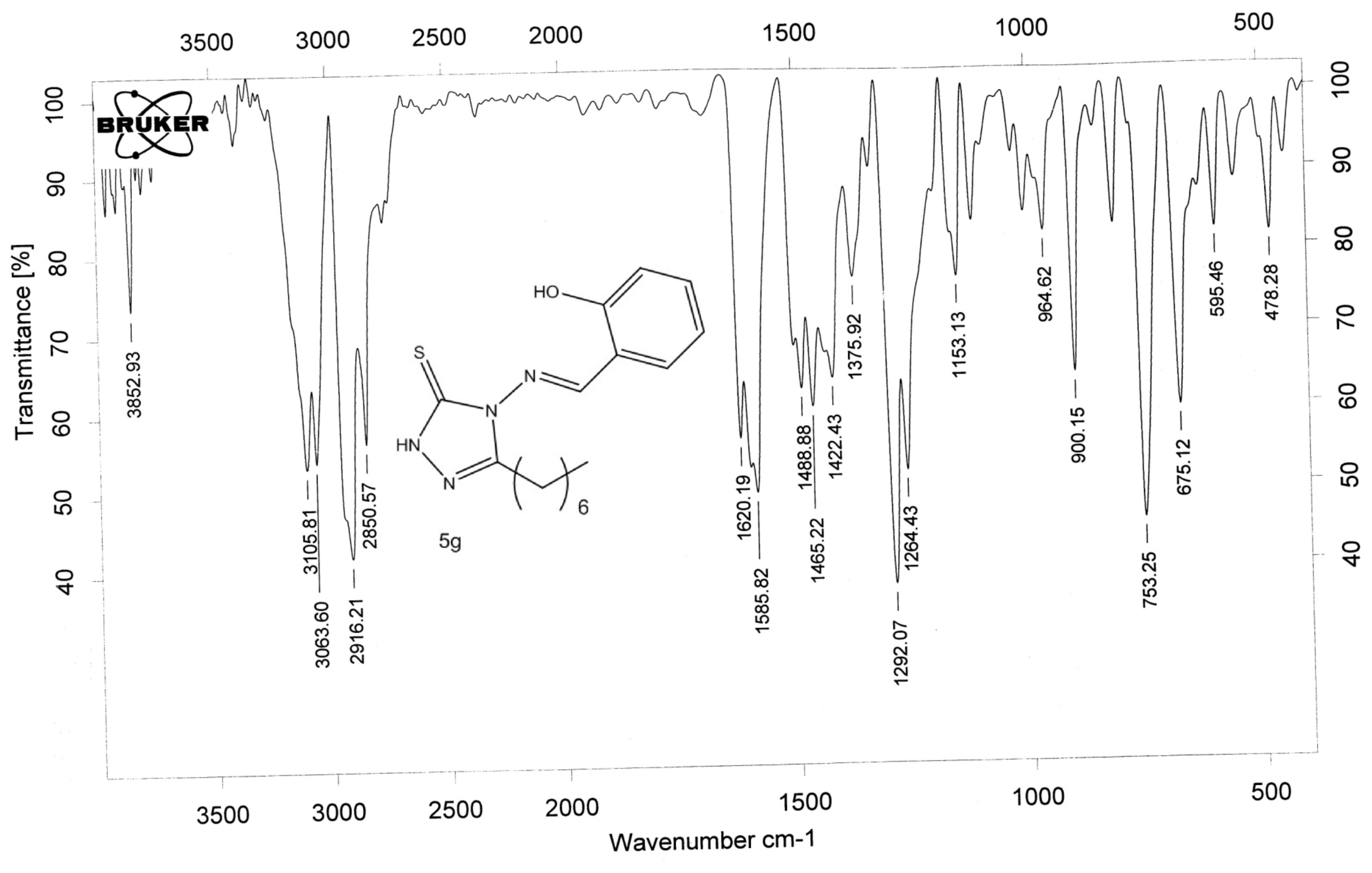

Supplement: Supplementary file 37 [file turkjchem-45-6-1805s37.tif]

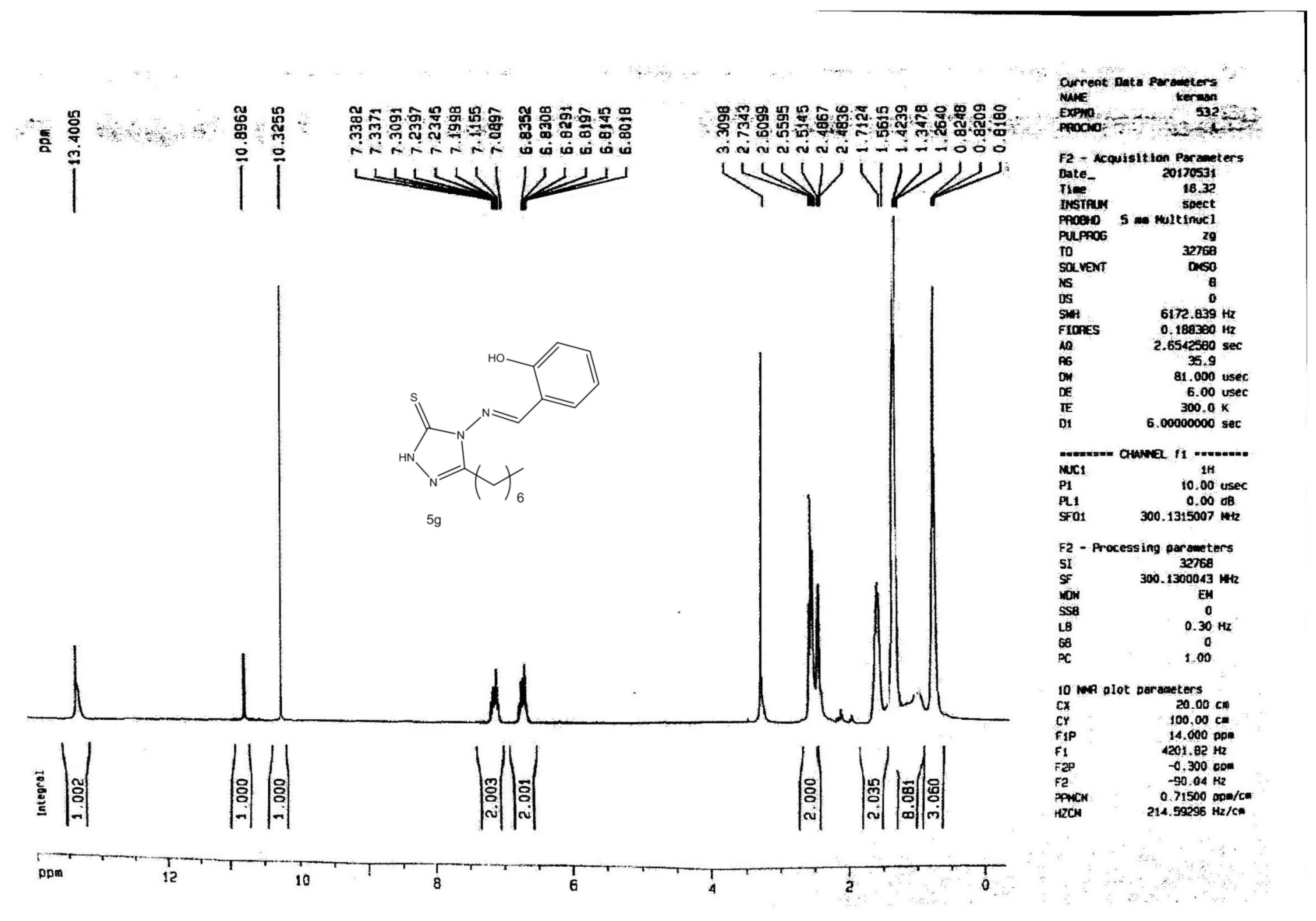

Supplement: Supplementary file 38 [file turkjchem-45-6-1805s38.tif]

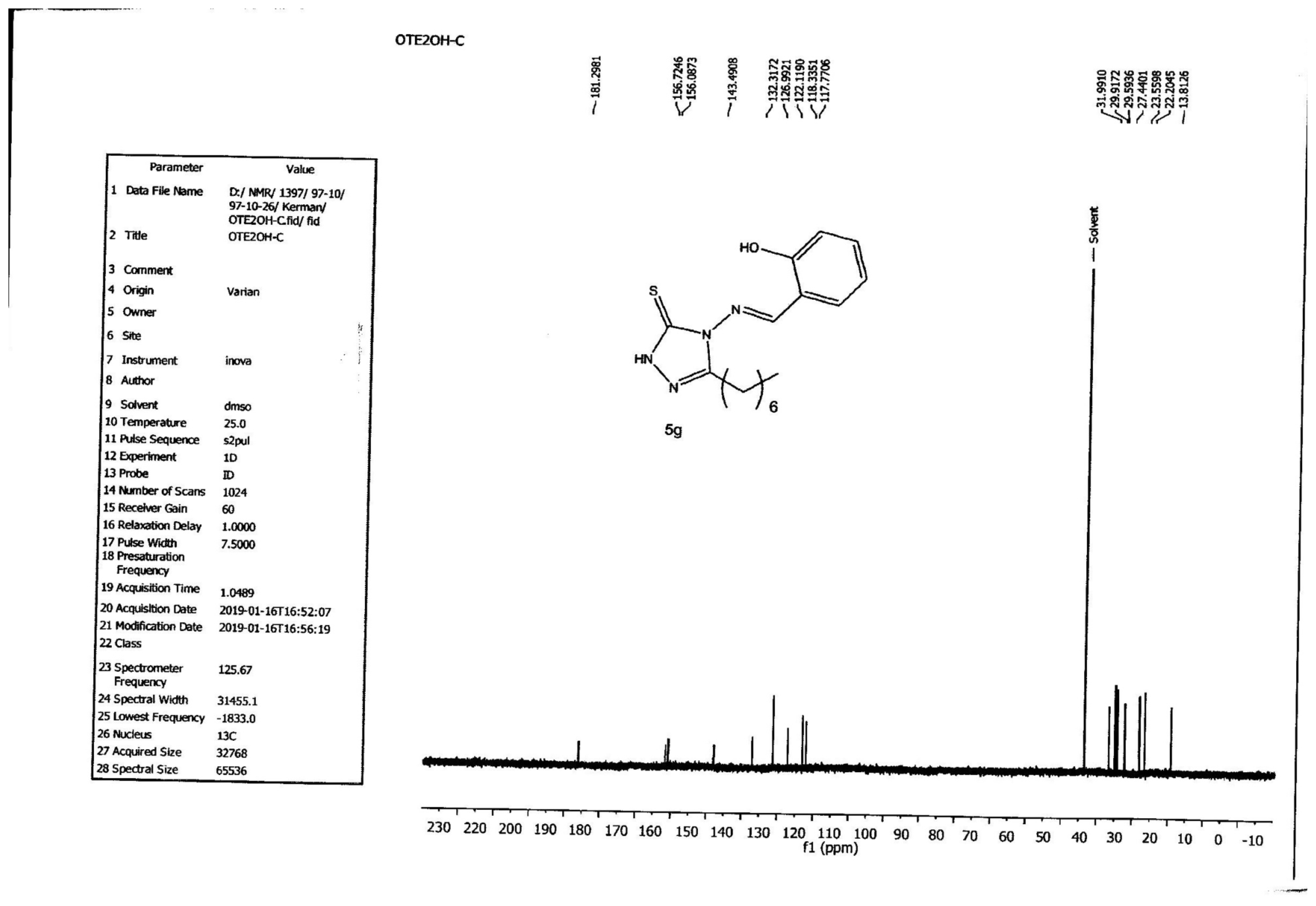

Supplement: Supplementary file 39 [file turkjchem-45-6-1805s39.tif]

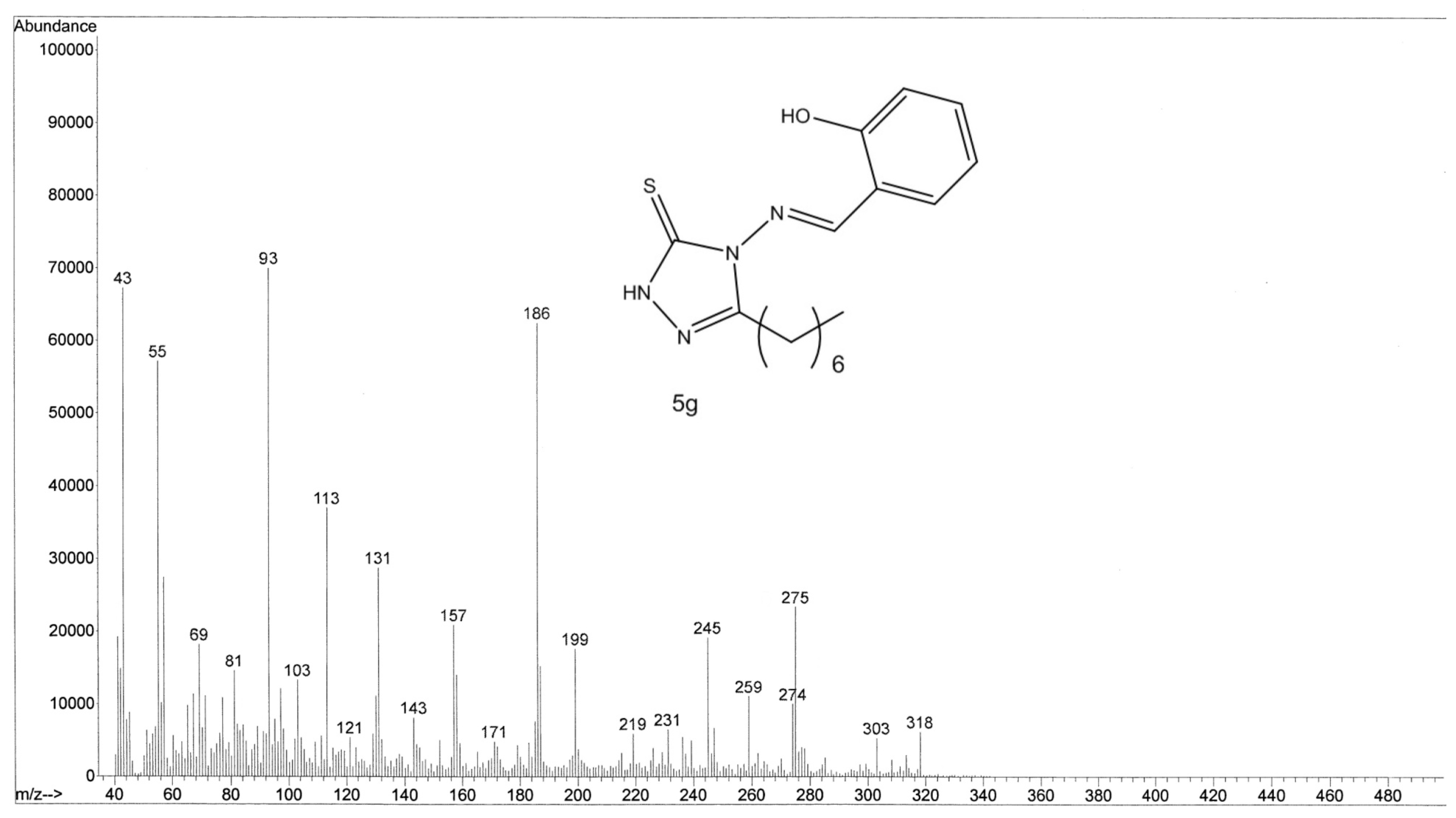

Supplement: Supplementary file 40 [file turkjchem-45-6-1805s40.tif]

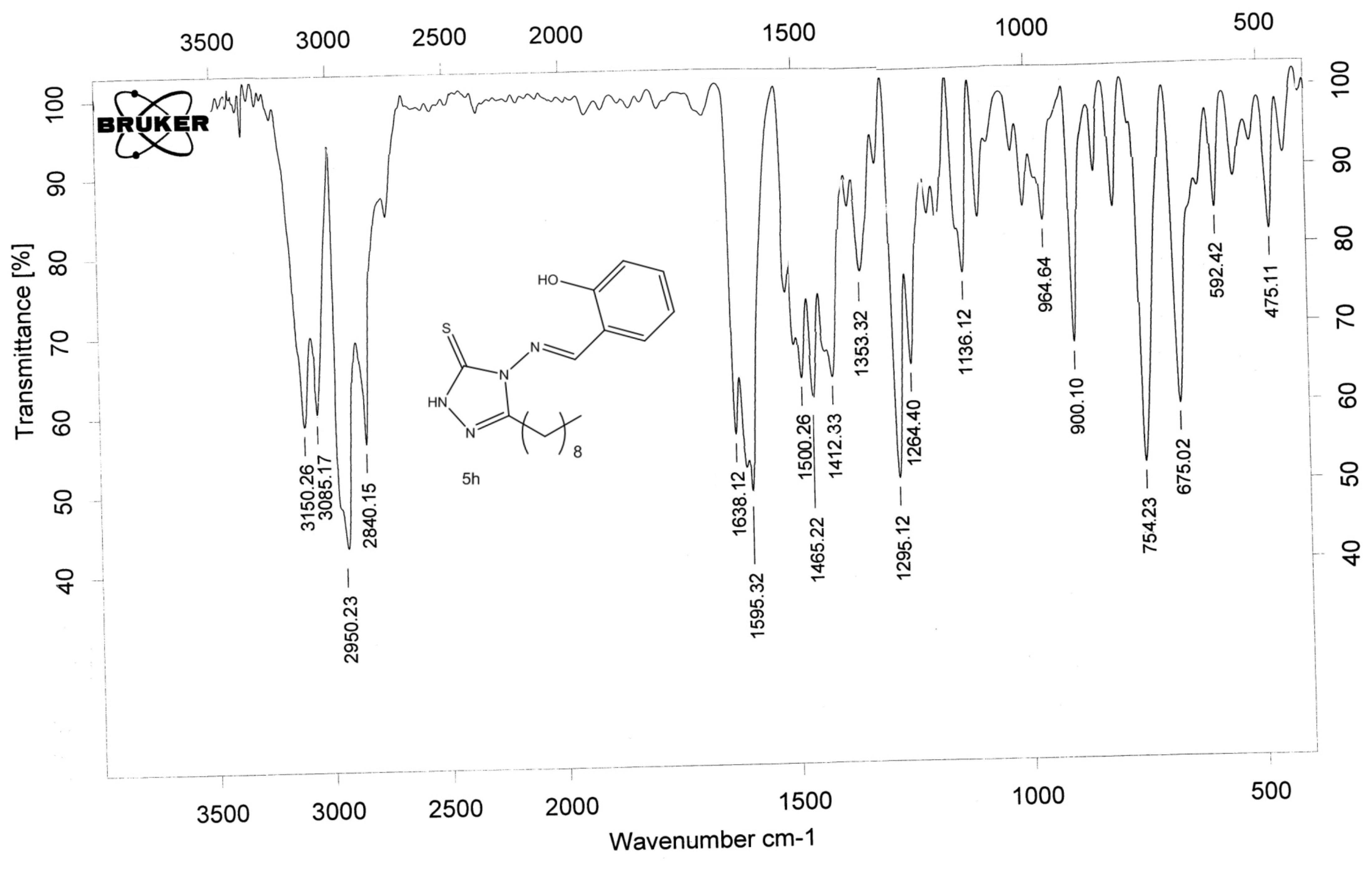

Supplement: Supplementary file 41 [file turkjchem-45-6-1805s41.tif]

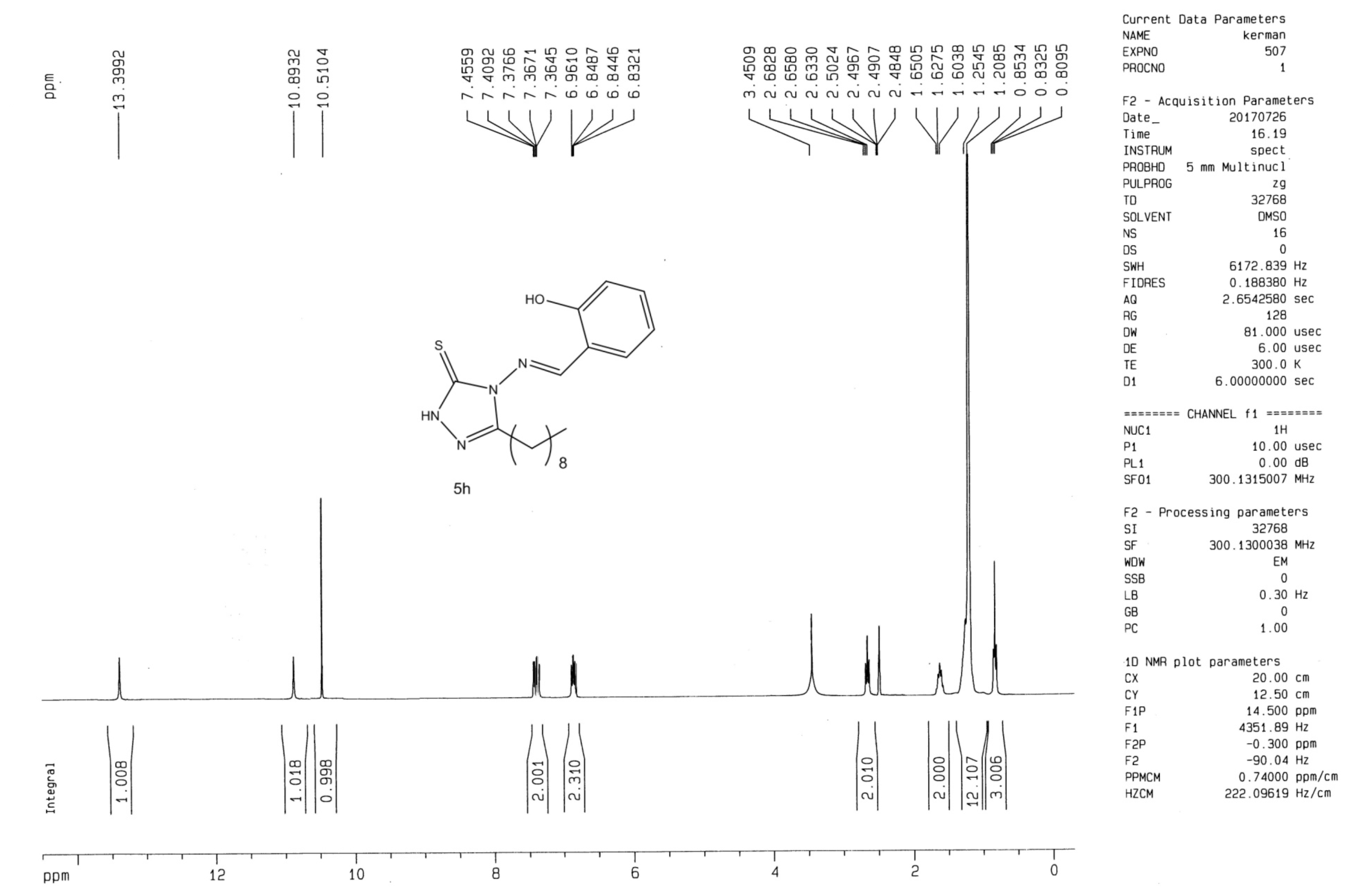

Supplement: Supplementary file 42 [file turkjchem-45-6-1805s42.tif]

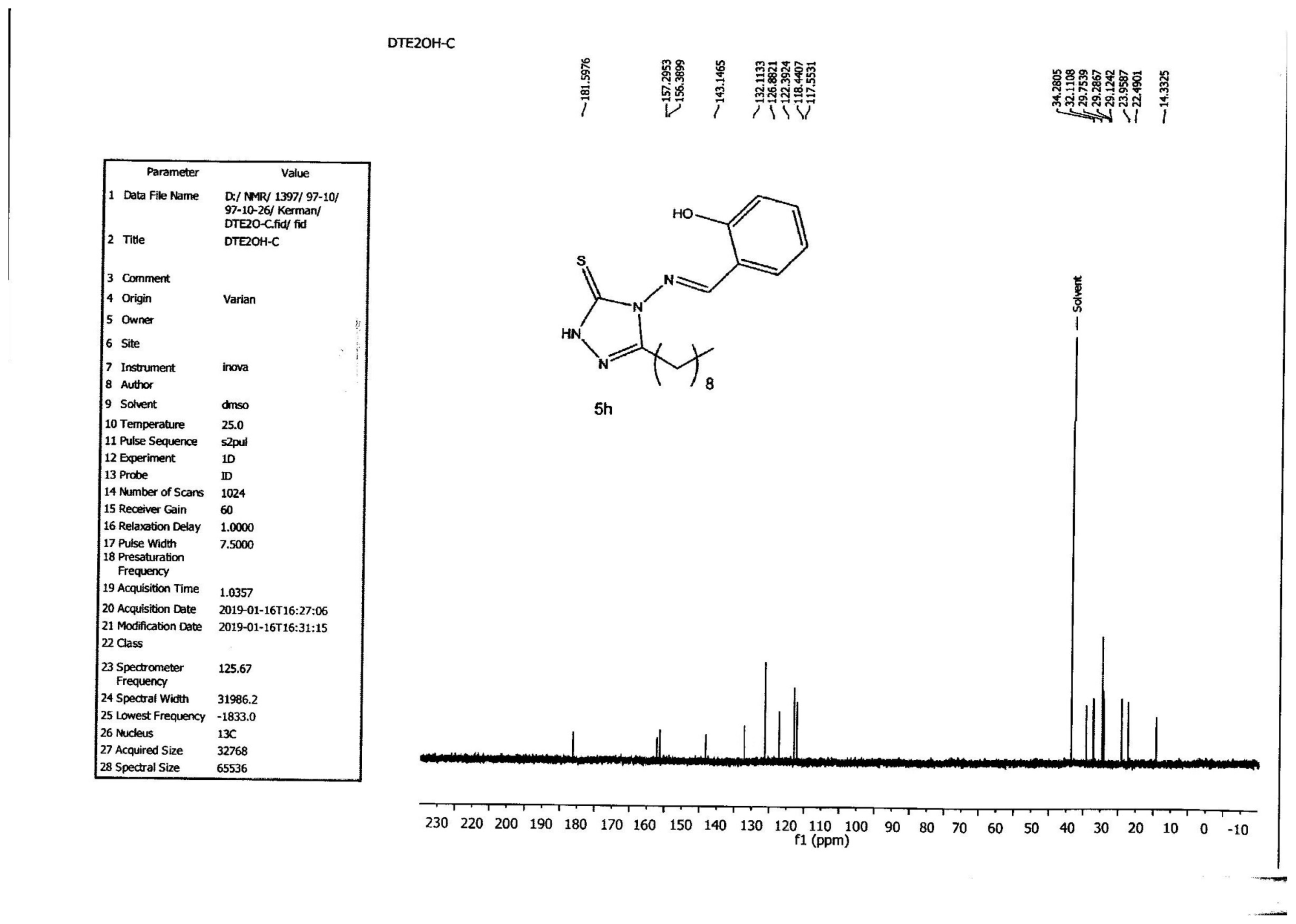

Supplement: Supplementary file 43 [file turkjchem-45-6-1805s43.tif]

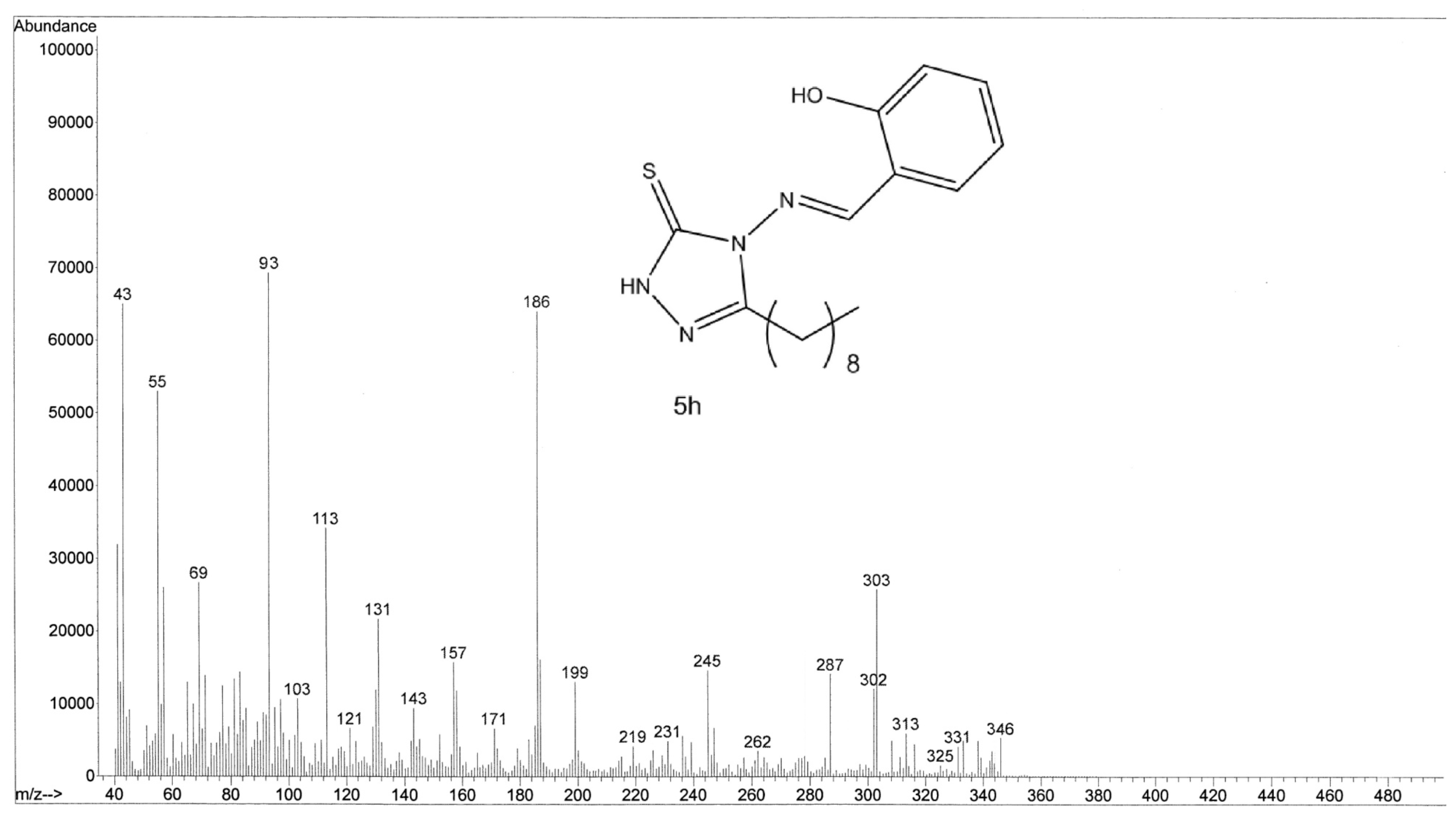

Supplement: Supplementary file 44 [file turkjchem-45-6-1805s44.tif]

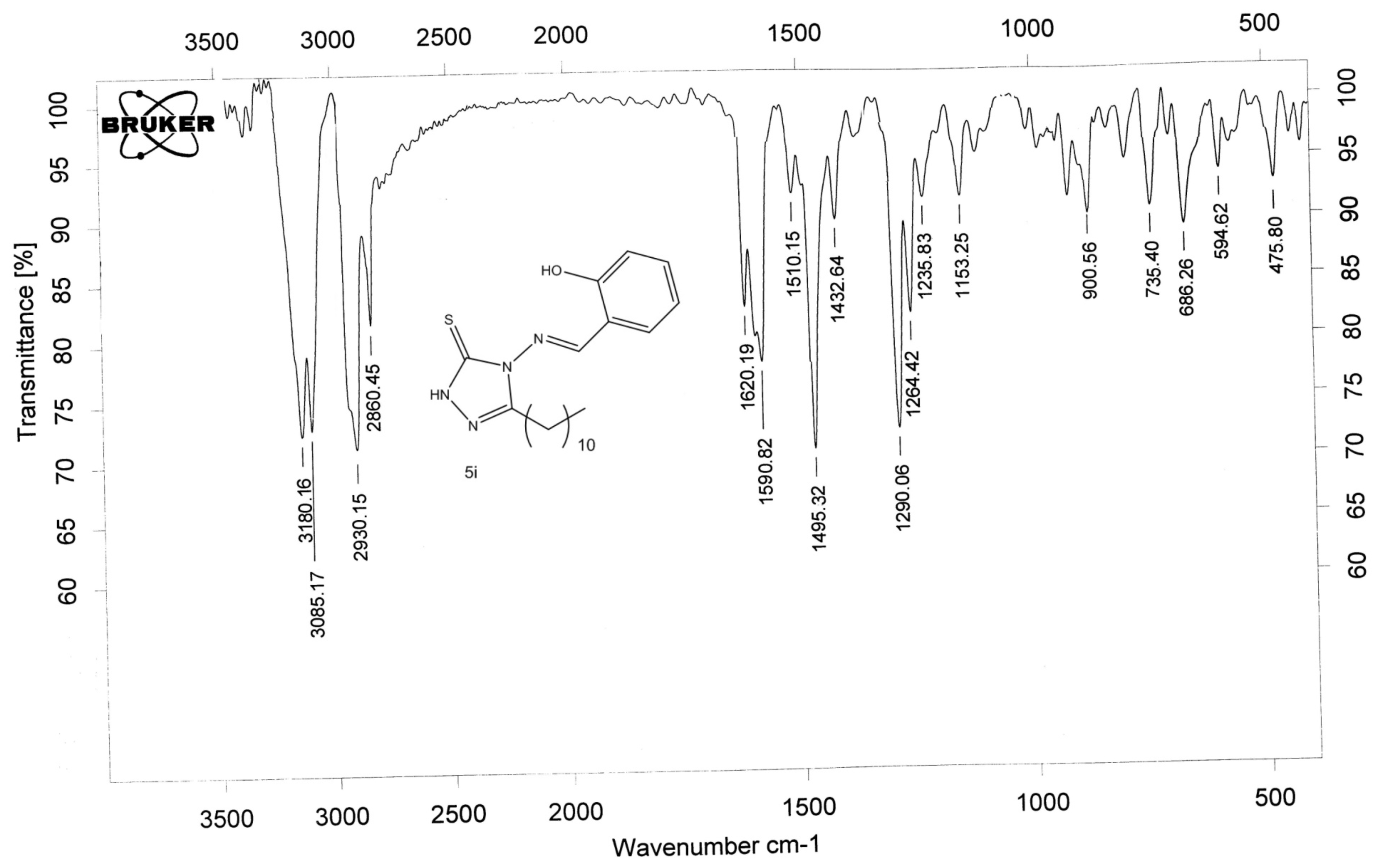

Supplement: Supplementary file 45 [file turkjchem-45-6-1805s45.tif]

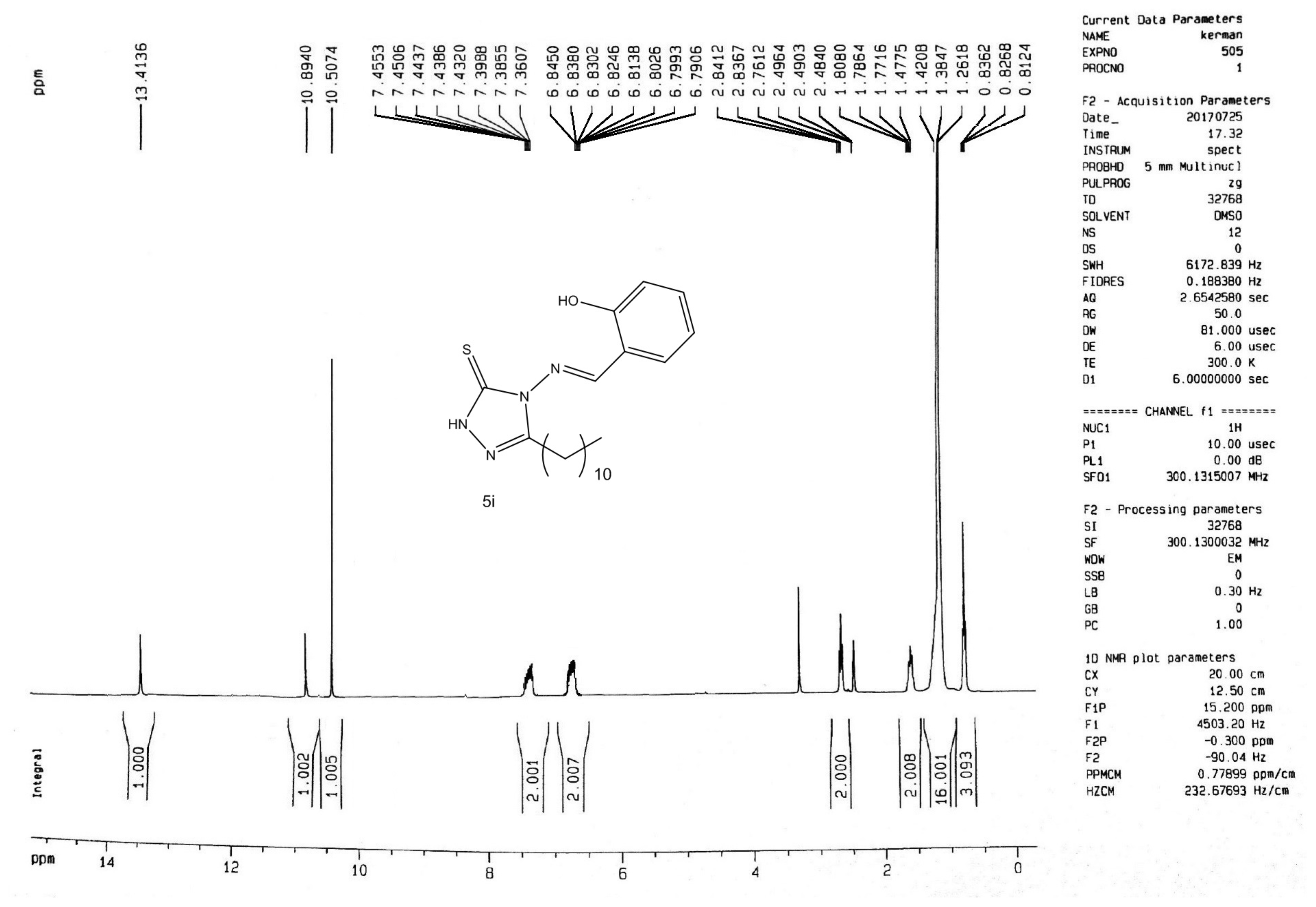

Supplement: Supplementary file 46 [file turkjchem-45-6-1805s46.tif]

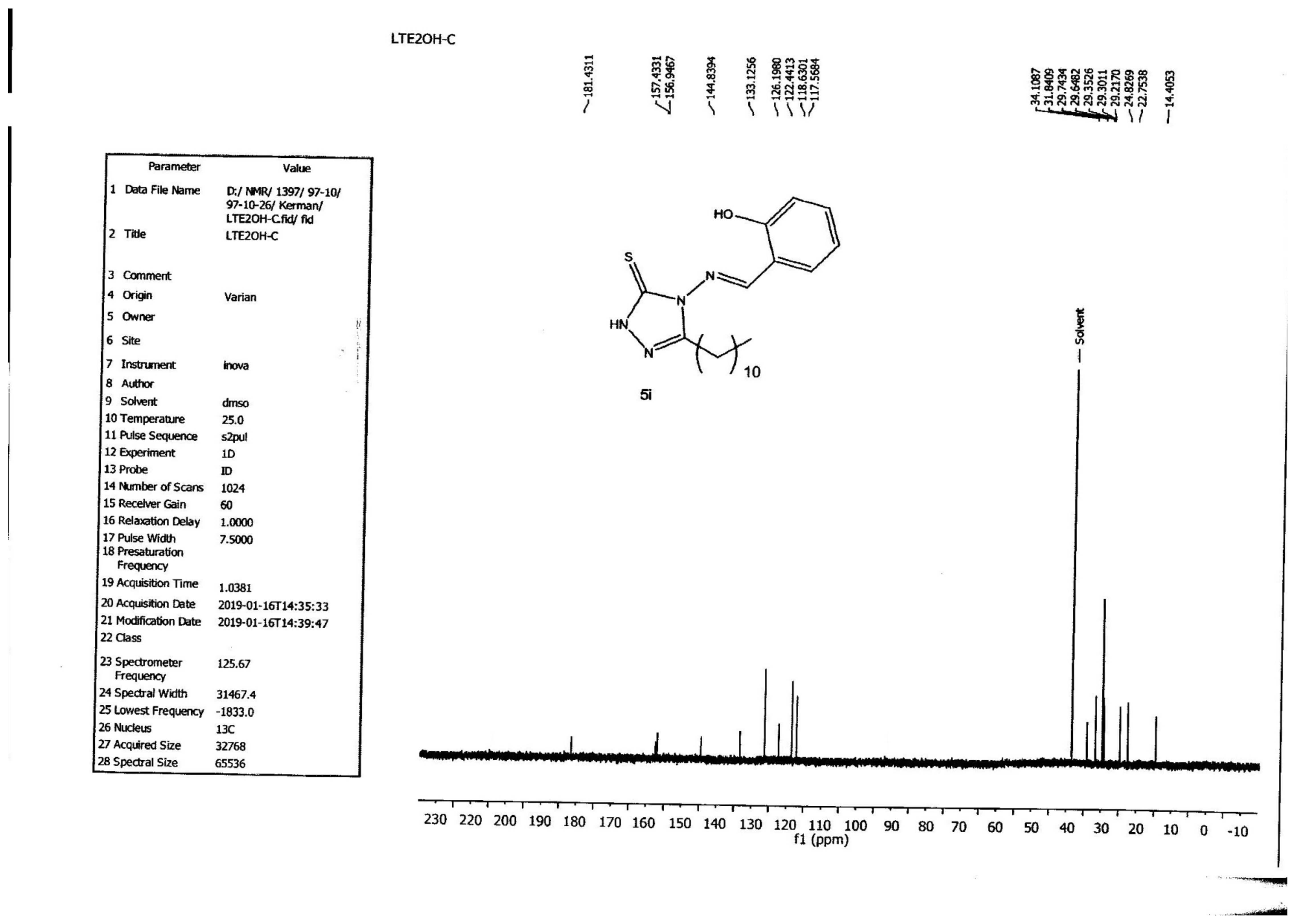

Supplement: Supplementary file 47 [file turkjchem-45-6-1805s47.tif]

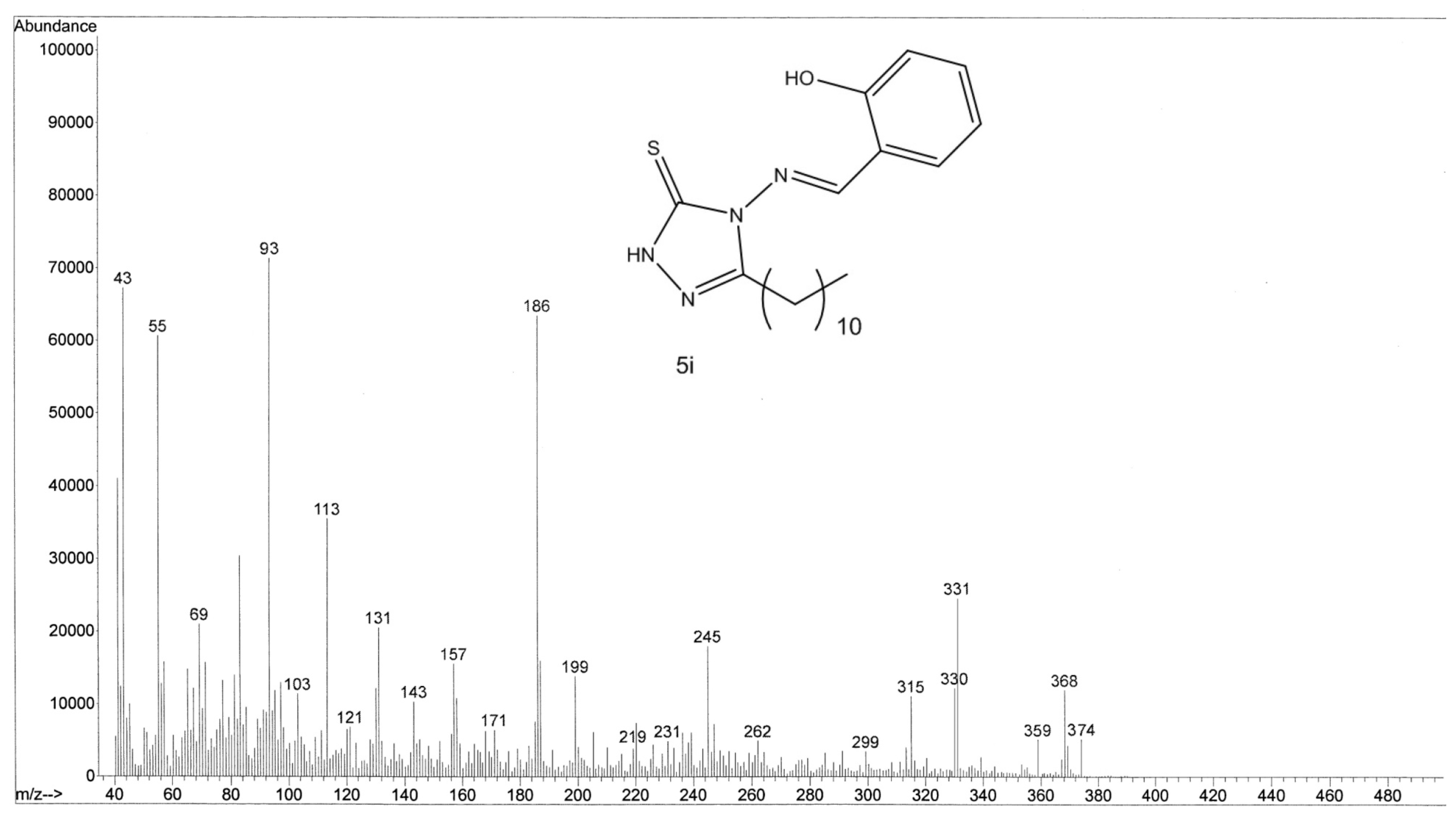

Supplement: Supplementary file 48 [file turkjchem-45-6-1805s48.tif]

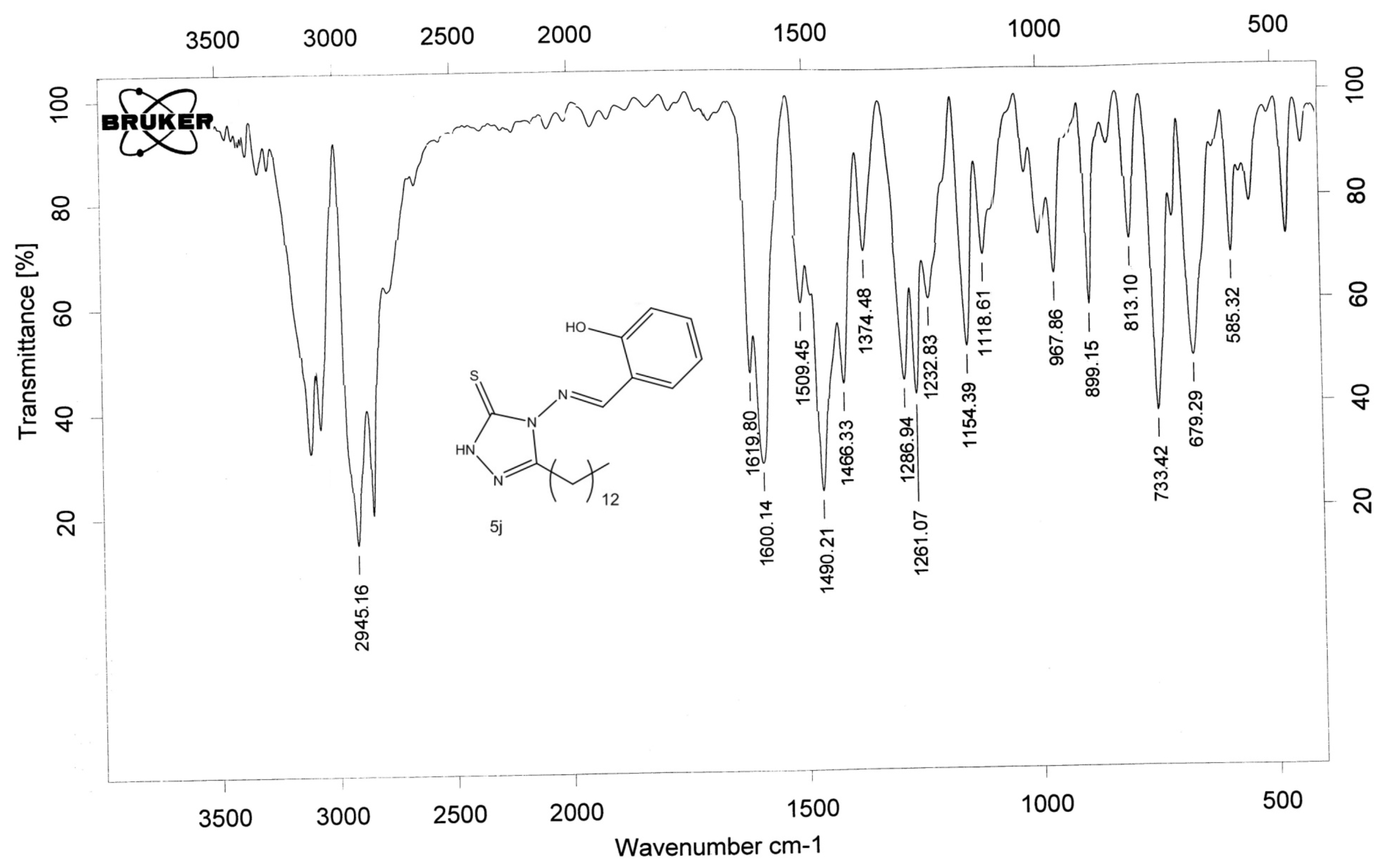

Supplement: Supplementary file 49 [file turkjchem-45-6-1805s49.tif]

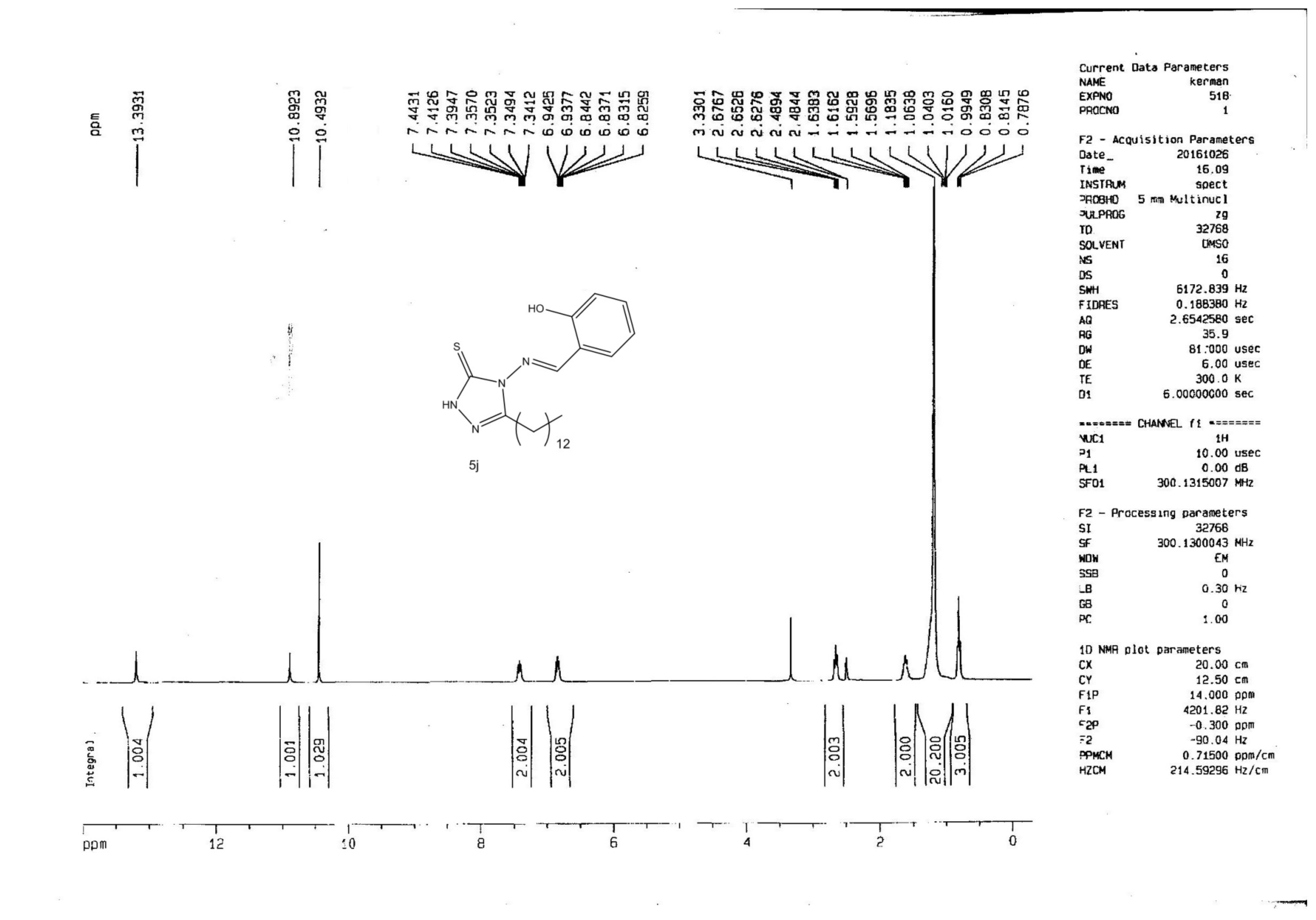

Supplement: Supplementary file 50 [file turkjchem-45-6-1805s50.tif]

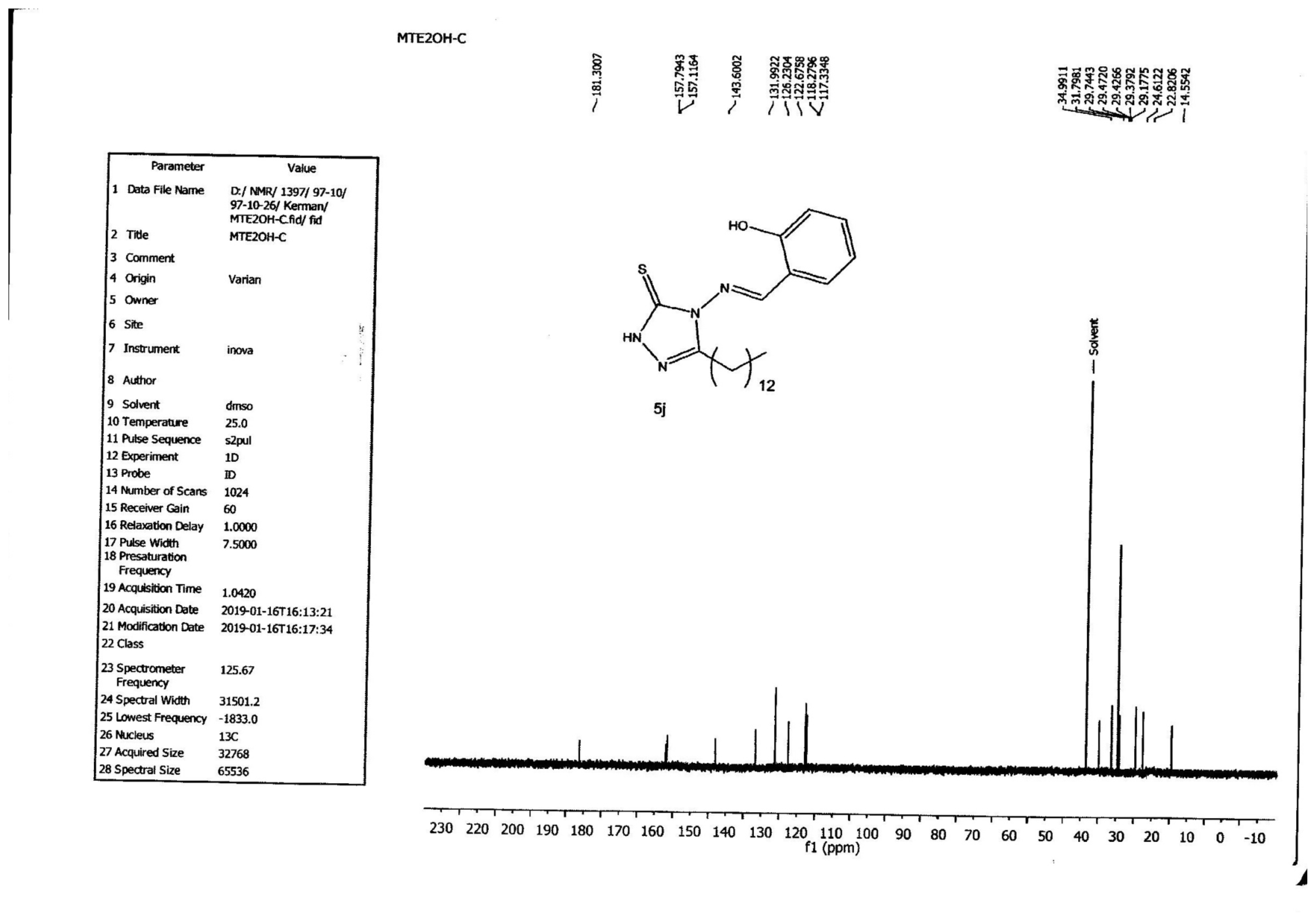

Supplement: Supplementary file 51 [file turkjchem-45-6-1805s51.tif]

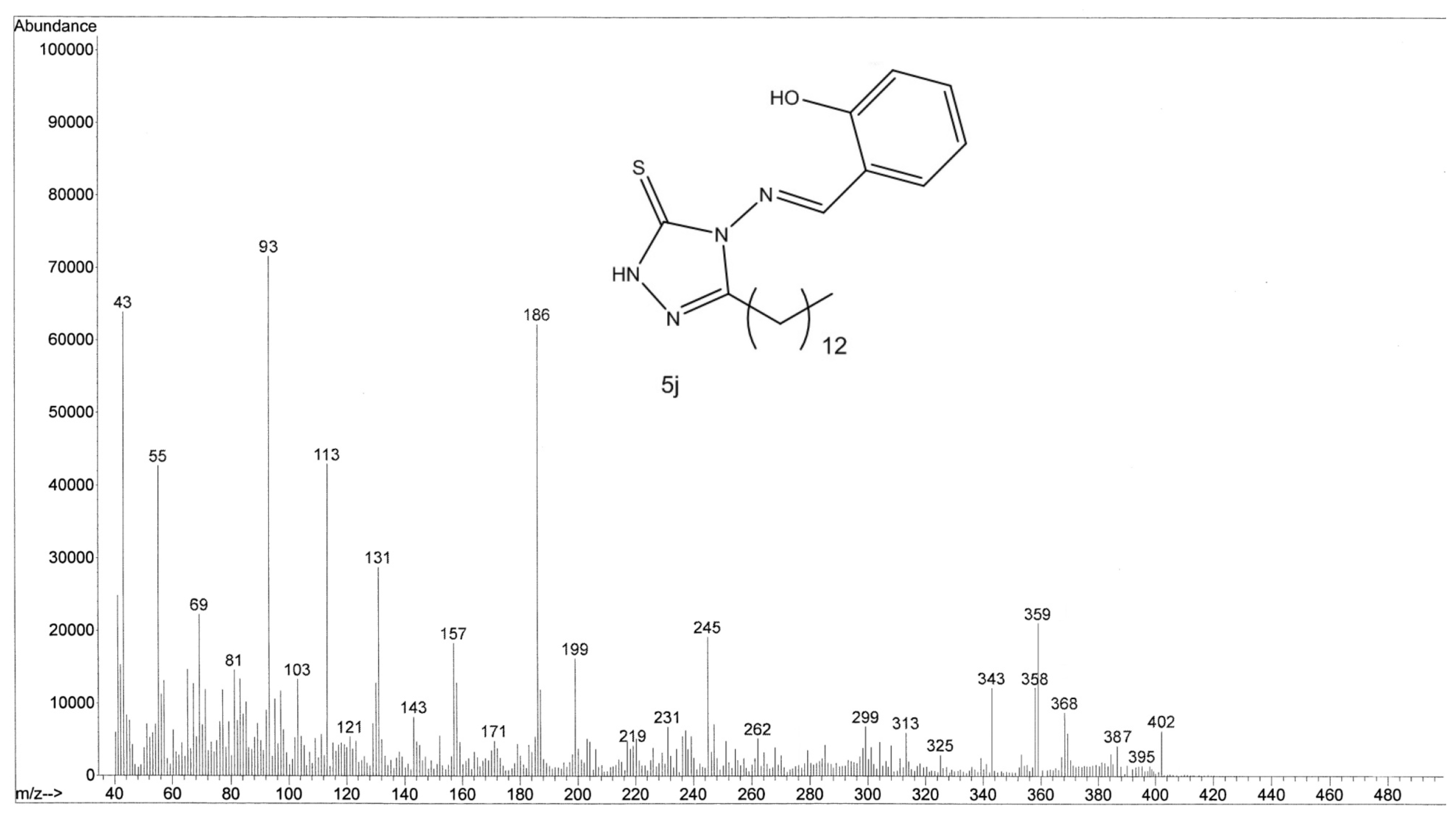

Supplement: Supplementary file 52 [file turkjchem-45-6-1805s52.tif]

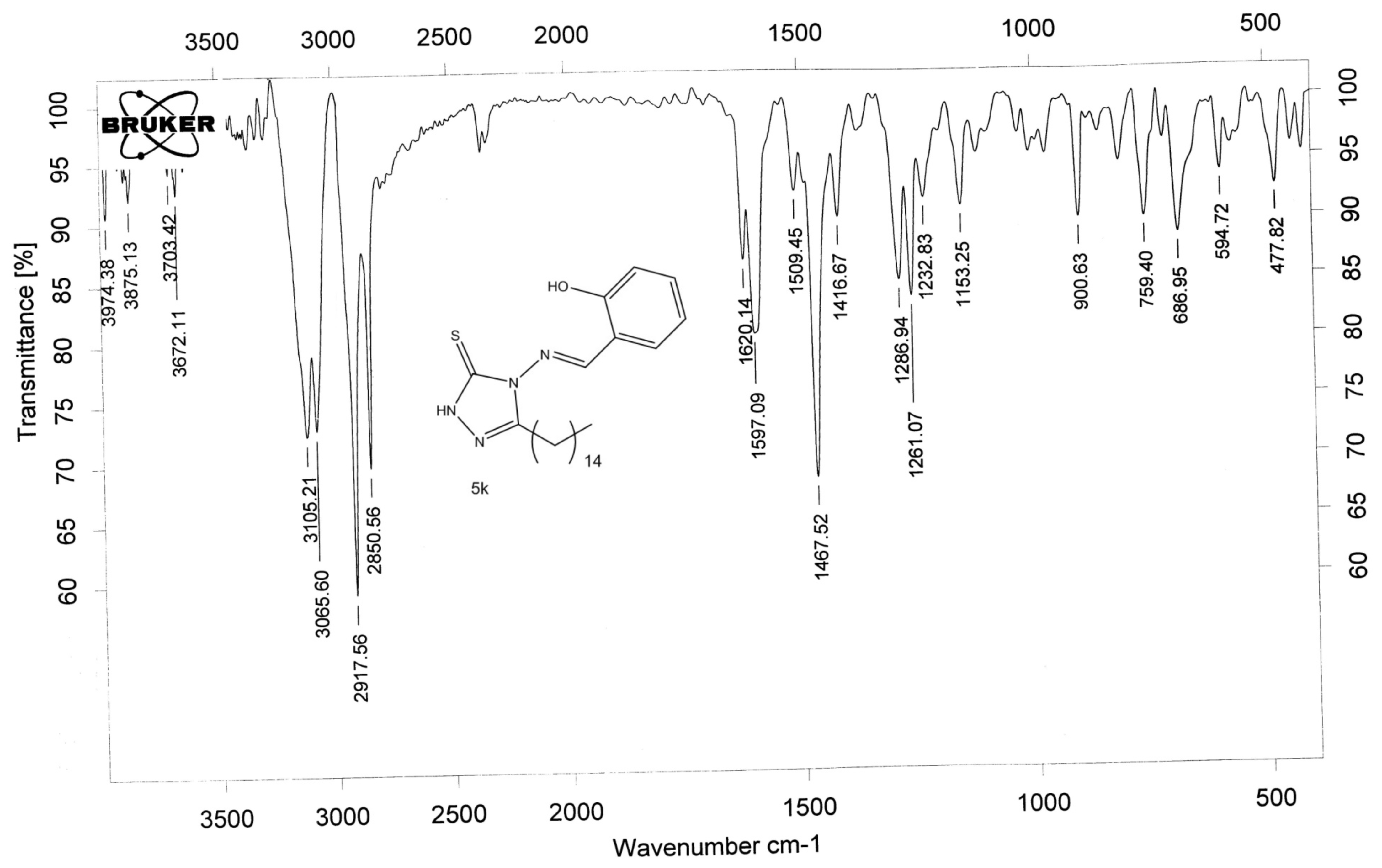

Supplement: Supplementary file 53 [file turkjchem-45-6-1805s53.tif]

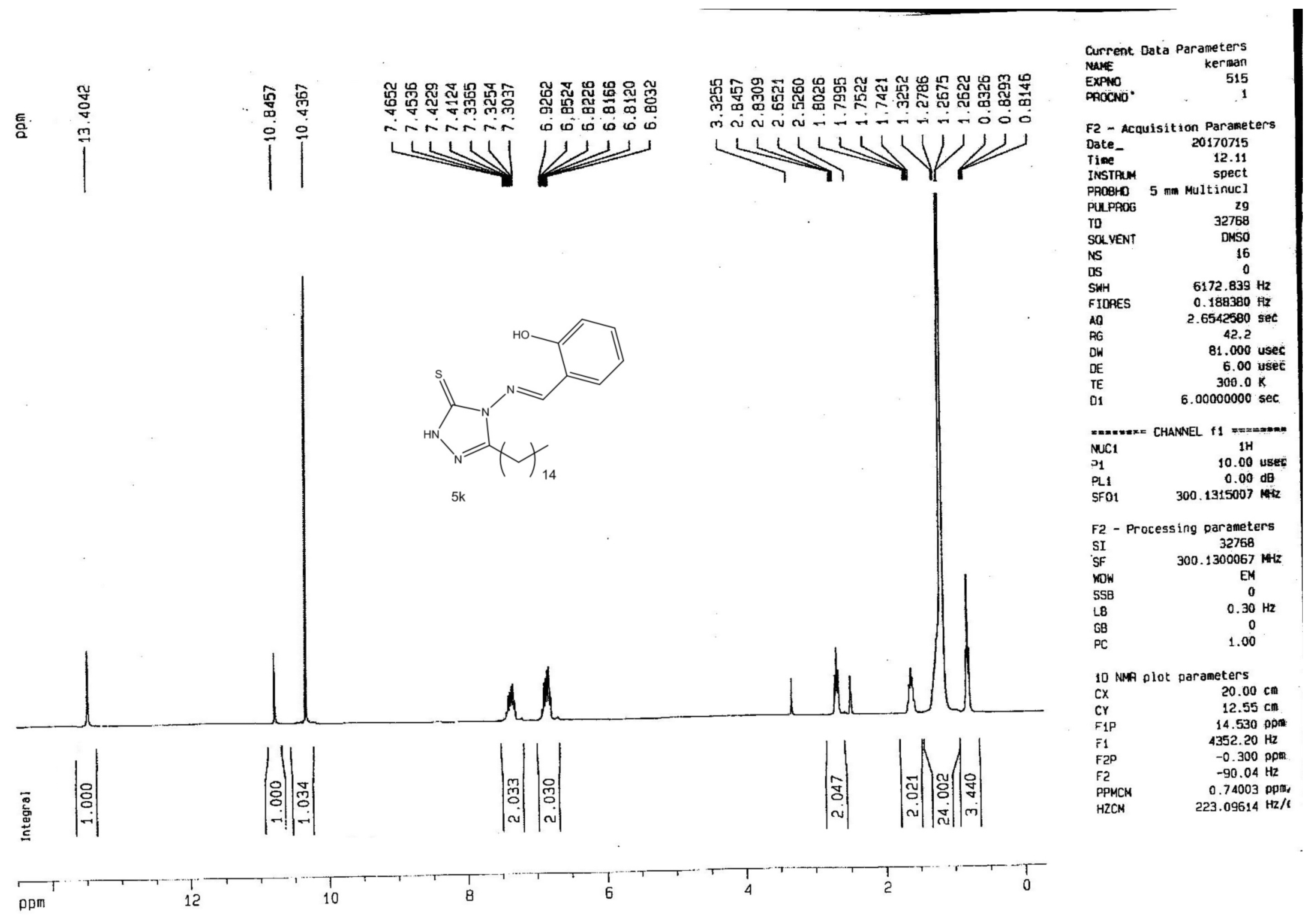

Supplement: Supplementary file 54 [file turkjchem-45-6-1805s54.tif]

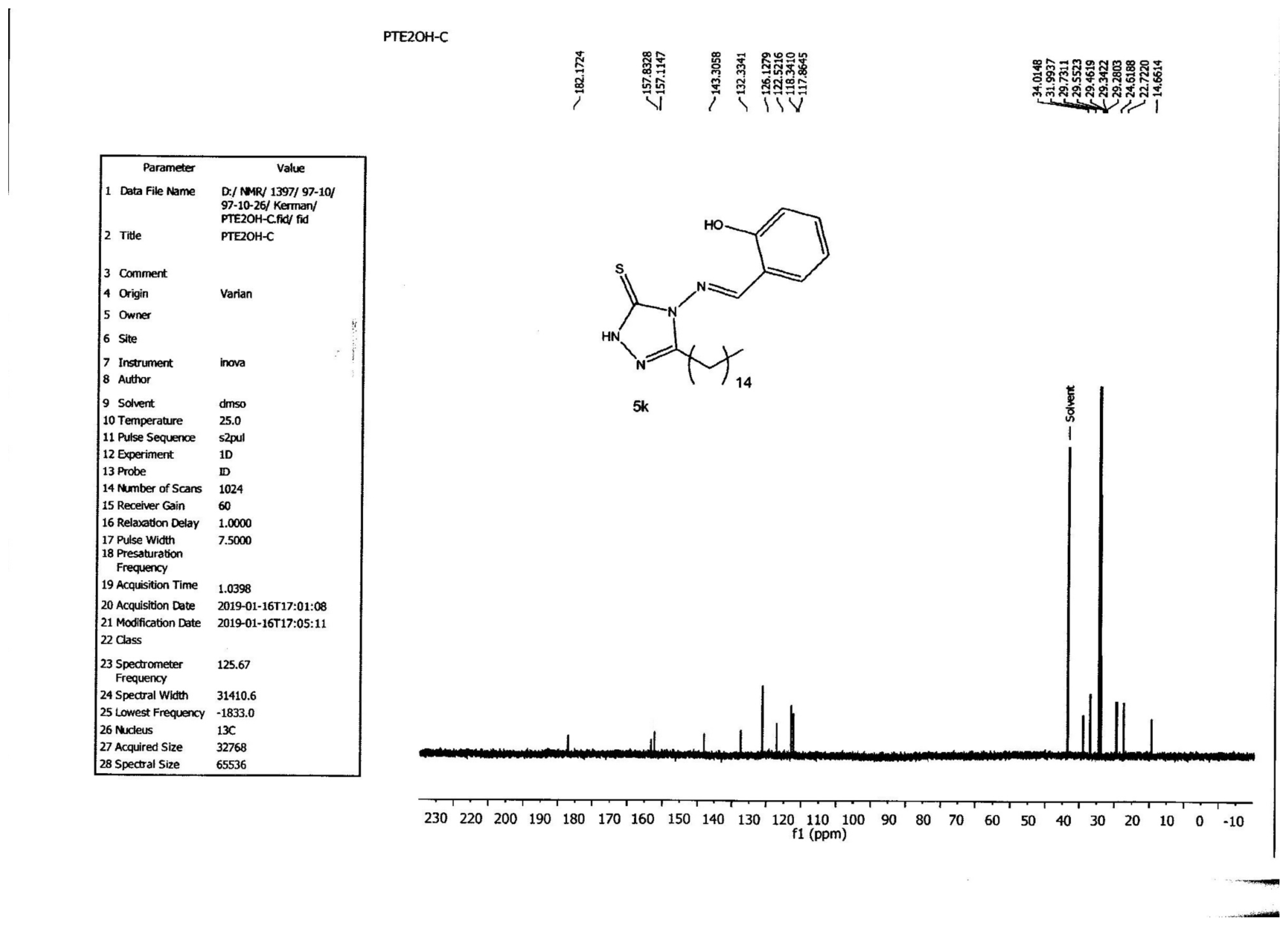

Supplement: Supplementary file 55 [file turkjchem-45-6-1805s55.tif]

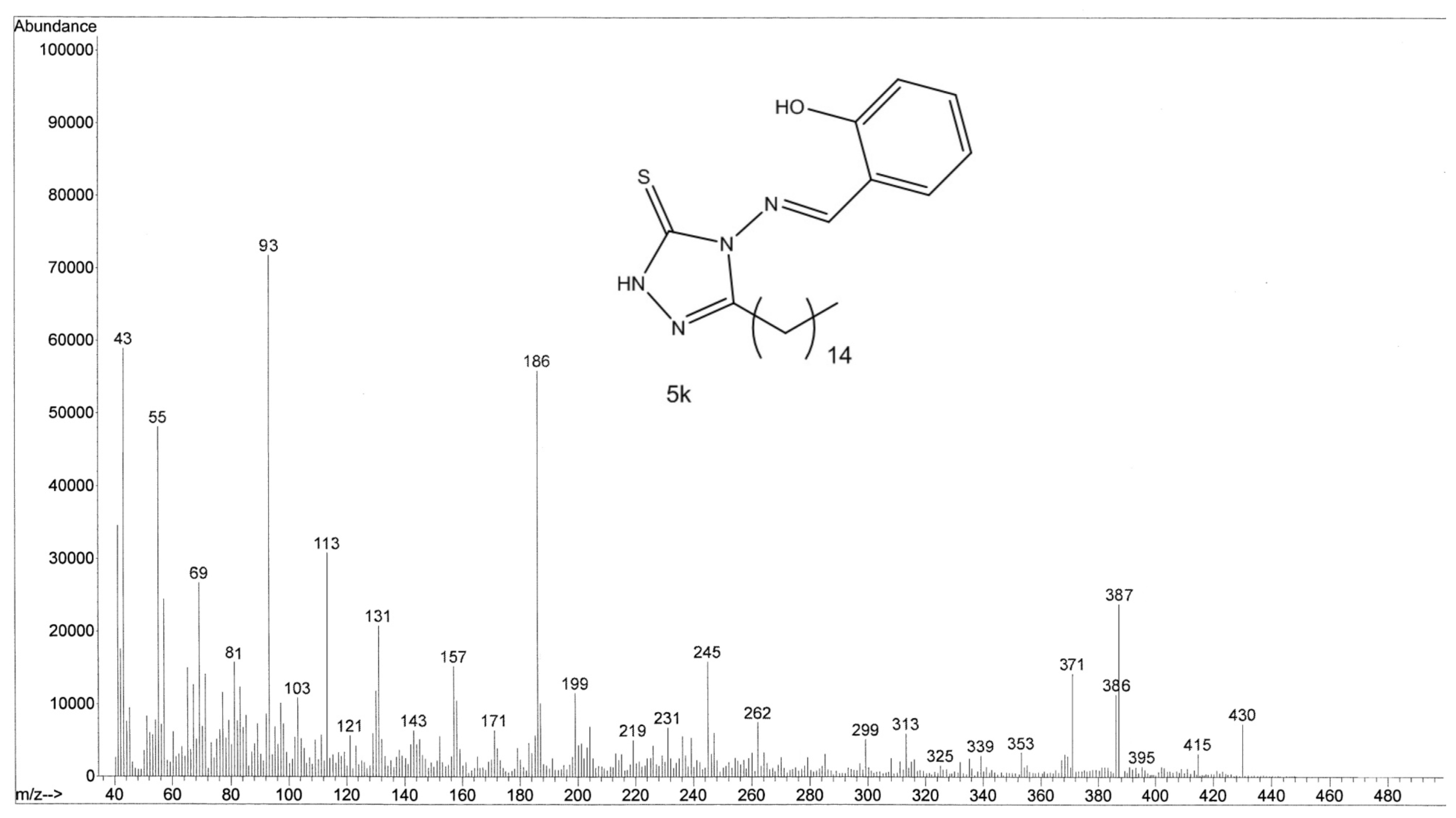

Supplement: Supplementary file 56 [file turkjchem-45-6-1805s56.tif]

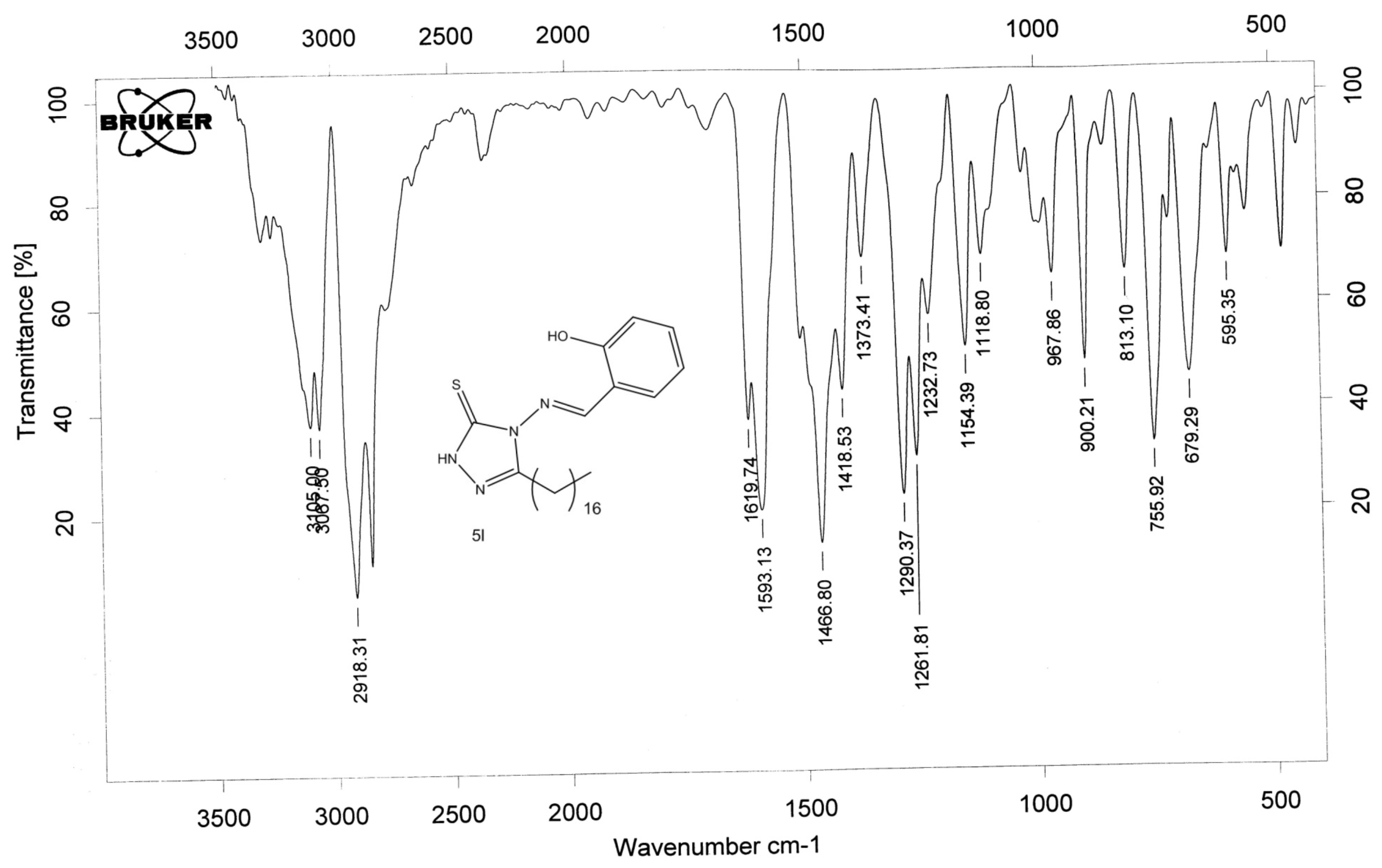

Supplement: Supplementary file 57 [file turkjchem-45-6-1805s57.tif]

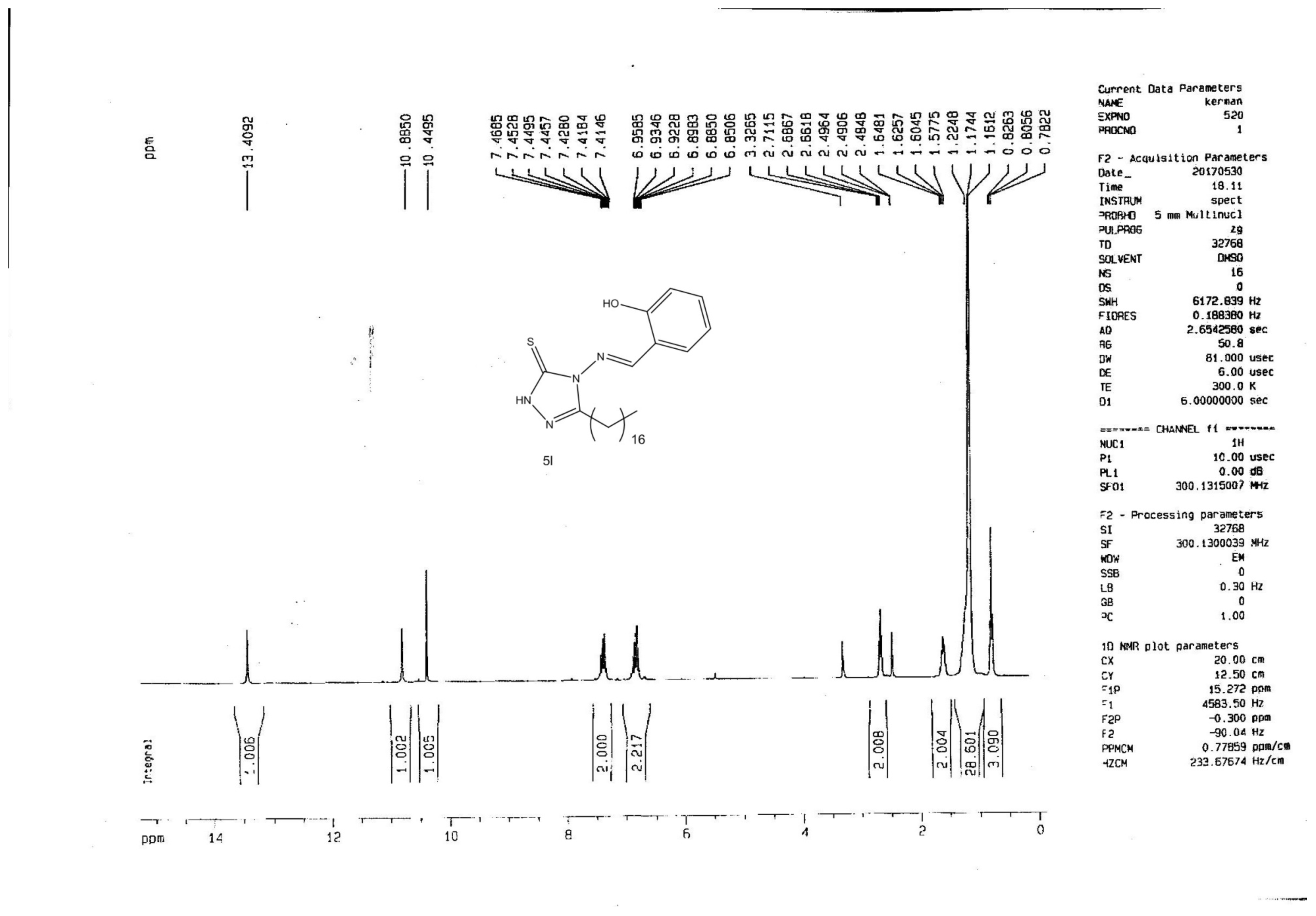

Supplement: Supplementary file 58 [file turkjchem-45-6-1805s58.tif]

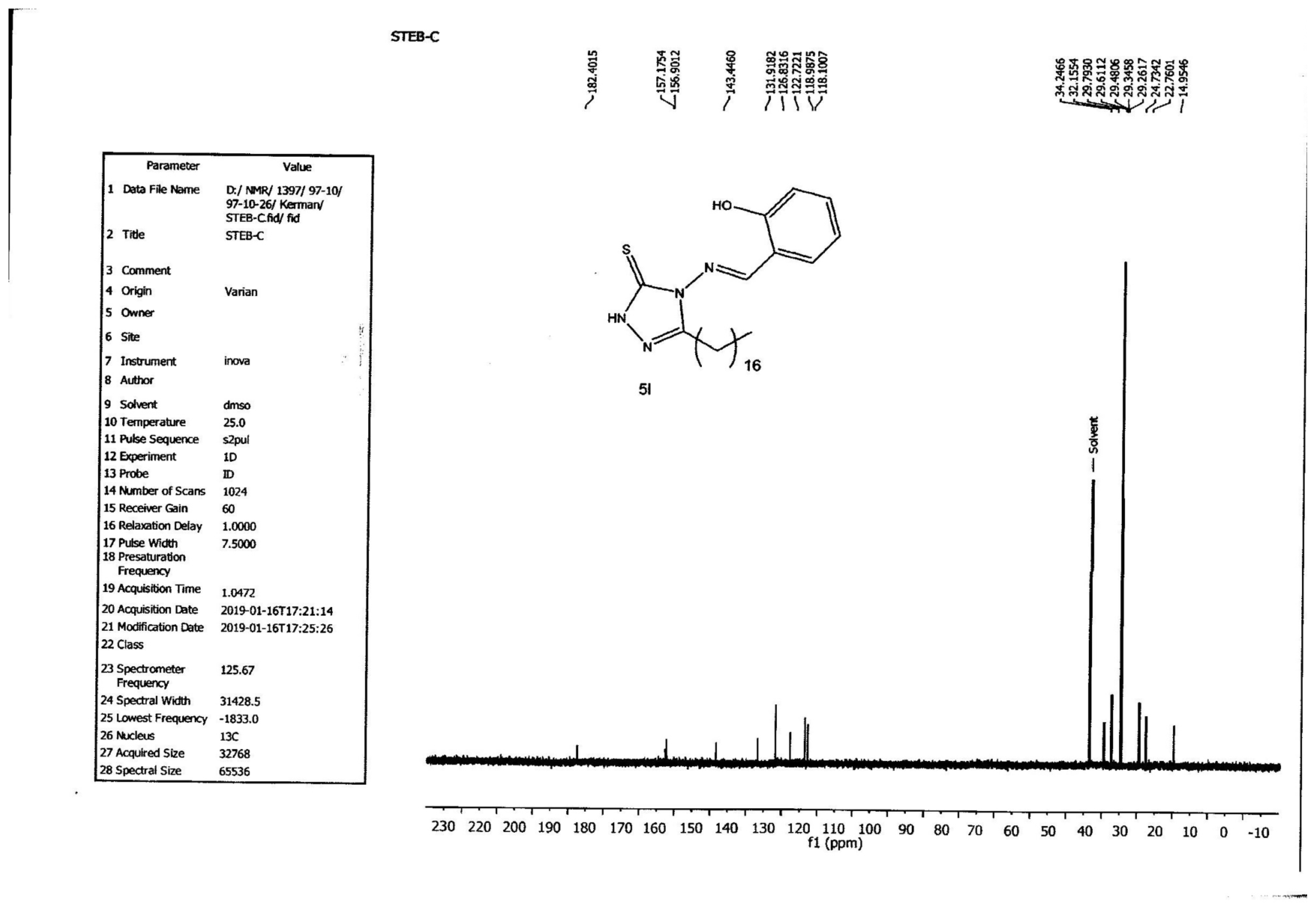

Supplement: Supplementary file 59 [file turkjchem-45-6-1805s59.tif]

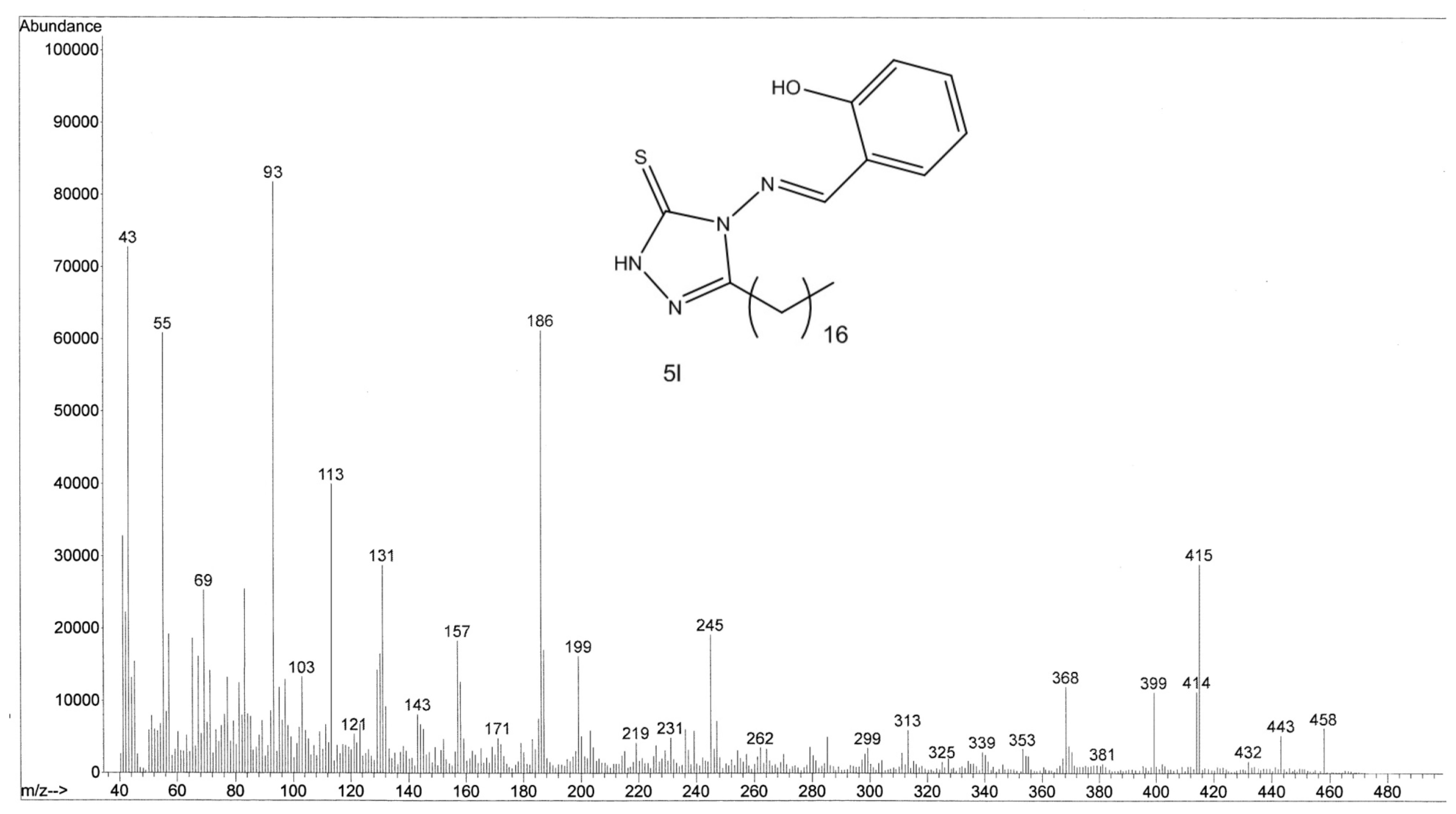

Supplement: Supplementary file 60 [file turkjchem-45-6-1805s60.tif]
